# Recent advances in synthesis and application of Magnéli phase titanium oxides for energy storage and environmental remediation

**DOI:** 10.1039/d4sc04477k

**Published:** 2025-01-20

**Authors:** S. Amanda Ekanayake, Haoxin Mai, Dehong Chen, Rachel A. Caruso

**Affiliations:** a Applied Chemistry and Environmental Science, School of Science, STEM College, RMIT University Melbourne Victoria 3000 Australia rachel.caruso@rmit.edu.au; b Current Address College of Materials Science and Engineering, Qingdao University of Science and Technology Qingdao 266042 P. R. China

## Abstract

High-temperature reduction of TiO_2_ causes the gradual formation of structural defects, leading to oxygen vacancy planar defects and giving rise to Magnéli phases, which are substoichiometric titanium oxides that follow the formula Ti_*n*_O_2*n*−1_, with 4 ≤ *n* ≤ 9. A high concentration of defects provides several possible configurations for Ti^4+^ and Ti^3+^ within the crystal, with the variation in charge ordered states changing the electronic structure of the material. The changes in crystal and electronic structures of Magnéli phases introduce unique properties absent in TiO_2_, facilitating their diverse applications. Their exceptional electrical conductivity, stability in harsh chemical environments and capability to generate hydroxyl radicals make them highly valuable in electrochemical applications. Additionally, their high specific capacity and corrosion resistance make them ideal for energy storage facilities. These properties, combined with excellent solar light absorption, have led to their widespread use in electrochemical, photochemical, photothermal, catalytic and energy storage applications. To provide a complete overview of the formation, properties, and environmental- and energy-related applications of Magnéli phase titanium suboxides, this review initially highlights the crystal structure and the physical, thermoelectrical and optical properties of these materials. The conventional and novel strategies developed to synthesise these materials are then discussed, along with potential approaches to overcome challenges associated with current issues and future low-energy fabrication methods. Finally, we provide a comprehensive overview of their applications across various fields, including environmental remediation, energy storage, and thermoelectric and optoelectronic technologies. We also discuss promising new directions for the use of Magnéli phase titanium suboxides and solutions to challenges in energy and environment-related applications, and provide guidance on how these materials can be developed and utilised to meet diverse research application needs. By making use of control measures to mitigate the potential hazards associated with their nanoparticles, Magnéli phases can be considered as versatile materials with potential for next generation energy needs.

## Abstract 2

Review covering the formation, properties and applications of Magnéli phase titanium suboxides (Ti_*n*_O_2*n*−1_, 4 ≤ *n* ≤ 9) in energy storage and environmental remediation.

## Graphical abstract

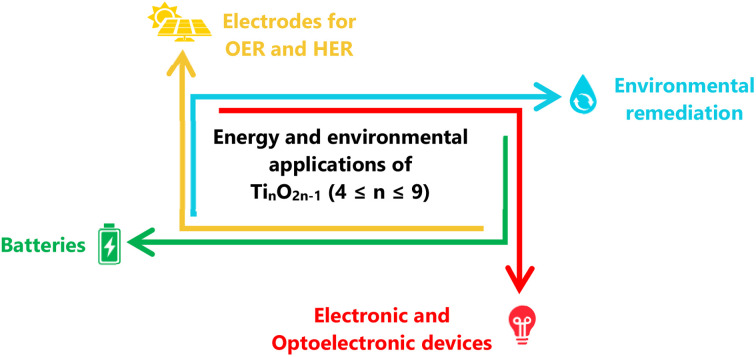

## 1. Introduction

Titanium dioxide (TiO_2_) is one of the most widely used semiconductors in industry owing to its high abundance, low cost, non-toxicity, chemical stability and biocompatibility.^1–4^ These properties have made TiO_2_ an excellent candidate for utilisation in a variety of diverse applications including optics, cosmetics, photovoltaic devices, photocatalysis and photoelectrochemical cells.^2,4^ However, the large bandgap of TiO_2_ (3.2 eV for anatase), which confines its light absorbance to the UV region and the high rate of electron–hole recombination, greatly limits its potential to be used in many other applications.^5^ Various approaches have been investigated to extend the light absorption of TiO_2_ into the visible region, including surface modification using organic materials, semiconductor coupling, creation of surface defects and oxygen vacancies (V_O_), non-metal doping and transition metal doping.^6–8^ Recent studies on defective titania have shown that the changes in physical and chemical properties of TiO_2_ such as electrical, thermal, optical and tribological properties can enhance its performance towards electrocatalytic and photocatalytic applications.^9,10^

The introduction of defects in the lattice structure of TiO_2_ serves as a platform to tune its physicochemical and surface properties, making it useful in a wider range of applications. These defects can be produced by thermal annealing, electron bombardment, sputtering and *via* the inclusion of impurities such as Ca and H.^11^ Thermal annealing of TiO_2_ produces its reduced oxide form, due to the formation of V_O_ and Ti interstitials. Low amounts of these vacancies (<10^−4^) are considered as point defects within the structure, whereas higher degrees of reduction form interstitial defects along with V_O_. Increasing the amount of V_O_ during defect creation changes the O/Ti ratio in the TiO_2_ crystal structure, leading to the generation of titanium suboxides.^12^ At elevated temperatures, these V_O_ arrange into planar defects known as crystallographic shear planes (CSPs). As the concentration of CSPs increases, the planes arrange into regular arrays known as Magnéli phases, with chemical formula Ti_*n*_O_2*n*−1_, where *n* can range from 4 to 9.^12–14^ Hence, the structure of Magnéli phases can be visualised as rutile chains, consisting of *n* number of unmodified octahedral blocks, interrupted by a CSP.^15–17^

Formation of V_O_, or its singly (V_O_^+^) or doubly (V_O_^2+^) charged components, result in delocalised electrons within the crystal structure that improve the electrical conductivity of the defective material.^12^ Hence, since their discovery by Arne Magnéli in 1957,^18^ Magnéli phase titanium suboxides have been widely used in the fields of electronics, photocatalysis, tribology, optoelectronics and thermomechanics due to their desirable features including excellent electrical and thermal conductivity, visible light absorptivity and chemical inertness.^14,19–21^ Magnéli phase titanium suboxides are used in various applications due to their unique structure, which imparts exceptional properties such as high electrical conductivity and strong corrosion resistance. For instance, Ti_4_O_7_, the most studied Magnéli phase, has an electrical conductivity of ∼1000 S cm^−1^, surpassing that of graphitic carbon (∼727 S cm^−1^).^22^ This high conductivity, combined with their oxygen evolution potential, makes Magnéli phases ideal for electrochemical applications like water splitting and cathodic protection.^22^ Additionally, Magnéli phases (*n* = 4–6) have been commercialised under the trade name Ebonex® and are utilised as electrode substrates in batteries and as catalyst supports in fuel cells and water treatment systems.^23,24^ Their stability in harsh chemical environments, such as fluoride-based etchants, hydrochloric acid and aqua regia, further expands their versatility in diverse electrochemical environments.^25^ Applications such as reactive electrochemical membranes (REMs) also take advantage of the material's ability to generate hydroxyl radicals (OH˙) through water oxidation.^26^ In battery applications, Magnéli phases are highly valued due to their excellent electrical conductivity and high specific capacity, allowing for significant energy storage and release. Their stability, corrosion resistance, and durability make them reliable materials for rechargeable batteries.^27,28^ In optics and photosensitivity-related applications, these suboxides are used for their visible light and near-infrared (NIR) photosensitivity, which results from V_O_ created during the phase transformation from rutile to Magnéli.^29^ Furthermore, their high solar-to-vapour efficiency makes them suitable for photothermal applications, such as solar steam generation.^30^ These properties make Magnéli phases highly versatile, supporting their use in diverse fields, including energy storage, water treatment and solar energy harvesting.

Throughout the last few decades, studies have been conducted on various synthesis approaches to produce different types of titanium suboxides and examine their application in a range of areas, including for environmental remediation, energy generation or in optoelectronic devices. These aspects as well as the changes in TiO_2_ surface upon defect creation and their effect in catalytic applications have been previously reviewed.^11,14,31–33^ Although several reviews have focused on the literature related to titanium suboxides and defective (or “black”) TiO_2_,^31,34–37^ only a few have specifically addressed the synthesis and potential applications of Magnéli phase titanium suboxides.^33,38,39^ Some of these reviews not only discuss the ‘Magnéli phases’ but also provide a comprehensive overview of the synthesis and properties of all titanium oxides up to TiO, describing their structure, synthesis and performance. However, these reviews primarily focus on either the environmental remediation or electrochemical applications of Magnéli phase titanium suboxides, leaving their broader potential in areas such as thermoelectric and energy storage applications underexplored. To address this gap, this review provides a comprehensive overview of the literature on Magnéli phase titanium suboxides. We discuss the formation, synthesis and properties of the lower Magnéli phases (Ti_*n*_O_2*n*−1_, 4 ≤ *n* ≤ 9), along with recent advances (within the timeframe 2015–2024) in various fields, including environmental remediation, thermoelectric applications, energy storage and catalysis. In discussing the use of these materials across diverse applications, we explore the fundamental principles and mechanisms that underlie their performance, drawing connections among applications to guide readers in tailoring Magnéli phase titanium suboxides for specific uses. This review also highlights the challenges associated with their application in various fields, offering insights into potential future developments in the lesser-explored areas of Magnéli phase titanium suboxides.

## 2. Structure and properties of Magnéli phase titanium suboxides

### 2.1 Crystal structure

#### 2.1.1 Types of defects on the crystal structure of TiO_2_

**Fig. 1:**
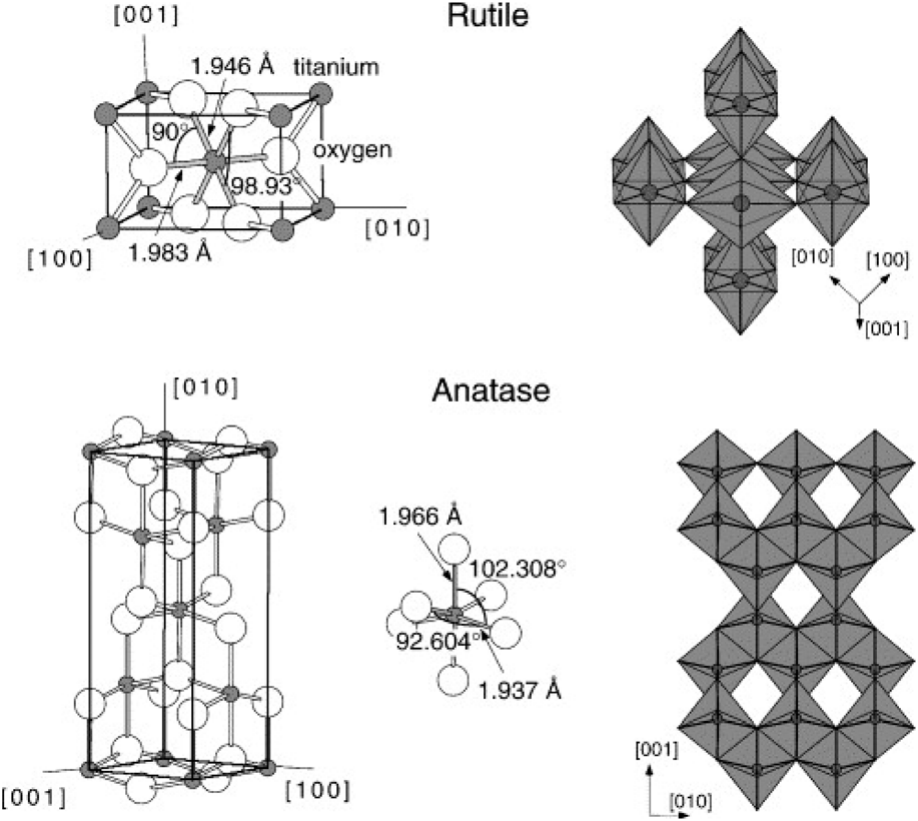
Bulk structures of rutile and anatase TiO_2_. Reproduced with permission from ref. 11. Copyright 2003, Elsevier.

TiO_2_ crystallises in three main phases: anatase (tetragonal, *D*_4h_^19^-*I*4_1_/*amd*, *a* = *b* = 3.782 Å, *c* = 9.502 Å), rutile (tetragonal, *D*_4h_^14^-*P*4_2_/*mnm*, *a* = *b* = 4.584 Å, *c* = 2.953 Å) and brookite (rhombohedral, *D*_2h_^15^-*Pbca*, *a* = 5.436 Å, *b* = 3.782 Å, *c* = 5.135 Å).^11^ Anatase and rutile phases have been the most investigated phases of TiO_2_. In both crystals, the building blocks of their unit cells are made of slightly distorted octahedra, composed of a titanium atom surrounded by six oxygen atoms (Fig. 1). Additionally, cotunnite, a high-pressure phase of TiO_2_, prepared at high temperature and pressure has also been reported and is considered as one of the hardest polycrystalline materials.^40^ However, rutile is identified as the thermodynamically stable phase of TiO_2_, stable at all temperatures, while other phases are considered metastable.^11,41^

Intentional creation of defects in TiO_2_ with precise control of their position, concentration and distribution tailors the materials physicochemical properties and reactivity. Different types of defects introduced into TiO_2_ can be classified as follows.^31,42,43^

(I) Zero dimensional defects (point defects: oxygen and titanium vacancies and interstitials and substitutional impurities).

(II) One dimensional defects (line defects: edge or screw dislocations).

(III) Two dimensional defects (planar defects: grain and twin boundaries and stacking faults).

(IV) Three dimensional defects (volume defects: aggregates of atoms or vacancies to form precipitates or voids).

Defects in the TiO_2_ lattice can be introduced either by changing the O/Ti ratio or through the incorporation of high and low valence ions into the lattice to form donors and acceptors. These defects in titania can be generated during synthesis or post synthesis of the material.^31,44^ Some approaches used to induce defects in the TiO_2_ lattice include thermal annealing, electron bombardment, prolonged oxidation, partial oxidation, reducing agents (H_2_, C, metals), UV irradiation, high energy particle bombardment and vacuum activation.^11^ Calculated formation energies of point defects have shown that Ti-rich conditions favour the formation of V_O_ and Ti interstitials, which behave as weak donors, whereas O-rich conditions lead to Ti vacancies that act as acceptors.^45^ Defect disorder in TiO_2_ as a variation of oxygen partial pressure was elucidated by Nowotny and co-workers. These findings suggested that Ti interstitials and electrons are formed at low oxygen partial pressures due to reduction of TiO_2_, whereas prolonged oxidation forms Ti vacancies and holes. In addition, due to the low formation enthalpy of V_O_ and their prevalence across a broad range of stoichiometry in both reducing and oxidizing conditions, V_O_ can be regarded as the dominant type of defect in TiO_2_.^44^

V_O_ in metal oxides can be generated through various processes, such as thermal treatment in a vacuum or inert environment, chemical reduction at elevated temperatures, ion doping or interfacial engineering. When metal oxides undergo high-temperature treatment, lattice oxygen atoms may either desorb to release O_2_ (eqn (1)) or react with H_2_ or CO, forming H_2_O and CO_2_, respectively.^46^1
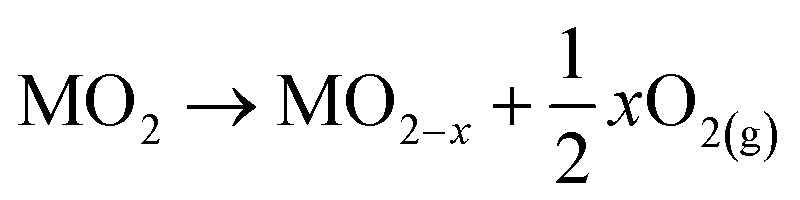


V_O_ are the most common point defects in semiconducting metal oxides. This is due to their lowest formation energy among various types of defects that act as donors.^47,48^ Density functional theory (DFT) calculations indicate that the formation of V_O_ introduces a defect level near the Fermi level, enhancing photoabsorption and electronic conductivity while reducing electron–hole recombination, which in turn extends the lifetimes of charge carriers. Additionally, V_O_ can create unsaturated coordination that are more reactive, facilitating processes such as H_2_ evolution and CO_2_ reduction.^46^

By modifying the local atomic and electronic structures, V_O_ act as centres for charge carrier separation, enhancing carrier separation efficiency. They can also adjust light absorption, influence the conductivity, and impact separation and surface reactions in semiconducting metal oxides.^46,49^ This results in changes in their chemical and physical properties including photocatalytic and photoelectrochemical effects, superconductivity, ferromagnetism, piezoelectric effect, redox activity and phase transitions.^48^ In addition, research has shown that these vacancies are involved in reactive oxygen species generation and acting as crucial adsorption and active sites in different applications.^50^

#### 2.1.2 Formation of titanium suboxides

##### 2.1.2.1 Structural changes occurring during defect creation

**Fig. 2:**
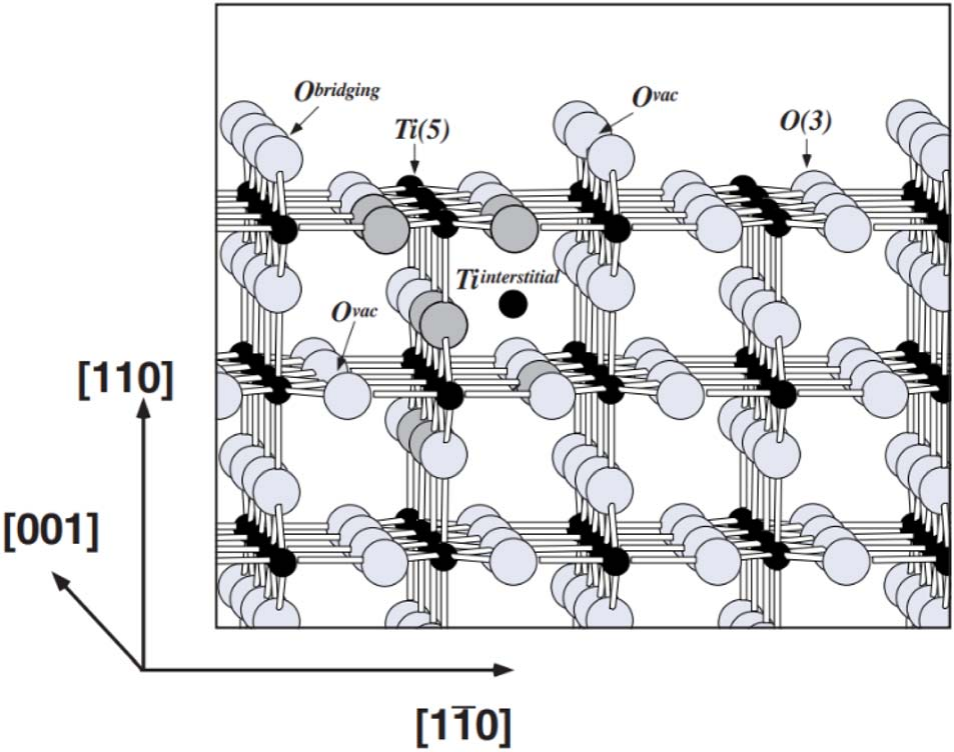
Ball and stick model of the rutile TiO_2_ (110) surface. Large grey balls represent oxygen atoms and small black balls represent titanium atoms. Two types of point defects that are prevalent in rutile TiO_2_, oxygen vacancies (O_vac_) and Ti interstitials, are shown. Reprinted with permission from ref. 51. Copyright 2003, Springer Nature.

As shown in Fig. 2, in the rutile crystal lattice structure, each Ti atom is surrounded by six O neighbours and four Ti next-nearest neighbours while each O atom is bonded to 3 Ti atoms. Within rutile TiO_2_, the (110) surface is the most stable crystal surface, characterised by rows of titanium and oxygen atoms along the [001] direction.^52^ The titanium atoms on the surface exhibit five-fold coordination with one dangling bond and those in the bulk, six-fold coordination. Oxygen atoms can be present in-plane (bulk) or in bridging positions. Those in bulk are three-fold coordinated whereas the oxygen atoms in bridging positions are two-fold coordinated. Due to less coordination at the surface, these oxygen atoms are prone to removal during high-temperature treatments, leading to the formation of point defects.^51^

During defect creation, surface oxygen atoms in the TiO_2_ lattice can be removed as a H_2_O molecule by a hydrogen reductant or O_2_ molecule by a non-hydrogen reductant, leading to the formation of surface V_O_.^53,54^ These V_O_ can cause reconstruction within the TiO_2_ lattice or if the concentration of V_O_ is very high it can eventually lead to lattice disorder.^55^ An oxygen vacancy is a double donor, where the position of the absent oxygen atom in the lattice can be occupied by two electrons leading to a neutral oxygen vacancy. Resonant photoemission spectroscopy confirms that these electrons have the ability to partially occupy the Ti 3d state, resulting in the creation of an energy state approximately 0.8 eV below the Fermi level.^56^ Furthermore, these electrons can also interact with neighbouring Ti^4+^ giving Ti^3+^ centres and V_O_^+^ or V_O_^2+^. Ti^3+^ formed on the lattice surface could react with adsorbed H_2_O and O_2_ molecules giving rise to –OH groups and O_2_^−^ centres.^57,58^ The rearrangement of atomic positions within the lattice structure induced by V_O_ can ultimately result in the reduction in Ti–O bond length. This in turn, can create a strain on the TiO_6_ octahedra leading to the formation of Ti–O systems with different octahedral packing.^59^

##### 2.1.2.2 Formation of crystallographic shear planes and structural changes in TiO_2−*x*_

**Fig. 3:**
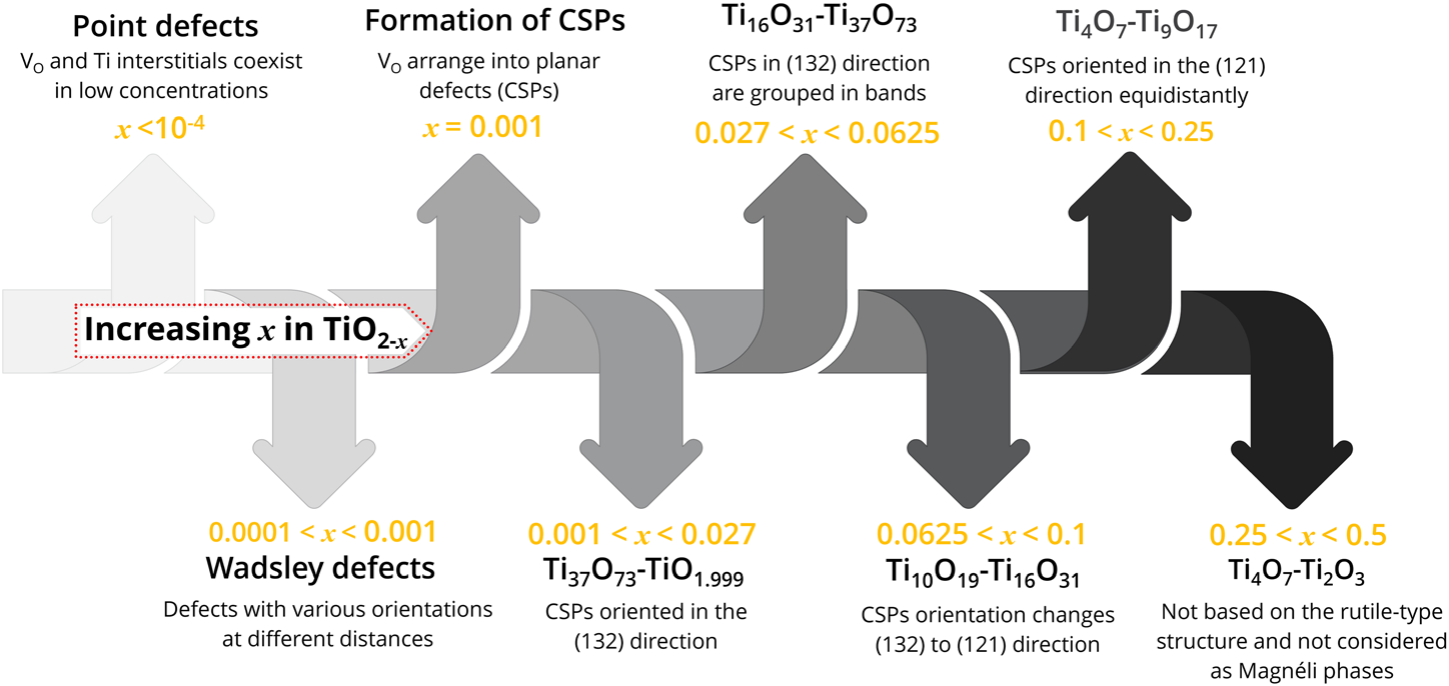
Formation of different defect induced TiO_2_ and phases with increasing *x* in TiO_2−*x*_.

Defective TiO_2_ (TiO_2−*x*_) and titanium suboxides are formed because of V_O_ and ionisation of these V_O_ which at the same time correspond to the reduction of Ti^4+^ to Ti^3+^.^60^ Depending on the value of *x* in TiO_2−*x*_, the types of defects present in TiO_2−*x*_ varies, along with their crystallographic structure, see Fig. 3. Slightly reduced TiO_2_ (TiO_2−*x*_, *x* < 10^−4^) is considered to only have point defects distributed in the matrix, where V_O_ and Ti interstitials coexist in low concentrations.^61^ When the *x* value in TiO_2−*x*_ falls between 0.001 and 0.0001, these are known as Wadsley defects. The increasing number of defects increases defect interaction and leads to defect ordering and structural transformation. Thereby, new compounds with different crystallographic structures are formed.

When the nonstoichiometry *x* in TiO_2−*x*_ reaches a critical value of *x* = 10^−3^ it causes V_O_ to arrange into planar defects known as CSPs.^31,60^ Formation of this shear structure can be considered as a way of eliminating point defects, forming regularly arranged extended planar defects.^60^ At CSPs, lattice planes move relative to each other, causing a collapse in the lattice structure. Such CSPs are common for d^0^ oxides including Ti, V, Mo and W.^17^ The structure of titanium suboxides with CSPs is described as a chain of rutile-like TiO_6_ octahedra interrupted every *n*th octahedron by a shear plane, where the octahedra share faces at the shear plane instead of usual edges and corners within the structure.^32^

When the *x* value in TiO_2−*x*_ falls between 0.027 and 0.001, new Ti_37_O_73_–TiO_1.999_ structures are formed. In these structures, the CSPs are oriented in the (132) direction.^31,60^ When *x* reaches 0.0625–0.027, Magnéli phases from Ti_16_O_31_–Ti_37_O_73_ are formed. In these structures, the shear planes oriented in (132) are grouped in bands.^62^ When *x* in TiO_2−*x*_ = 0.1–0.0625, higher Magnéli phases from Ti_10_O_19_ to Ti_16_O_31_ are formed. In these crystal structures, the shear plane orientation continuously changes from (132) direction to (121). When the *x* in TiO_2−*x*_ falls between 0.25 and 0.1 (for Ti_4_O_7_–Ti_9_O_17_), CSPs are oriented in the (121) plane equidistantly, forming ordered arrays of V_O_.

##### 2.1.2.3 Magnéli phases (Ti_*n*_O_2*n*−1_, 4 ≤ *n* ≤ 37)

**Fig. 4:**
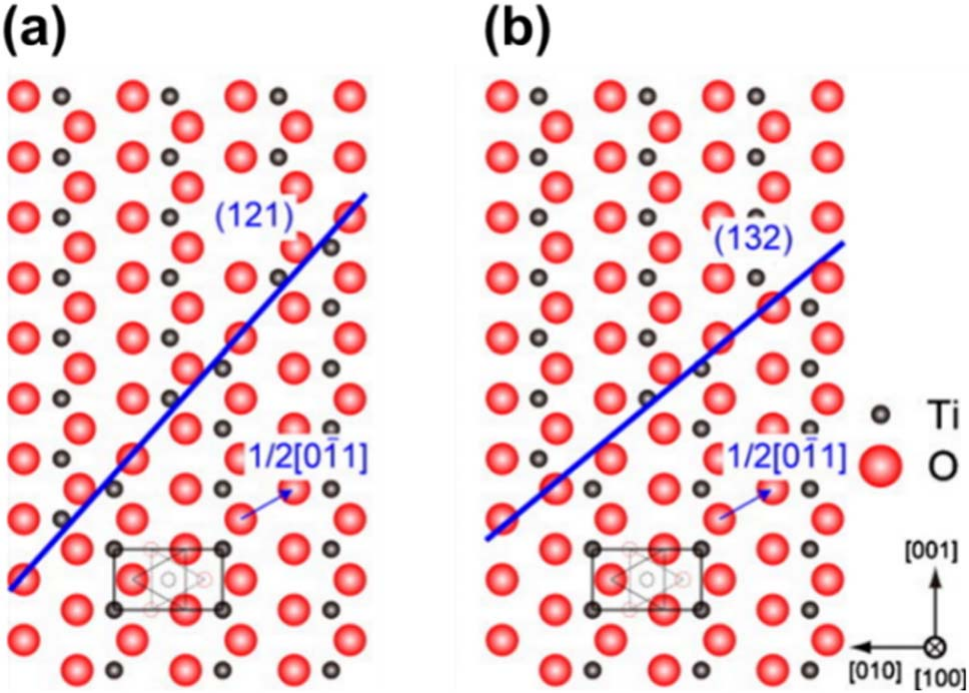
Crystallographic shear structure of Magnéli phase titanium suboxides showing the shear plane formation along the (a) (121) plane and (b) (132) plane. Reprinted with permission from ref. 63. Copyright 2010, AIP Publishing.

Titanium suboxides with CSPs in their structure are known as Magnéli phases and can be denoted by the formula Ti_*n*_O_2*n*−1_, where 4 ≤ *n* ≤ 37.^32^ Based on the concentration of CSPs (value of *n*), these Magnéli phases can be classified into two groups: in the series of suboxides with 10 ≤ *n* ≤ 37 (higher Magnéli phases), the CSP is introduced in the (132) plane in the structure of rutile TiO_2_, whereas the direction of the CSP changes to the (121) plane when 4 ≤ *n* ≤ 9 (Fig. 4). In all Magnéli phase suboxides, across the CSPs, rutile slabs are displaced relative to each other by the 
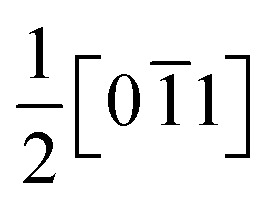
 vector, also known as the displacement vector.^63^ Such displacement requires the removal of an oxygen plane, thereby making the CSP oxygen deficient in comparison with rutile TiO_2_.^16^ Considering the nonstoichiometry of these Magnéli phases, the *y* value of TiO_*y*_ in higher Magnéli phases can range from 1.933 ≤ *y* ≤ 1.972 whereas for the lower Magnéli phases it ranges from 1.750 ≤ *y* ≤ 1.889.^64^

##### 2.1.2.4 Lower titanium suboxides

In the octahedral unit cell of Ti_3_O_5_ (space group *C*2/*m*), Ti atoms are surrounded by O atoms and are located at three distinct sites in the crystal, two Ti^3+^ and one Ti^4+^ sites.^65^ The presence of two types of metallic ions within the unit cell causes a distorted octahedron due to different Ti–O distances.^66^ Ti_2_O_3_ is reported to have the corundum (α-Al_2_O_3_) structure, with its stoichiometry extending from TiO_1.49_ to TiO_1.51_.^18,66^ Its fundamental unit cell comprises of pairs of Ti^3+^ ions situated in octahedral sites. TiO possesses a defective NaCl crystal structure (space group *Fm*3̄*m*), wherein there are an equivalent number of vacancies distributed randomly on both the cation and anion sites, with its homogeneity ranging from TiO_0.64_ to TiO_1.26_.^14,18^

#### 2.1.3 Structure of Magnéli phase titanium suboxides

The crystal structure of TiO_2_ typically contains point defects known as Wadsley defects. However, the formation of additional V_O_, as described earlier, results in the reorganisation of the crystal structure into new crystallographic phases and configurations.^67^ As mentioned in the previous section, when the *n* value in Ti_*n*_O_2*n*−1_ falls between 10 and 16, the orientation of the CSP changes from (132) to the (121) plane. Pure (121) plane CSP first appears for the Ti_*n*_O_2*n*−1_ Magnéli phase series when *n* = 9 and continues in the same plane for all suboxides where 4 ≤ *n* ≤ 9.^68^ Padilha *et al.* reports that the model for these oxygen deficient phases can be derived from rutile phase through a shear operation that involves the successive displacement of atoms within the rutile crystal. Specifically, all atoms located above each (121) plane are shifted *n* times along the *c* vector from the origin and are subsequently dislocated in the [01̄1] direction of the rutile structure. This direction aligns with a lattice vector of the oxygen subnet—namely, the vector [01̄1] that connects two oxygen atoms in the rutile crystal. This allows mapping of the dislocated atoms of that species onto atoms of the same species, while leaving the lattice positions of the oxygen atoms unchanged.^67^

**Fig. 5:**
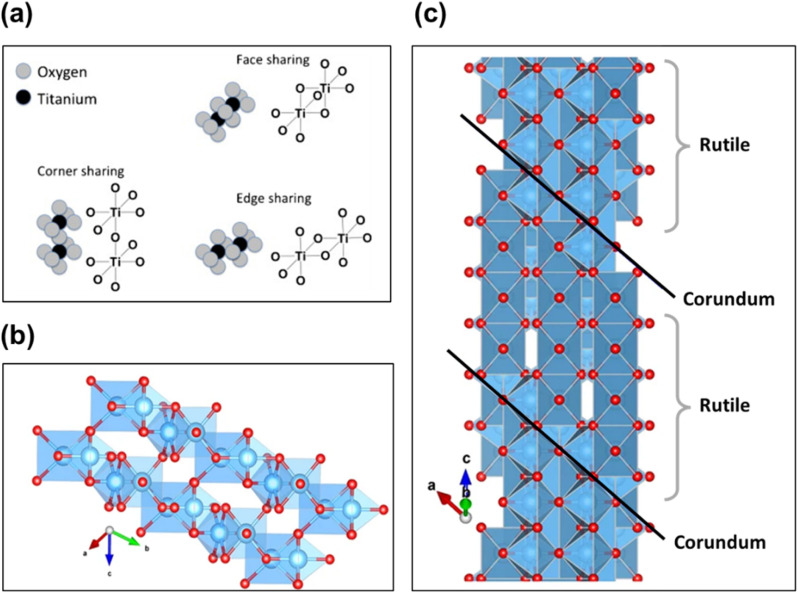
(a) Edge, corner and face sharing orientations of TiO_2_ octahedra in Magnéli phase titanium suboxides. Reproduced with permission from ref. 23. Copyright 2010, Elsevier. (b) Corundum Ti_2_O_3_ viewed parallel to the *c* axis and (c) structure of Ti_4_O_7_, showing four-unit rutile chains along the *c* direction bounded by corundum structures restricted in the (001) planes. Blue spheres represent the Ti atoms while the smaller red spheres represent O atoms.^67^

The rutile phase features an ordered arrangement of both oxygen and titanium atoms along the [001] direction. In contrast, the Magnéli phase maintains an ordered arrangement of oxygen atoms along the [001] direction but exhibits a titanium atomic mismatch along the (121) plane.^69^ The lattice structure of lower Magnéli phases can therefore be understood as a repeated pattern (block) of edge- and corner-sharing TiO_6_ rutile octahedra (as illustrated in Fig. 5(a)) that extend in two directions. However, in the third direction these blocks are disrupted by a plane of V_O_ in the (121) direction at every *n*th octahedra.^15,16,69^ This plane is formed of face sharing TiO_6_ octahedra, resembling a corundum (Ti_2_O_3_) structure (Fig. 5(b)). The boundaries created by the corundum structures in Magnéli phases are known as shear planes as illustrated in Fig. 5(c).^67^ This layer of face sharing octahedra serves both as the last layer of one block and the first layer of the adjacent one. This causes titanium atoms of one block to relate to the unoccupied or interstitial positions of the adjacent block. As a result, the symmetry of the structure changes from tetragonal to triclinic as the size of the unit cell increases.^21^ Parallel rutile blocks containing *n* number of octahedra (referred to as pseudorutile chains) link together through the terminal face sharing octahedron,^41,67,70^ leading to electronic interactions between the corresponding titanium atoms.^41,71^ Hence, in Magnéli phase, there is an ordered arrangement of oxygen atoms along the [001] direction, while a titanium atomic mismatch occurs on the (121) plane.^69^

Based on crystal structures previously reported for Magnéli phase titanium suboxides,^70,72,73^ Bowden *et al.* calculated X-ray powder diffraction patterns and compared them with experimental diffraction patterns.^74^ Calculated patterns for *n* = 4, 5 have shown good representation of the reported crystal data, though experimental patterns for *n* > 5 patterns were not presented due to the difficulty in obtaining their single phases.^74^ Crystal structure parameters for these Magnéli phase suboxides are given in Table 1.^74^

**Table 1:** Crystal structure parameters of Magnéli phase (Ti_*n*_O_2*n*−1_) titanium suboxides (4 ≤ *n* ≤ 9)

	Space group	*a* (Å)	*b* (Å)	*c* (Å)	*α* (Å)	*β* (Å)	*γ* (Å)	Reference
Ti_4_O_7_	*A*1̄	5.593	7.125	12.456	95.02	95.21	108.87	72
Ti_5_O_9_	*P*1̄	5.569	7.120	8.865	97.55	112.34	108.50	73
Ti_6_O_11_	*I*1̄	5.552	7.126	32.233	66.94	57.08	108.51	75
Ti_7_O_13_	*I*1̄	5.537	7.132	38.151	66.70	57.12	108.50	75
Ti_8_O_15_	*I*1̄	5.526	7.133	44.059	66.54	57.17	108.51	75
Ti_9_O_17_	*I*1̄	5.524	7.142	50.03	66.41	57.20	108.53	75

### 2.2 Physical properties of Magnéli phase titanium suboxides

#### 2.2.1 Electrical conductivity

TiO_2_ is recognised as an n-type semiconductor, and its conductivity is primarily attributed to donor-type defects, such as V_O_ and titanium interstitials.^76^ These defects contribute to the nonstoichiometry of TiO_2_, leading to an apparent deficiency of oxygen in the form of TiO_2−*x*_. Hence, the electrical conductivity (*σ*) of such an n-type semiconductor can be related to the electronic charge (*e*), carrier concentration (*n*) and carrier mobility (*μ*) as given in eqn (2):^77,78^

2 *σ* = *enμ*

**Fig. 6:**
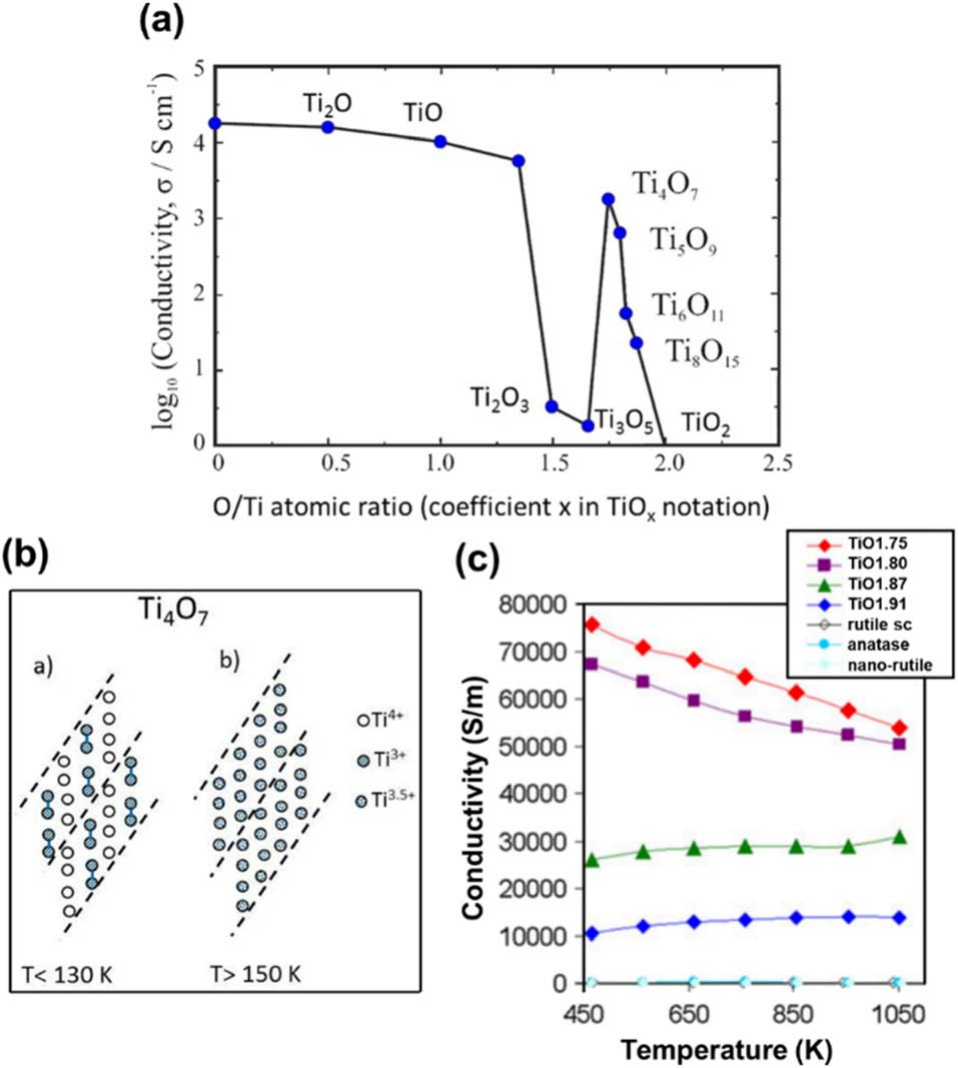
(a) Phase diagram of Magnéli phase titanium suboxides showing the variation of log of conductivity with oxygen stoichiometry. (b) Schematic representation of Ti_4_O_7_, showing the presence of Ti^3+^, Ti^3.5+^ and Ti^4+^ arranged in chains, interrupted by CSPs. At low temperature, Ti_4_O_7_ is non-metallic and clearly distinguishable Ti^3+^ and Ti^4+^ oxidation states are present. Reprinted with permission from ref. 32. Copyright 2022, Taylor & Francis. (c) Electrical conductivity as a function of temperature for rutile single crystals (sc), nano-rutile, anatase and different Magnéli phases. Reprinted with permission from ref. 86. Copyright 2012, Springer Nature.

Excellent electrical conductivity demonstrated by Magnéli phase titanium suboxides is attributed to the presence of shear planes and Ti^3+^, which serve as platforms for electron delocalisation.^79^ Formation of CSPs increases the carrier density and reduces the carrier mobility. This makes the electron mobility hopping-driven, where the electron hops between cation sites.^71^ The change in conductivity from electron conduction in Ti to a hopping driven mechanism in Magnéli phase titanium suboxides is attributed to the presence of oxygen and to the presence of both Ti^3+^ and Ti^4+^ in its structure.^15,80^ With the reduction in oxygen, Ti atoms in the lattice interact electronically leading to higher conductivity. As shown in Fig. 6(a), variation in electron conductivity in different *n* Magnéli phases is related to the difference in oxygen vacancy content.^23,81,82^ Ti oxides in the Ti_*n*_O_2*n*−1_ homologous series are mixed valence compounds, with two Ti^3+^ and (*n*-2) Ti^4+^ ions per formula unit. Furthermore, based on electron paramagnetic resonance spectra studied between 90 and 150 K in Magnéli phase single crystals, Fairhurst and co-workers reported that the intrinsic components in the crystal structure of Ti_*n*_O_2*n*−1_ (*n* = 5–8) are Ti_2_^7+^ oxygen-bridged dimers.^41,59,83^

Magnéli phase titanium suboxides have highest conductivity for lowest *n* values (*n* = 4, 5) and exhibit phase transitions with temperature. Such transitions have been attributed to the presence of both Ti^3+^ and Ti^4+^, which provide possibilities for electron localisation at these cation sites, leading to different charge ordered states.^84^ Ti_4_O_7_, the most conductive Magnéli phase titanium suboxide, is reported to undergo two phase transitions with temperature. At 130 K it undergoes a semiconductor–semiconductor transition and a semiconductor–metal transition at 150 K.^15,85^ Similar observations had previously been made by Bartholomew *et al.* in their electrical property measurement of some titanium oxide Magnéli phases. They reported that Ti_4_O_7_ showed metallic behaviour at room temperature and undergoes a phase transition at 149 ± 2 K to a semiconducting phase, with a reduction in conductivity, followed by a significant decrease in magnetic susceptibility. A second transition has been observed at 125 K with three-orders of magnitude decrease in conductivity, but with no change in susceptibility.^21^ At low temperatures, the Ti^3+^ in Ti_4_O_7_ covalently bond with each other, forming bipolarons (Ti^3+^–Ti^3+^ pairs) and occupy alternate pseudorutile chains to form a low temperature (LT) semiconducting phase. A reordering of these Ti^3+^–Ti^3+^ pairs occurs during semiconductor–semiconductor transition from LT to intermediate temperature (IT). At high temperature (HT) all Ti cations are present as Ti^3.5+^ with no apparent pairing leading to highly conductive metallic behaviour (Fig. 6(b)).^72,85,87^ Watanabe *et al.* measured the Raman spectra of Ti_4_O_7_ as a function of temperature and observed a stable charge ordered state across the electronic phase transition temperatures.^88,89^ Magnetic susceptibility measurements conducted for the material showed a strong increase in paramagnetic susceptibility at 150 K where the semiconductor–metal transition takes place. However, at temperatures below 150 K, it is antiferromagnetic.^90,91^

Such phase transitions have also been observed in Ti_5_O_9_, at 128 K and 139 K. Although LT and IT phases are semiconducting, the conductivity of the HT phase increases with increasing temperature, making it difficult to be classified as a true metal.^15,21,84^ Electrical conductance measured for higher *n* Magnéli phases have shown that two transitions occur for Ti_6_O_11_ at 147 K and 119 K and one transition for Ti_7_O_11_ at 120 K separating regions showing non-metallic behaviour.^15,21^ However, conductance measurements carried out for Ti_8_O_15_ and Ti_9_O_17_ have not shown any clear steps between 4 K < *T* < 295 K. But a discontinuous change in the first derivative has been observed at 120 K, which was more evidently observed in Ti_7_O_13_.^15^ Bartholomew *et al.* reported that higher titanium suboxides (Ti_8_O_15_) were found to be semiconducting over the temperature range 78–295 K.^21^

Fan *et al.* compared the electrical conductivities of Ti_4_O_7_ and Ti_9_O_17_ samples prepared *via* hydrogen reduction. The study revealed that Ti_4_O_7_ has an electrical conductivity of 9.92 × 10^4^ S m^−1^ at room temperature (RT), which decreases as temperature rises. This metallic behaviour further confirmed that Ti_4_O_7_ becomes a highly paramagnetic metal following a semiconductor-to-metal transition above 150 K, due to the delocalisation of 3d electrons from Ti^3+^ ions. On the other hand, Ti_9_O_17_ exhibited typical semiconductor characteristics from RT to 817 K, with electrical conductivity nearly an order of magnitude lower than that of Ti_4_O_7_ at RT, reflecting a lower level of electron doping. Furthermore, the carrier concentration of Ti_4_O_7_ was an order of magnitude higher than that of Ti_9_O_17_, with values of 17.2 × 10^21^ cm^−3^ for Ti_4_O_7_ and 1.83 × 10^21^ cm^−3^ for Ti_9_O_17_. These findings suggested that CSPs do not act as sources of electron diffraction, since the concentration of CSPs is higher in Ti_4_O_7_ compared to Ti_9_O_17_.^77^ Backhaus-Ricoult and co-workers reported changes in electrical conductivity as a function of temperature for ceramic powders (TiO_2−*x*_, *x* = oxygen deficiency) containing mixtures of Magnéli phases. TiO_1.75_ was composed of *n* = 4–5 (in Ti_*n*_O_2*n*−1_) phases with Ti_2_O_3_, TiO_1.80_ with *n* = 4–6 phases and Ti_2_O_3_, TiO_1.87_ with *n* = 6–8 phases and TiO_1.91_ composed of other (possibly higher) Magnéli phases. Their observations showed that the electrical conductivity increased with increasing oxygen deficiency (Fig. 6(c)). Ceramics with lower deficiency demonstrated semiconducting behaviour with increasing conductivity with temperature while those with Ti_4_O_7_ and Ti_5_O_9_ phases showed metallic behaviour with decreasing conductivity with increasing temperature.^86^ Adamaki *et al.* measured the AC electrical properties of Magnéli phase titanium suboxides up to 375 °C and compared them with those obtained for TiO_2_. Low frequency (100 Hz) electrical conductivities of Magnéli phase titanium suboxide fibres reduced at 800–1100 °C showed increased conductivity between 10^−1^ and 10 S m^−1^ in contrast to the insulating behaviour of rutile TiO_2_. Samples reduced at 1200–1300 °C demonstrated metallic behaviour with 10^3^–10^4^ S m^−1^ conductivity at low frequency measurements. When the measurements were conducted at a wider range of frequencies (1–10^5^ Hz), fibres reduced at 1200 and 1300 °C behaved as conductors showing that their conductivity was frequency independent across the frequency range examined.^92^ The conductivity of titanium suboxides is reported to be significantly higher than that of TiO_2_, in a wider range of frequencies from 0.1 Hz to 1 kHz.^93^

#### 2.2.2 Thermal conductivity

Thermoelectric efficiency of a material is evaluated by a dimensionless figure of merit, *ZT*, and is related to three physical properties of the material, Seebeck coefficient (*α*), electrical resistivity (*ρ*) and thermal conductivity (*λ*). The dependence of *ZT* on these physical properties and absolute temperature (*T*) is given as follows (eqn (3)):3
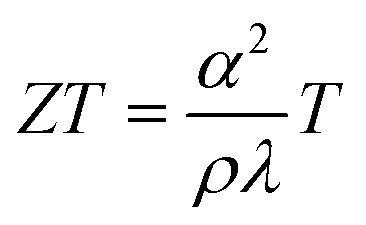


Thermal conductivity is a combination of two components: phonon (lattice) contribution (*κ*_l_) and carrier concentration (*κ*_e_). Lattice imperfections help in scattering of phonons with different wavelengths leading to a reduction in *κ*_l_. Point defects, grain boundaries, phase boundaries, interfaces and dislocations are some crystal imperfections that act as phonon scattering sources.^94^ Thereby, a reduction in lattice thermal conductivity is reported to exhibit enhanced thermoelectric performance in the material.^63,95^ The large number of V_O_ present in Magnéli phase titanium suboxides (in CSPs) serve as phonon scattering centres that can reduce the mean free path of phonons.^96^ This reduction reduces the lattice thermal conductivity, which in turn can affect the total thermal conductivity and thermopower.^97,98^The *κ*_e_ is estimated from electrical conductivity (*σ*) through the Wiedemann–Franz law, *κ*_e_ = *σLT*, where *L* is the Lorentz number and *T* is temperature. Although the Lorentz number is constant in metals, in semiconductors, it slightly depends on the carrier concentration.^99^ However, carrier concentration does not affect *κ*_l_, suggesting that materials with good thermoelectric performance demonstrate low lattice thermal conductivity.

**Fig. 7:**
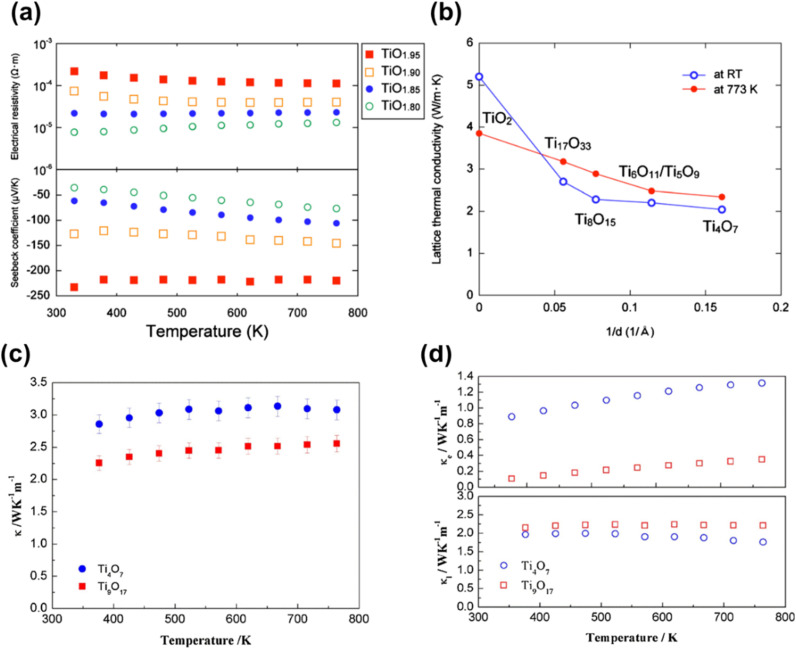
(a) Variation of electrical resistivity (top) and Seebeck coefficient (bottom) with temperature for hot pressed TiO_2−*x*_ specimens. (b) Variation of lattice thermal conductivity with the density of CSPs (reciprocals of the spacing of CSPs) at RT and 500 °C. Reprinted with permission from ref. 63. Copyright 2010, AIP publishing. (c) Variation of thermal conductivity (*κ*), (d) electron thermal conductivity (*κ*_e_) (top) and lattice thermal conductivity (*κ*_l_) with temperature for Ti_4_O_7_ and Ti_9_O_17_. Reprinted with permission from ref. 77. Copyright 2018, Elsevier.

Harada *et al.* measured the variation in thermoelectric properties of a series of hot pressed Magnéli phase TiO_2−*x*_ powders with change in the *x* value (Fig. 7(a)). From the results obtained for TiO_2−*x*_ powders (*x* = 0.05, 0.10, 0.15 and 0.20), it was noted that the value of electrical resistivity and thermal conductivity decrease when the value of *x* increases, and the absolute value of the Seebeck coefficient decreases with an increase in *x*. However, the value of lattice thermal conductivity decreased with increasing oxygen deficiency (*x*) (Fig. 7(b)). Based on the dependence of electrical resistivity and lattice thermal conductivity on oxygen deficiency, it was concluded that CSPs act as sources for phonon scattering but not carrier scattering.^63^

Reduced rutile TiO_2_ and highly deficient Magnéli phase TiO_2−*x*_ powders demonstrated a decrease in thermal conductivity with decreasing O/Ti ratio (*i.e.*, increasing *x*). Furthermore, it was also determined that phonon scattering was mostly affected by planar defects compared to point defects and hence planar defects play a critical role in the thermal conductivity of TiO_2−*x*_ materials.^69^ Thermoelectric studies conducted by Fan *et al.* showed that the thermal conductivity of Ti_9_O_17_ was lower than that of Ti_4_O_7_, demonstrating better overall thermoelectric performance compared to the latter (Fig. 7(c) and (d)).^77^ In measurement of thermoelectric properties of plasma spray-synthesised TiO_2−*x*_ deposits, Lee *et al.* found the introduction of V_O_ into substoichiometric titanium oxides with the resultant formation of Ti_4_O_7_ increases electrical conductivity but reduces the Seebeck coefficient. It was further observed that the presence of a porous, defective structure with interfaces in these deposits caused a significant reduction in through-thickness thermal conductivities.^100^

#### 2.2.3 Optical properties

The optical properties of a material, such as its refractive index and absorption coefficient, can be determined by examining the spectral dependence of transmittance and reflectance. The spectral dependence of the absorption coefficient can be used to determine the bandgap energy, which represents the energy of the fundamental optical transitions from the valence band (VB) to the conduction band (CB).^19^

**Fig. 8:**
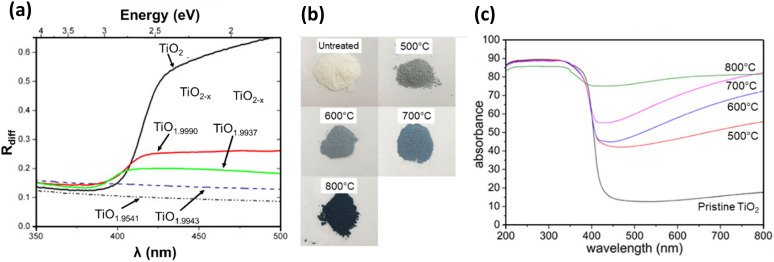
(a) Diffuse reflectance spectra of TiO_2_ and reduced TiO_2−*x*_ showing the variation in light absorbance with changes in oxygen stoichiometry. Reprinted with permission from ref. 19. Copyright 2007, Elsevier. (b) Digital images of untreated and reduced rutile TiO_2_ and (c) UV-vis absorption (percentage scale) spectra of untreated and reduced TiO_2_ hydrogenated at 500–800 °C. Reprinted with permission from ref. 105. Copyright 2019, American Chemical Society.

As shown in Fig. 8(a), stoichiometric TiO_2_ shows a dramatic rise in reflectance, in the range 390–420 nm. The change in the optical reflectance that occurs in this range is associated with the beginning of the tail of the absorption curve of TiO_2_. The optical bandgap of the material is determined from the wavelength of the inflection point of the absorption curve (edge). Due to the surface reflectivity of TiO_2_, the shorter wavelength region (*λ* < 400 nm) of its reflectance curve consists of a region of constant reflectivity. This is because if the particles are all absorbing, the absorption coefficient has minimal impact until it approaches the centre of the main absorption band. However, the particles become relatively transparent with reducing absorption coefficient at longer wavelengths (*λ* > 450 nm). This in turn increases the net diffuse reflectivity of the sample, due to numerous reflections and refractions occurring in the bulk of the material. Hence, when the value of the absorption coefficient is very low, diffuse reflectivity reaches 1.^19^

As explained in the previous paragraph, rutile based TiO_2_ samples show the most significant variation in reflectance between 390 and 420 nm related to their fundamental absorption edge. Reflectance on the shorter wavelength side of this is independent of the oxygen stoichiometry in TiO_2_, while on the long wavelength side, reflectance decreases with increasing oxygen non stoichiometry.^19,101^ Based on the ionisation state of V_O_ in rutile, they can act as single or double electron donors. Hence, the decrease in reflectance with increasing V_O_ is related to sample conductivity, which increases when the number of free electrons in the lattice increases.^101^ Furthermore, studies have also reported a less intense broad band at 500–600 nm, caused by reduction. This band is attributed to the presence of two-electron centred V_O_.^102^

The linear region of the slope in the reflectivity *vs.* wavelength plot (Fig. 8(a)) was absent for highly reduced nonstoichiometric samples. This is because the additional states created during reduction absorb light related to photon energies within the forbidden bandgap.^19^ Because of this, all Magnéli phases are known to be highly active under visible light, extending their absorbance from visible to the near IR region.^29,103,104^

Liu *et al.* made similar observations in the optical properties of hydrogen reduced rutile TiO_2_ annealed at different temperatures (Fig. 8(b)). The absorbance of UV light in the *λ* < 400 nm region was similar for both pristine and hydrogen reduced TiO_2_. This similarity implies that the presence of V_O_ on the surface of the particles has minimal impact on UV absorption. However, increasing reduction temperature resulted in higher absorption in the longer wavelength region (*λ* > 400 nm), attributed to the creation of oxygen defects that form mid gap states between the VB and CB. The sample reduced at the highest temperature (800 °C), comprising a mixture of Ti_9_O_17_ with rutile TiO_2_, showed the highest visible light absorbance, owing to the presence of the highest amount of oxygen defects (Fig. 8(c)).^105^

#### 2.2.4 Electronic structure and properties

**Fig. 9:**
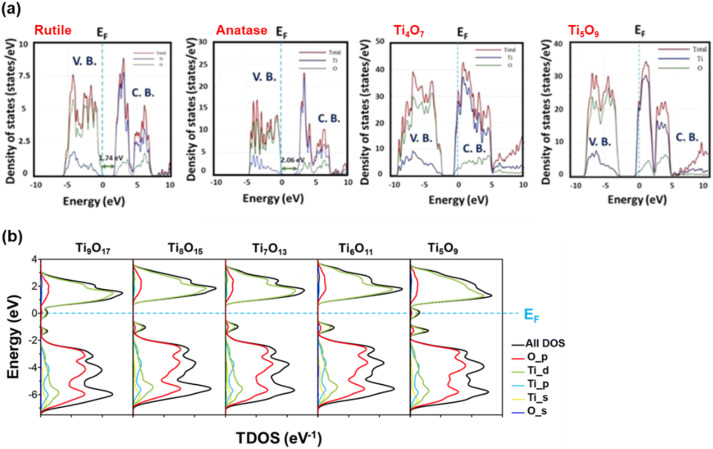
(a) Normalised DOS diagram for rutile, anatase, Ti_4_O_7_ and Ti_5_O_9_ (ref. 106) and (b) Calculated total DOS of Magnéli phase Ti_*n*_O_2*n*−1_ (5 ≤ *n* ≤ 9).^30^

In the normalised electron density of states (DOS) for both rutile and anatase, as reported by Malik *et al.*, CB is predominantly composed of Ti 3d states, while the VB is comprised of O 2p electrons (Fig. 9(a)).^106^ Similar observations were made by Niu and coworkers in the total DOS calculated for anatase TiO_2_, and the Fermi level is located just above the VB maximum, confirming its semiconducting nature.^107^ But in Magnéli phase Ti_*n*_O_2*n*−1_ (*n* = 4–9), the Fermi level shifts into the CB, indicating the presence of free electrons, thus imparting metallic characteristics and excellent electrical conductivity.^107^ When studying the electronic structure of Ti_4_O_7_ and Ti_5_O_9_, Malik *et al.* also showed that for these two suboxides, V_O_ introduce an intermediate band formed by pseudo-point defects. This intermediate band emerges under reduced atmospheric conditions when Ti^4+^ interstitials in the CB are converted into Ti^3+^, which act as shallow donor states below the CB minimum. As shown in Fig. 9(a), for both Ti_4_O_7_ and Ti_5_O_9_, the intermediate band formed by Ti^3+^ pseudo-defects lowers the energy of the CB below the Fermi level, confirming the semi-metallic states of these Magnéli phases, where electron behaviour resembles that of metals.^106^

Padilha *et al.* demonstrated that DFT-based calculations could identify defect levels within the bandgap region for all Ti_*n*_O_2*n*−1_ phases (where 2 < *n* < 5). These defects, similar to those in rutile TiO_2_, primarily involve Ti 3d orbitals and arise due to the presence of intrinsic V_O_. Improved descriptions of Ti 3d orbitals were obtained using either the Hubbard U parameter or hybrid functionals, which shifted these defect levels away from the CB minimum, depicting them as either shallow or deep levels.^67^ Slipukhina *et al.* conducted first-principles calculations on Ti_5_O_9_ to investigate its electronic and magnetic properties. They identified several quasi-degenerate magnetic solutions for all phases—low, intermediate and high temperatures—making it challenging to determine the precise charge distribution at various temperatures. They noted that the charge and orbital orders across all three phases were non-unique, and the formation of Ti^3+^–Ti^3+^ bipolaronic states was less likely than in Ti_4_O_7_, though not impossible. Additionally, they concluded that phase transitions in Ti_5_O_9_ result from complex interrelations between electronic correlations, electron–lattice and spin–lattice coupling, rather than structural changes alone.^84^

Ekanayake *et al.* used DFT simulations to explore the electronic and optical properties of Magnéli phase Ti_*n*_O_2*n*−1_ (*n* = 5–9). Their total DOS analysis (Fig. 9(b)) confirmed that the CB minimum is largely composed of Ti d orbitals, while the VB consists of O 2p orbitals. Their findings also indicated strong d–d correlation effects in these titanium suboxides, which led to the emergence of new bands within the Ti–O gap, thus reducing the bandgaps from the 3.2 eV observed in TiO_2_. Spin–orbit coupling introduced additional bands derived from Ti 3d and O 2p orbitals, especially in Ti_9_O_17_ and Ti_5_O_9_, creating high-energy mid-gap states near the Fermi level. This in turn led to further narrowing of the bandgaps of these phases contributing to metallic behaviour.^30^

## 3. Methods to synthesise Magnéli phase titanium suboxides

**Fig. 10:**
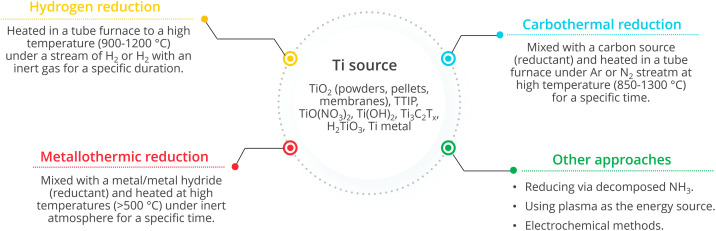
Different synthesis approaches for Magnéli phase titanium suboxides (TTIP is titanium(iv) isopropoxide and T_*x*_ indicates functional groups) and a typical process for each method.

Synthesis approaches used to prepare Magnéli phase titanium oxides can be divided into two groups: partial reduction from TiO_2_ and incomplete oxidation from low valence titania species.^54^ Reduction approaches include hydrogen reduction, argon annealing, metal reduction, reduction with organic reductants, high energy particle reduction and electrochemical reduction. Oxidation approaches involve the use of raw Ti materials including metallic titanium, organotitanium and inorganotitanium to generate TiO_2−*x*_ in an oxygen containing environment.^33^Fig. 10 summarises the fate of different Ti sources and the typical strategies employed in each of the synthesis methods.

### 3.1 Hydrogen reduction

One of the most commonly used approaches to form Magnéli phase titanium suboxides is hydrogen reduction, which makes use of either pure H_2_ gas under low or high pressure, mixtures of H_2_/Ar or H_2_/N_2_ or the use of hydrides such as NaBH_4_ or CaH_2_.^108–112^ The reduction reaction occurring in the presence of hydrogen can be written as shown in eqn (4).^33^

4 *n*TiO_2_(s) + H_2_(g) → Ti_*n*_O_2*n*−1_(s) + H_2_O(g)

In a typical synthesis, the TiO_2_ source (such as amorphous or crystalline powders, pellets or membranes) is first placed inside the reactor, typically a tube furnace. The furnace is then heated to a high temperature, usually between 900 and 1200 °C, under a stream of pure H_2_ or a mixture of H_2_ and an inert gas.

The reaction is maintained for a specific duration (typically 2–8 h), after which the resulting product—whether in powder, pellet, or membrane form—is collected for further testing and characterisation. When different precursors, such as hydroxides, alkoxides, nitrates or MXenes, are used as the Ti source, they are first converted into TiO_2_ through methods like hydrolysis or hydrothermal treatment. In some cases, these precursor forms can be directly subjected to a hydrogen stream to form the desired Magnéli phases.

Table 2 summarises various studies that have made use of hydrogen reduction to prepare Magnéli phase titanium suboxides using different titanium precursors.

**Table 2:** Magnéli phase titanium suboxides synthesised *via* hydrogen reduction using different starting materials and reduction conditions

Ti precursor	Synthesis/treatment of the precursor	Reduction conditions	Product composition	Ref.
Amorphous TiO_*x*_	Addition of H_2_O_2_ to a mixture of TiCl_4_ and ethylenediaminetetraacetic acid caused the decomposition of the Ti complex into colloidal TiO_*x*_. The resulting mixture after precipitating and filtering yielded amorphous TiO_*x*_	5 g of amorphous TiO_*x*_ was heated in a quartz tubular reactor at 1050 °C for 2 h with a heating rate of 5 °C min^−1^ under 10% H_2_ (Ar) stream fed at 20 cm^3^ min^−1^	Mixture of Ti_9_O_17_ and Ti_6_O_11_	113
Anatase TiO_2_	—	Reducing anatase TiO_2_ powder at 950 °C for 4 h under pure H_2_ flow of 200 mL min^−1^. Ti_4_O_7_ pellet formed by reducing TiO_2_ pellet at 1050 °C for 2 h	Ti_4_O_7_ powder/Ti_4_O_7_ pellet	114
	TiO_2_ pellets were initially formed by uniaxial pressing followed by sintering at 1327 °C for 5 h in air	Reduction of TiO_2_ pellets was conducted in a 7% H_2_ (Ar) atmosphere for 3.5 h from 947 to 1147 °C	Combinations of TiO_2_ and Ti_4_O_7_ obtained at 997 °C and 1067 °C and a mixture of Ti_9_O_17_ and Ti_8_O_15_ obtained at 1147 °C	19
	TiO_2_ was mixed with MilliQ water, triethanolamine and polyethylene oxide solution to form a suspension. This mixture was dip-coated on a tubular α-Al_2_O_3_ support, dried at RT and at 100 °C followed by sintering in air at 800 °C	Reduced in a tube furnace from 850 to 1050 °C (5 °C min^−1^ heating rate) under 30% H_2_ (N_2_) atmosphere for 2–7 h	Ti_4_O_7_/Al_2_O_3_ membrane	115
Anatase TiO_2_ nanosheets	Electrostatic deposition of TiO_2_ nanosheets on SiO_2_ beads	Annealing at 950 °C for 7 h under 200 mL min^−1^ H_2_ flow	SiO_2_@Ti_4_O_7_	116
Rutile TiO_2_	Rutile TiO_2_ powders mixed with water : isopropanol (1 : 1, v/v) and 5 wt% (w/w) polyethylene oxide binder and compressed at 20 MPa to form a plate	Annealing at 1050 °C for 4 h under H_2_ atmosphere	Monolithic porous Ti_4_O_7_	22
	Rutile TiO_2_ mixed with different weight percentages of PVA	Annealed at 1100 °C for 1 h at a heating rate of 3 °C min^−1^ under 5% H_2_ (Ar) atmosphere	A mixture of Ti_4_O_7_ and Ti_5_O_9_ was obtained when 75 wt% PVA and 25 wt% TiO_2_ was used	117
	—	Reduced at 800 °C heated at a rate of 10 °C min^−1^ under pure H_2_ gas at 1.0 L min^−1^	Mixture of rutile TiO_2_ and Ti_9_O_17_	105
TiO_2_ (Degussa, P25)	P25 was oxidised (800 °C, 2 h) to remove any organic contaminants	Reduced in a 4% H_2_ (Ar) atmosphere at 1000 °C, 1100 °C and 1180 °C for 2 h at a heating rate of 1 °C min^−1^	A mixture of Ti_9_O_17_/Ti_6_O_11_ was obtained at 1000 °C and Ti_4_O_7_/Ti_5_O_9_ at 1100 °C	118
	Co ions deposited on TiO_2_ powders obtained by dispersion of CoCl_6_·6H_2_O in TiO_2_ powder in ethanol followed by stirring at RT, solvent evaporation at 65 °C and drying at 50 °C for 12 h	Annealed under H_2_ atmosphere with a flow rate of 100 mL min^−1^ and heated at 400–800 °C for 3 h	Ti_8_O_15_	119
Commercial pigment TiO_2_	Mixtures of TiO_2_/polyethylene glycol solution compacted in a uniaxial press followed by sintering in air at 1050 °C for 1 day	Reduced at 1050 °C for 4 h	Monophasic Ti_4_O_7_ electrodes	120
TiO_2_ powder	—	Reduced at 1050 °C for 6 h under H_2_ flow in a tube furnace	Ti_4_O_7_/Ti_6_O_11_	121 and 122
	Reduced in a tube furnace at 1050 °C for 5 h in the presence of 1.0 atm H_2_ flow	0.5 g of the formed Ti_4_O_7_ powder was mixed with the binder, paraffin oil and pelletised using a hydraulic press and further reduced at 1050 °C for 6 h under H_2_ flow to remove the binder	Ti_4_O_7_ electrode	123
TiO_2_ membrane	—	Reduced in a tube furnace at 1050 °C for 30 h in a H_2_ atmosphere	Ti_4_O_7_/Ti_6_O_11_ REM	124
TiO_2_ reactive electrochemical membrane (REM)	REMs were synthesised in a tubular TiO_2_ ultrafiltration membrane (50 kDa), followed by cutting the membrane to 10 cm in length	REMs reduced in a tube furnace at 1050 °C under 1 atm H_2_ flow for 30–50 h	Ti_6_O_11_ membrane when treated for 30 h, mixture of Ti_6_O_11_ and Ti_4_O_7_ at 40 h and Ti_4_O_7_ membrane at 50 h	26
Titanium(iv) isopropoxide (TTIP)	Decomposition of TTIP vapour inside a hydrogen stream thermal aerosol hot wall reactor formed TiO_2_ aerosol	TiO_2_ was reduced by the H_2_ stream between 500 and 1100 °C at 100 mbar	100% Ti_4_O_7_ at 1100 °C, 60% Ti_4_O_7_ and 21% Ti_5_O_9_ with 5.5% anatase TiO_2_ at 1000 °C and 24% Ti_4_O_7_, 19% Ti_5_O_9_ with 31.4% anatase TiO_2_ formed at 900 °C	125
	TTIP solution is converted to Ti(OH)_2_ in water, which is further converted to titanium nitrate using HNO_3_. A colloidal crystal template formed of silica nanoparticles was added into the above solution followed by drying at 120 °C and further at 200 °C for 3 h	Heated under H_2_ flow with a flow rate of 200 mL min^−1^ in a tube furnace at 800 °C for 5 h	Mesoporous Ti_6_O_11_ with trace amounts of anatase TiO_2_	126
TiO(NO_3_)_2_	Nitric acid added to a mixture of Ti(OC_4_H_9_)_4_)/distilled water so that Ti(OC_4_H_9_)_4_ would undergo hydrolysis and react with HNO_3_ to form TiO(NO_3_)_2_ as follows:^106^	TiO(NO_3_)_2_ was then annealed in a tube furnace at 1000 °C for 6 h at a 5 °C min^−1^ heating rate under 200 sccm H_2_ flow	Ti_4_O_7_ nanoparticles	127
	Ti(OC_4_H_9_)_4_ + 3H_2_O → TiO(OH)_2_ + 4C_4_H_9_OH			
	TiO(OH)_2_ + 2HNO_3_ → TiO(NO_3_)_2_ + 2H_2_O			
H_2_Ti_3_O_7_ nanorods	Anatase TiO_2_ nanoparticles hydrothermally treated with NaOH at 150–180 °C for 2–5 days to form H_2_Ti_3_O_7_	H_2_Ti_3_O_7_ nanorods are heated under a H_2_ atmosphere between 800 and 1050 °C for 1–4 h	Ti_8_O_15_ nanorods were obtained at 850 °C and Ti_4_O_7_ fibres at 1050 °C	103
Ti(OH)_2_ and anatase TiO_2_	Ti(OH)_2_ was precipitated from H_2_TiCl_6_ and was thoroughly washed followed by drying at 100 °C. To form anatase TiO_2_, Ti(OH)_2_ was hydrothermally treated at 200–300 °C for 3 h	Reduced in a Tamman furnace under H_2_ atmosphere (flow rate of 5 L min^−1^) at 800, 900 and 1000 °C for 1, 5.5 and 27.5 h	Reduction of Ti(OH)_2_ and anatase TiO_2_ at 1000 °C for 1 h: Ti_9_O_17_	128
			Reduction of anatase TiO_2_ at 1000 °C for 5.5 h: mixture of Ti_9_O_17_, Ti_8_O_15_ and Ti_5_O_9_	
MXenes (Ti_3_C_2_T_*x*_)	Peroxo titanic acid gel, formed by a mixture of Ti_3_C_2_T_*x*_ and H_2_O_2_) was dropped on to an etched quartz glass followed by oven drying at 60 °C (where T_*x*_ indicates fucntional groups)	Annealed at 800–1000 °C under H_2_ atmosphere for 1 h	Magnéli phase flexible film (Ti_4_O_7_/Ti_5_O_9_ at 1000 °C and Ti_6_O_11_/Ti_7_O_13_ at 900 °C)	129

The types of Magnéli phases obtained from each method indicate that the key factor in hydrogen reduction is the temperature at which these phases are generated. Generally, higher temperatures and/or longer heating times result in Ti_4_O_7_ and other lower-*n* Ti_*n*_O_2*n*−1_ phases, whereas mixed phases tend to appear at lower temperatures or with shorter heating times. Additionally, some studies have explored the mixing of different Ti sources, solvents and carbon sources before H_2_ reduction. For example, Krishnan *et al.* examined the influence of poly(vinyl alcohol) (PVA) on Magnéli phase formation. They investigated mixtures of TiO_2_ and PVA (25–75% PVA by weight) and subsequently annealed the samples under a hydrogen atmosphere. PVA was utilised to reduce the agglomeration of Ti_*n*_O_2*n*−1_ and enhance the surface area. Their findings showed that the highest reduction in agglomeration and the largest surface area (25.3 m^2^ g^−1^) were achieved with 75 wt% PVA.^130^ You *et al.* mixed TiO_2_ powder with water and isopropanol (1 : 1, v/v) before hydrogen treatment to reduce the capillary force within the powder. The resulting sample consisted of Ti_4_O_7_; however, their study did not explore the effect of varying the water-to-isopropanol ratio on the formation of Magnéli phases.^131^ Furthermore, while many studies employed a specific H_2_ stream flow rate, none have discussed its impact on particle size or the types of Magnéli phases formed. Therefore, the synthesis temperature, as well as the use of dispersants and solvents, are crucial design considerations for H_2_-based reduction methods to achieve the desired phase and particle size.

As shown in Table 2, hydrogen reduction is a popular method for synthesising Magnéli phases due to its ability to operate at lower temperatures (as low as 800 °C)^126^ and shorter reduction times (such as 1 h)^129,130^, thereby saving both time and energy. This method also offers the potential to obtain Magnéli phase titanium suboxide nanoparticles, depending on the precursor used. Despite these advantages, hydrogen reduction has significant drawbacks, primarily due to the high risk of explosion associated with working with hydrogen, as well as the higher costs involved in the H_2_ storage and transportation.^132^ As a result, alternative methods have been developed for synthesising Magnéli phase titanium suboxides.

### 3.2 Carbothermal reduction

Reduction of TiO_2_ using carbon or an organic polymer under an inert atmosphere is termed carbothermal reduction and occurs according to the following reaction (eqn (5)).^33^

5 *n*TiO_2_(s) + C(s) → Ti_*n*_O_2*n*−1_(s) + CO(g)

Carbothermal reduction has been widely used in recent studies owing to the safety and low cost of the method compared to other approaches. Furthermore, contact between the reactant and the reductant has shown an increased reaction rate compared to gas–solid reactions.^133^ It is reported that during carbothermal reduction, V_O_ in the TiO_2_ lattice are initially increased followed by lattice reconstruction in the final stage.^133^ The ratio between TiO_2_ : carbon plays a critical role in the phase of the final product, since an over stoichiometric ratio can lead to the formation of TiC or TiO_*x*_C_*y*_.^134–136^ Furthermore, the kinetics of the TiO_2_ carbothermal reduction depends heavily on the reaction temperature, initial bulk density, gas atmosphere and TiO_2_ grain size.^134,137^

In a typical synthesis, TiO_2_, in the form of powder or pellets, is mixed with a carbon-based reductant such as carbon black, activated charcoal, or a polymer like PVA or polyethylene glycol (PEG). The mixing of the TiO_2_ and the carbon source is usually a physical process involving techniques such as ball milling, ultrasonication or manual mixing. The resulting mixtures are then heated in a tube furnace under an inert atmosphere at temperatures ranging from 850 to 1300 °C for 1 to 24 h, producing a series of products with various combinations of Magnéli phases. Table 3 summarises some research conducted on synthesising Magnéli phase titanium suboxides using the carbothermal reduction. The overall trend indicated that increasing the treatment/reduction time resulted in a higher proportion of lower Magnéli phases being formed.

**Table 3:** Magnéli phase titanium suboxides synthesised *via* carbothermal reduction using different starting materials and reduction conditions

Ti precursor	Synthesis/treatment of the precursor	Reduction conditions	Product composition	Ref.
Anatase TiO_2_	Anatase TiO_2_ mixed with carbon black was ball milled for 24 h at 200 rpm, dried at 80 °C to remove the solvent	Dried powders were heated in a tube furnace from 1000 to 1300 °C for 3 h under Ar flow with a flow rate of 300 mL min^−1^	Anatase/Rutile/Ti_9_O_17_ at 1100 °C	133
			Ti_5_O_9_/Ti_4_O_7_/λ-Ti_3_O_5_ at 1200 °C	
	Anatase TiO_2_ and polydopamine (PDA) mixed in 1 : 1 mass ratio, the mixture dipped in tris–buffer solution and stirred for 48 h. TiO_2_@PDA composites obtained by centrifugation were washed with water and ethanol and oven dried at 80 °C for 12 h	Heated in a tube furnace from 900 to 1200 °C under Ar flow (300 mL min^−1^ flow rate) for 1 h	Anatase/Ti_4_O_7_/λ-Ti_3_O_5_ at 900 °C	133
			Ti_4_O_7_/λ-Ti_3_O_5_ at 1000 °C	
	TiO_2_ and synthetic graphite mixed with distilled water and carboxymethyl cellulose and dried overnight at 120 °C. The mixture was formed into pellets by uniaxial hydraulic press	Reduction was conducted under H_2_, Ar and He atmospheres (1.00 NL min^−1^) in a fixed bed reactor in a vertical tube furnace for 5 h	Under H_2_ flow: mixtures of TiO_2_/Ti_8_O_15_/Ti_4_O_7_	134
			Under Ar flow: mixtures of TiO_2_/Ti_9_O_17_/Ti_8_O_15_/Ti_5_O_9_/Ti_4_O_7_	
	Mixtures of TiO_2_/carbon black were ball milled using 4.0 wt% carbon content	Heated in vacuum furnace from 800 to 1100 °C for 2 h	At 1000 °C: Ti_9_O_17_/Ti_7_O_13_/Ti_6_O_11_	135
			At 1025 °C: Ti_4_O_7_/Ti_5_O_9_	
	A paste formed with TiO_2_, carbon black, water and organic binders was air oxidised at 350 °C for 2 h	Sintering at 1300 °C for 2 h under Ar atmosphere	Ti_4_O_7_/Ti_5_O_9_	138
	—	Inside the tube of a tube furnace, a crucible containing anatase TiO_2_ is positioned between two crucibles containing activated charcoal. Then the samples are reduced at 1100 °C for 2–12 h	At 6 h: >90% Ti_9_O_17_	30
			7–8 h: mixtures of Ti_9_O_17_/Ti_8_O_15_/Ti_7_O_13_/Ti_6_O_11_	
			9 h: mixture of Ti_9_O_17_/Ti_8_O_15_/Ti_7_O_13_/Ti_6_O_11_/Ti_5_O_9_	
			10–12 h: phase reversal resulting in mixtures of Ti_9_O_17_/Ti_8_O_15_/Ti_7_O_13_/Ti_6_O_11_	
Rutile TiO_2_	TiO_2_, polyvinylpyrrolidone and water were mixed with ultrasonic radiation followed by drying and placing in a silica tube surrounded by carbon	Heated at 950 °C for 30 min by 2.45 GHz microwave irradiation under Ar atmosphere introduced into the furnace at 0.5 L min^−1^	Ti_4_O_7_ nanoparticles (60 nm)	139
	TiO_2_ and PVA were mixed 50/50 mass ratio	Heated at a rate of 5 °C min^−1^ at 1100 °C for 1 h under N_2_ atmosphere with a flow rate of 50 mL min^−1^	Ti_4_O_7_	29
TiO_2_ powder	Green-body pellets were formed *via* uniaxial pressing of a ball-milled mixture of TiO_2_, polyethylene glycol and distilled water. These pellets were then sintered at 1300 °C for 4 h in air	TiO_2_ tablets were covered with carbon black in alumina crucibles and heated at 1300 °C (120 °C h^−1^) for 4–6 h under Ar flow	Ti_4_O_7_/Ti_5_O_9_/Ti_6_O_11_ and residual TiO_2_	93
TiO_2_ powder	TiO_2_ fibres were formed *via* thermoplastic extrusion using stearic acid as the pre-coating material and polyethylene as the binder	TiO_2_ fibres embedded in carbon black powder were reduced at 1200 °C and 1300 °C from 1 to 24 h in an Ar atmosphere	Mixture of Ti_10_O_19_, Ti_9_O_17,_ Ti_8_O_15_, Ti_6_O_11_, Ti_5_O_9_, Ti_4_O_7_, Ti_3_O_5_ and TiO_2_	92
TTIP	Ti hydroxy oxide (Ti(OH)_*x*_O_*y*_ was formed by the reaction between TTIP with HCl vapour. This precipitate was dispersed in glucose (C/Ti ratio = 1.07)) and dried at 50 °C	Calcined at 1000 °C for 2.5 h in a vacuum chamber (slow cooling to 500 °C followed by quenching)	Ti_4_O_7_	140
	Amorphous TiO_2_ is precipitated *via* TTIP and mixed with PVA powder	Heated from 700 to 1000 °C for 1 h under N_2_ atmosphere with a flow rate of 40 mL min^−1^	Ti_4_O_7_ with trace amounts of anatase and rutile	141
Titanium ethoxide	Hybrid solutions of titanium ethoxide (in absolute dry ethanol) mixed with polyethylene imine (PEI) or polyethylene glycol (PEG) were prepared followed by electrospinning the solutions	Heated from 700 to 1000 °C for 4 h under N_2_ atmosphere at a heating rate of 5 °C min^−1^	PEG/Ti (800 °C): Ti_4_O_7_/TiN	80
			PEI/Ti (900 °C): Ti_8_O_15_	
			PEI/Ti (1000 °C): Ti_6_O_11_/Ti_5_O_9_	
	Titanium ethoxide and PEG mixed with ethanol to form a gel followed by heating at 100 °C for 4–6 h	Heated at 950 °C under Ar stream with a heating rate of 4 °C min^−1^	Ti_4_O_7_	142

The main trend in the materials formed *via* carbothermal reduction is that lower Magnéli phases are more prevalent when higher temperatures and longer durations are used for the reduction. However, for carbothermal reduction, the next most important consideration is the ratio between the Ti source and the carbon source. Toyoda *et al.* synthesised carbon-coated Magnéli phase titanium suboxides by heat-treating mixtures of rutile TiO_2_ and PVA.^29^ A 95/5 ratio of TiO_2_/PVA at 1100 °C for 1 h resulted in a mixture of rutile and Ti_9_O_17_. However, upon reducing this ratio, lower Magnéli phases were obtained, including Ti_5_O_9_ and Ti_6_O_11_ at an 80/20 ratio, and complete Ti_4_O_7_ was achieved at a 50/50 ratio. Further reducing this ratio resulted in lower titanium oxides, including Ti_2_O_3_ and Ti_3_O_5_, implying the ability to control the generated titanium suboxide phase with the reductant ratio. Similar studies were conducted by Huang *et al.*, where glucose was used as the carbon source to reduce titanium hydroxy oxide (Ti(OH)_*x*_O_*y*_).^140^ The C/Ti ratio was varied from 6.49 to 1.07. At the highest C/Ti ratio, TiO was obtained in the product heat-treated at 1000 °C for 4 h; at a 2.17 ratio, TiO_2_ was obtained as the product after annealing at 1000 °C for 2.5 h; and at a 1.07 ratio, Ti_4_O_7_ was obtained as the product under the same conditions. These studies demonstrate that in carbothermal reduction, the temperature of heat treatment, time of annealing and the C/Ti ratio are the most important design considerations for obtaining a desired product. However, similar to hydrogen reduction, no studies have been conducted on the effect of gas flow rate, highlighting further research opportunities in this area.

As mentioned earlier, carbothermal reduction addresses many concerns associated with hydrogen reduction, particularly the high costs and safety risks related to the use and storage of hydrogen gas. However, the main drawback of carbothermal reduction is the extensive pretreatment required, involving time-consuming mixing of the TiO_2_ source with the carbon reductant. In addition, direct contact between TiO_2_ and the carbon source could also yield in unwanted byproducts such as TiC and TiO_*x*_C_*y*_, which need to be separated from Magnéli phases prior to their use. To address these issues, our group introduced a novel carbothermal reduction method that eliminates the need for pretreatment or mixing with a carbon source. Instead, this method relies on CO_(g)_ generated under low-oxygen conditions to reduce TiO_2_ and form Magnéli phases.^30^ While the method successfully produces the desired Magnéli phases, the resulting particle sizes were in the micrometre range, limiting their potential applications. Therefore, further development of a carbothermal reduction approach that requires less/no precursor pretreatment while achieving nanoparticulate Magnéli phases remains an area of opportunity.

### 3.3 Metallothermic reduction

To eliminate post-synthesis purification steps for the removal of any unreacted metal oxides or carbonaceous by-products resulting from conventional reduction methodologies, metallothermic reduction has been used to synthesise titanium suboxides. This method makes use of metals or metal hydrides to reduce TiO_2_ at high temperatures.^143^ In this method, TiO_2_ is mixed with a reductant (either a metal or metal hydride) and the resulting mixture is heat-treated at high temperatures (typically 500 °C or above) for a certain duration, usually under an inert atmosphere, to obtain Magnéli phases. While metallothermic reduction minimises most of the post-synthesis purification compared to carbothermal reduction, the byproducts vary depending on the reductant used. When metal hydrides are used, H_2_ is evolved as a byproduct during the reaction. However, when metals are used as reductants, metal oxides are formed as byproducts along with the Magnéli phases. Additionally, metals like Ti and Zr, though effective as reductants, are expensive, making them unsuitable for large-scale synthesis due to the high associated costs. Therefore, there is still room for improvement in metallothermic reduction methods that do not rely on costly metals as reductants.

**Fig. 11:**
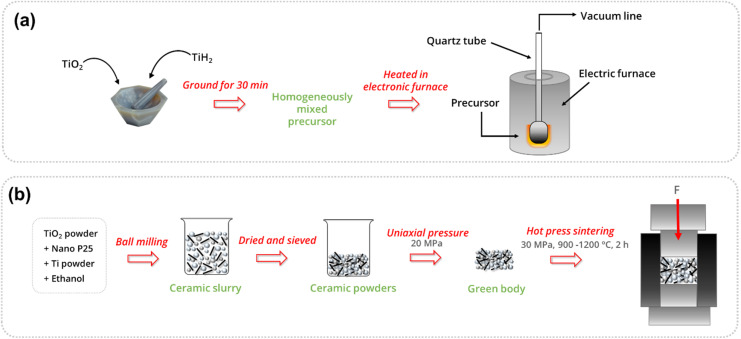
(a) Schematic representation of the experimental route for the synthesis of titanium suboxides in an electric furnace under vacuum and (b) schematic diagram of the *in situ* hot press sintering technique used to synthesise Ti_4_O_7_.

Strobel *et al.* obtained Ti_9_O_17_ crystals and smaller crystals of Ti_8_O_15_ and Ti_6_O_11_ upon the reaction of TiO_2_ with Ti powders in a two-zone furnace using Cl_2_ as the transporting agent. Here it was observed that the oxygen pressure is a critical parameter in crystal growth and the temperature gradient not only influences the growth rate but also the nature of the growing phase.^75^ Nagao *et al.* reported the synthesis of Magnéli phase titania (Ti_4_O_7_ and Ti_8_O_15_), lower titanium suboxides (TiO, Ti_2_O_3_ and Ti_3_O_5_) and metallic Ti, in solid phase reaction of rutile TiO_2_ with TiH_2_ using varied TiO_2_/TiH_2_ ratios, treatment times and temperatures (Fig. 11(a)).^143^ The presence of TiH_2_ causes the generation of metallic Ti by evolving H_2_ at higher temperatures (∼500 °C) (eqn (6)). This metallic Ti then rapidly reacts with TiO_2_ in the mixture to give titanium suboxides of varied deficiencies (eqn (7)).

6 TiH_2_ → Ti + H_2_

7 Ti + (2*n* − 1)TiO_2_ → 2Ti_*n*_O_2*n*−1_

Using these conditions, Ti_4_O_7_ has been prepared with a TiO_2_/TiH_2_ ratio of 3, by treating at 600 °C for 48 h, whereas for Ti_8_O_15_, a ratio of 5 has been used at 600 °C for 72 h.^143^ Geng *et al.* synthesised Ti_4_O_7_ through *in situ* hot-pressed sintering of Ti and TiO_2_ in a single-step process. A mixture of anatase TiO_2_, nano P25 and Ti powders in various ratios was prepared using ethanol as the solvent. After ball milling, a ceramic slurry was obtained, which, upon drying, yielded ceramic powders. These powders were compacted into green bodies through uniaxial pressing to ensure proper loading into graphite molds. The green bodies were then hot-press sintered under a 30 MPa load at varying temperatures (900–1200 °C) for 2 h under vacuum (Fig. 11(b)). The optimal sample, with a Ti/TiO_2_ weight ratio of 0.09, sintered at 1000 °C, exhibited superior mechanical properties and an excellent electrical conductivity of 1129 S cm^−1^.^144^

**Fig. 12:**
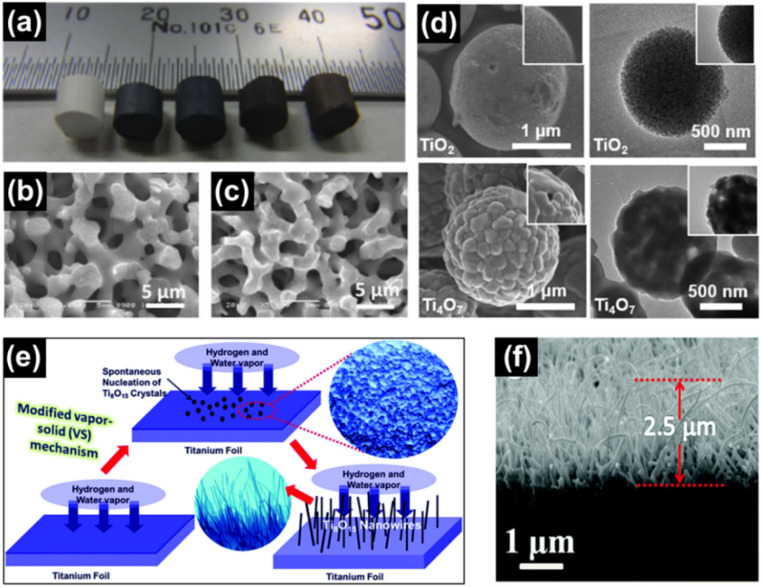
(a) Digital image of anatase TiO_2_, Ti_6_O_11_, Ti_4_O_7_, Ti_3_O_5_ and Ti_2_O_3_ monoliths (left to right) prepared using a sol–gel approach. SEM images of macroporous monoliths of (b) anatase TiO_2_ and (c) Ti_4_O_7_. Reprinted with permission from ref. 81. Copyright 2012, American Chemical Society. (d) SEM images (top left and bottom left) and TEM images (top right and bottom right) of TiO_2_ and Ti_4_O_7_ microspheres, respectively. Reprinted with permission from ref. 145. Copyright 2021, Elsevier. (e) Schematic illustration of the vapour–solid mechanism used for the growth of Ti_8_O_15_ nanowires and (f) SEM image of the cross section of the Ti_8_O_15_ nanowires. Reprinted with permission from ref. 146. Copyright 2015, Royal Society of Chemistry.

Anatase TiO_2_ monoliths have been reduced using a zirconium getter to form single phase Ti_6_O_11_, Ti_4_O_7_, Ti_3_O_5_ and Ti_2_O_3_ with varied amounts of Zr between 1000 and 1180 °C at a heating rate of 100 °C h^−1^ for 1 day reaction time (Fig. 12(a)–(c)).^81^ The formation of porous Ti_*n*_O_2*n*−1_ monoliths is expressed in eqn (8).

8 2*n*TiO_2_ + Zr → 2Ti_*n*_O_2*n*−1_ + ZrO_2_

Liu *et al.* made use of a mixture of mesoporous TiO_2_ and Ti powder (7 : 1 mol%) to synthesise Ti_4_O_7_ microspheres by heat treating at 850 °C for 3 h under Ar (Fig. 12(d)).^145^ A mixture of anatase TiO_2_ and Ti_4_O_7_ was obtained when CaH_2_ was used as the reductant to reduce P25 TiO_2_ (Degussa) when reacted at 500 °C for 5 h.^147^ Gusev *et al.* prepared single and multi-phase Ti_4_O_7_ and Ti_6_O_11_ oxides by reacting rutile TiO_2_ with Ti. Ball milling was used for mechanical activation of the starting materials followed by annealing under Ar atmosphere at 1060–1080 °C for 4 h to obtain highly conducting ceramics.^148^ He *et al.* reduced TiO_2_ powders with the use of chemically polished Ti foil. The Ti foil substrate was placed 6 cm away from the quartz reactor containing TiO_2_ in a tube furnace followed by heating at 1050 °C under H_2_ flow for 2 h. During the reaction, reduction of TiO_2_ by H_2_ forms H_2_O (g), which together with H_2_ (g) react with Ti to form a layer of compact Ti_8_O_15_ nanoparticles on the substrate (Fig. 12(e) and (f)).^146^

### 3.4 Other synthesis approaches

#### 3.4.1 Using NH_3_

Decomposed NH_3_ was used as the reducing agent to synthesise a series of multi-phased Magnéli titania by using rutile TiO_2_ as the starting material. It has been observed that highly conductive Ti_4_O_7_, Ti_5_O_9_ and Ti_6_O_11_ have been obtained when the reactant was reduced under a mixture of N_2_ and H_2_ gases (obtained from instantaneously decomposed NH_3_) at temperatures between 1000 and 1100 °C.^149^ Furthermore, Gou *et al.* reports the identification of Ti_9_O_17_ as a by-product in reducing TiO_2_ powders under an NH_3_ atmosphere at 1000 °C.^150^

#### 3.4.2 Using plasma as the energy source

**Fig. 13:**
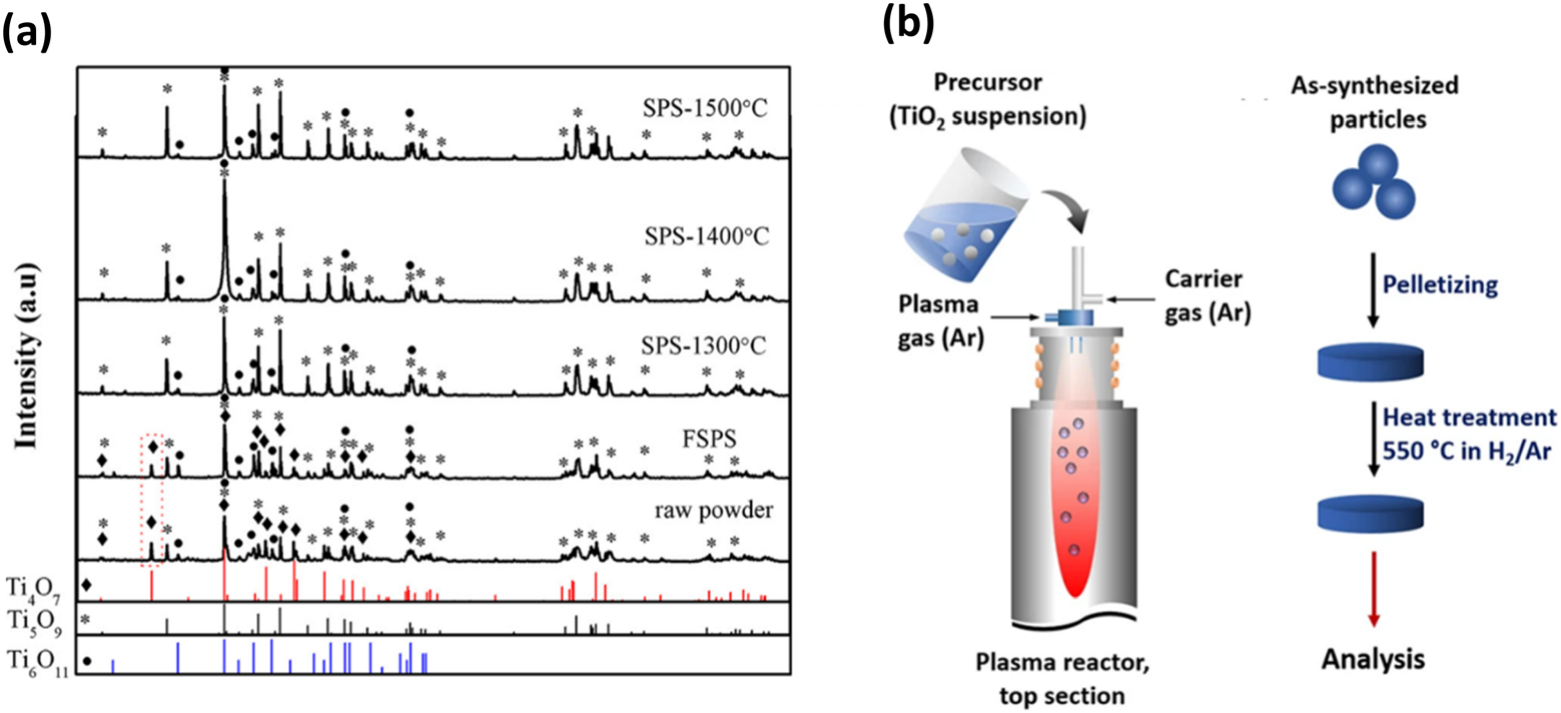
(a) XRD patterns of raw powder, FSPS sample and samples prepared by conventional SPS at different temperatures.^153^ (b) Preparation of TiO_*x*_ nanoparticles using radiofrequency (RF) induction thermal plasma method (left) and heat treatment of the samples after synthesis to improve their electrical conductivity (right).^13^

Zhang *et al.* used spark plasma sintering (SPS) to densify 1–2 μm sized Ti_4_O_7_ powdered particles to full density.^151^ Ti_4_O_7_ electrodes with a relative density of 70.3% were synthesised using spark plasma sintering at 700 °C and 30 MPa.^152^ Complete densification of Magnéli phase titania using flash spark plasma sintering (FSPS) was performed on titanium suboxide powders with agglomerates of 50–500 μm size. Results revealed that compared to conventional spark plasma sintering, FSPS was more reliable for densification since rapid sintering contributed to the retention of original phases Ti_4_O_7_, Ti_5_O_9_ and Ti_6_O_11_ without any oxidation afterwards. The XRD pattern of the FSPS sample (Fig. 13(a)) was similar to that of the raw powder, whereas in the patterns of the SPS samples, the characteristic Ti_4_O_7_ peak at 2*θ* = 20.7° had disappeared.^153^

Wang *et al.* also used SPS to fabricate Magnéli phase titanium oxides *via in situ* reduction with carbon powder and observed that the reduction can be easily achieved at low pressure and temperatures above 1200 °C and pointed out that a stable sintering environment without any pressure change due to gas reaction is vital for the reaction.^154^ SPS has been used as a densification method for Ti_4_O_7_ and Ti_8_O_15_ nano-powders by Conze and co-workers, in which the samples were heated at 1300 °C for 5 min at a heating rate of 100 °C min^−1^ under 60 MPa pressure using graphite dies lined with carbon foil.^155^ Lee *et al.* made use of an atmospheric plasma spray process to fabricate Magnéli phase titanium oxide deposits (*n* = 4–9), using TiO_1.9_ as the feedstock powder and by varying the hydrogen gas ratio to allow the tunability in nonstoichiometry and phase content.^100^ Xu *et al.* synthesised Magnéli phase titanium suboxides and lower suboxides (*n* = 2, 3) using flash synthesis by thermal plasma by varying the input power, feeding rate of the precursor H_2_TiO_3_, and the H_2_/Ar ratios in the gas mixture, which was used as the carrier gas.^156^ A radiofrequency (RF) induction plasma method was used by Arif and co-workers to form nanoparticles composed of TiO_2_, Ti_8_O_15_, Ti_4_O_7_ and lower titanium oxide phases. In their synthesis, a mixture of rutile TiO_2_ and water-isopropyl alcohol mixture was fed into the plasma reactor that made use of Ar both as the plasma and the carrier gas. To improve the conductivity of these samples, the as synthesised samples were pelletised and annealed in a vacuum furnace under 3% H_2_ (Ar) atmosphere (Fig. 13(b)).^13^

#### 3.4.3 Further synthesis approaches

Ertekin *et al.* used cathodic electrochemical deposition to form a Magnéli phase titanium oxide film on indium tin oxide glass. Deposition was conducted using a peroxo-titanium solution bath containing TiOSO_4_ and 30% H_2_O_2_ in acetonitrile at different temperatures, ranging from −10 to 40 °C.^157^ Langlade *et al.* made use of UV laser irradiation to form titanium oxide films with Magnéli structure. In a typical synthesis, a pure Ti substrate was dipped in a solution of titanium(iv) isopropoxide (which was used as the metal precursor) in isopropanol and heated at 850 °C for 1 or 4 h after deposition. These coated materials were then moved in front of a frequency tripled Nd:YAG laser, emitting 355 nm, at different speeds to form Magnéli phase titanium suboxide coated metallic surfaces.^158^ Matsuda *et al.* made use of an oxidation approach to synthesise Magnéli titanium oxide films using Ti metal as the precursor. The method they used involved heating a thick foil of titanium metal in air at a temperature of 700 °C for a duration of 3 h, and then heating it to a temperature of 900 °C without any intervening period of rest under an oxygen partial pressure of 1.0 × 10^−10^ atm. This resulted in a Ti_4_O_7_ film on a Ti metal foil, capable of absorbing visible and near IR light.^159^ Sun *et al.* introduced controlled amounts of Magnéli phase suboxides (Ti_8_O_15_ and Ti_6_O_11_) and lower oxides (Ti_3_O_5_) into titania nanotubes by anodizing Ti sheets in a two-electrode system followed by annealing at 450 °C in a N_2_ atmosphere. Formation and existence of Magnéli phases at such low temperatures have been ascribed to two potential reasons. Firstly, the annealing process in N_2_ atmosphere, which is considered more favourable for Ti^4+^ reduction over an Ar atmosphere. Secondly, the presence of nonstoichiometric titanium oxides during anodisation is another plausible explanation, as these oxides may retain their structure during subsequent thermal treatment.^160^

## 4. Potential applications of Magnéli phase titanium suboxides

**Fig. 14:**
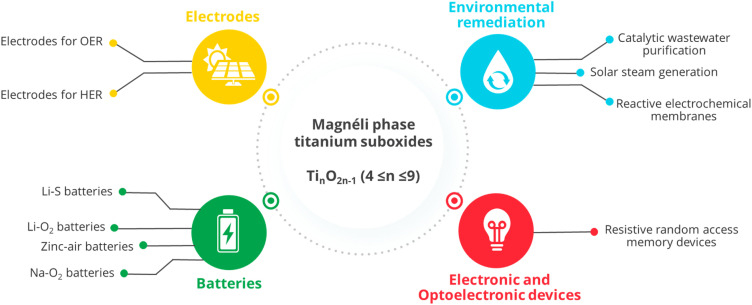
Potential applications of Magnéli phase titanium suboxides in environmental remediation and energy storage.

Magnéli phase titanium suboxides have been studied for their potential application in a variety of fields: as electrocatalysts and photocatalysts for environmental remediation, electrodes for oxygen and hydrogen evolution and as support materials in electronic and optoelectronic devices Fig. 14, as will be discussed in the following sections. Ebonex® is the registered trademark for a range of electrically conducting and corrosion resistant suboxides of titanium, mainly Ti_4_O_7_ and Ti_5_O_9_. Ever since its discovery and commercialisation, Ebonex® has been widely used in applications that require high electrical conductivity, corrosion resistance and oxidation resistance.^27^ Due to the presence of highly conducting Magnéli phases, its electrical conductivity reaches up to 300 S cm^−1^, comparable with that of carbon. It is projected that at room temperature in a 4 mol dm^−3^ H_2_SO_4_ solution, the half-life of Ebonex® is 50 years.^20^ Due to the unique structure of Magnéli phase titanium suboxides, the TiO plane at the edges connect with the TiO_2_ plane *via* sharing faces, producing a conductive band that is enclosed by TiO_2_. Hence, the conductivity in Ebonex® is as a result of the TiO layers while the chemical resistance is attributed to the TiO_2_ planes that shield the TiO layer.^27^ Ebonex® is widely used as an electrode owing to its electrochemical stability in both acidic and basic media, high electrical conductivity and high overpotentials for O_2_ and H_2_ evolution.^161,162^ However, Ebonex® itself possesses several disadvantages that make it impractical for use without alteration. One of the drawbacks is its tendency to undergo passivation through anodic polarisation, leading to an increase in the oxygen content beyond the stoichiometric value, forming titanium(iv) oxide over time. Furthermore, the material demonstrates slow transfer kinetics, necessitating surface modification or deposition of foreign materials such as noble metals and conductive oxides, for it to be used as an efficient catalyst support.^142,163^

### 4.1 Oxygen evolution and hydrogen evolution reactions

Photoelectrochemical water splitting is regarded as a highly desirable approach for generating sustainable hydrogen fuel, as it allows for the direct decomposition of water into hydrogen and oxygen through the absorption of sunlight.^164^ Electrochemical or photoelectrochemical water splitting occurs through two half reactions: oxygen evolution reaction and hydrogen evolution reaction occurring at the anode and cathode, respectively. Depending on the reaction conditions, these two half reactions are as follows.^165^

In acidic solution:Cathode: 2H^+^ + 2e^−^ → H_2_^2^ + 2e^-^Anode: H_2_O → 2H^+^ + ½O_2_ + 2e^−^In neutral solution:Cathode: 2H_2_O + 2e^−^ → H_2_ + 2OH^−^Anode: H_2_O → 2H^+^ + ½O_2_ + 2e^−^In alkaline solution:Cathode: 2H_2_O + 2e^−^ → H_2_ + 2OH^−^Anode: 2OH^−^→ H_2_O^+^ + ½O_2_ + 2e^−^

Hence, in photoelectrocatalytic water splitting, electrons facilitate the reduction of water molecules, leading to the formation of H_2_, while holes participate in the oxidation of water, resulting in the formation of O_2_ simultaneously.^166^

Conventional catalysts used in water splitting in acidic medium include iridium and ruthenium-based catalysts for oxygen evolution and Pt based materials for hydrogen evolution. Due to the scarcity and high cost of these materials, attention has been drawn to the development of cost effective alternatives.^165^ In all catalysts involved in oxygen and hydrogen evolution, the supporting material must have high electric conductivity and corrosion resistance. While carbon or graphite are commonly employed as catalyst support materials, there is emerging interest in considering Magnéli phase titanium suboxides such as Ebonex®, as catalyst supports. This is due to their notable properties including excellent oxidation resistance, high electrical conductivity (approximately 10^3^ Ω^−1^ cm^−1^), high overpotentials for oxygen and hydrogen evolution in both acidic and basic environments, and remarkable electrochemical stability. These physicochemical properties make Magnéli phase titanium suboxides promising candidates for catalytic applications.^27,161^

#### 4.1.1 Oxygen evolution reaction

The oxygen evolution reaction is the anodic reaction of water electrolysis. Studies have shown that the presence of V_O_ in transition metal oxides could favour the oxygen evolution reaction due to changed surface and bulk electronic structures that can in turn, change the surface adsorption properties and bulk conductivity.^47^ Owing to the presence of V_O_ in its structure, Magnéli phase titanium suboxides (or its commercial form, Ebonex®) have been used as a common candidate in these reactions.

Slavcheva *et al.* showed that PtCo/Ebonex® synthesised *via* a borohydride reduction method demonstrated better performance in the oxygen evolution reaction in alkaline medium, compared to Pt/Ebonex® and unsupported PtCo. The catalytic efficiency of these three electrodes was demonstrated by normalising the anodic current relative to the Pt content in the active layer, presented as mass activity (mA mg_Pt_^−1^). The curves showed that the electrochemical behaviour of PtCo/Ebonex was significantly superior to the other two electrodes, despite containing the lowest amount of Pt (0.83 mg cm^−2^). This facilitated the oxygen evolution reaction to a greater extent compared to pure Pt/Ebonex (5 mg cm^−2^) and unsupported PtCo (3.3 mg cm^−2^), proving the positive influence of the support on catalytic activity. The enhanced activity of the bimetallic catalyst was attributed to surface oxide formation and electronic interactions between the metals and the catalyst support material.^167^ Similarly, compared to polycrystalline Pt, a Pt/Ebonex® thin film loaded Au disk showed enhanced catalytic activity for the oxygen reduction reaction in acidic solution, due to the interaction of O_2_ with the active sites on the surface of the catalyst.^161^ They conducted rotating disk electrode measurements to compare the behaviour of Ebonex/Pt and pure Pt electrodes during the oxygen reduction reaction in 0.5 mol dm^−3^ HClO_4_ solution. The obtained polarisation curves showed that the onset of O_2_ reduction and the half-wave potentials were significantly shifted to more positive potentials in the case of the Ebonex/Pt electrodes, indicating higher catalytic activity for O_2_ reduction compared to pure Pt. Stoyanova *et al.* measured the water splitting electrocatalytic activity of Pt–Fe/Ebonex® and Pt–Co/Ebonex® catalysts using cyclic voltammetry and steady state polarisation. Results revealed that these two catalysts show enhanced performance for the oxygen evolution reaction in polymer electrolyte membrane water electrolysis compared to pure Pt. Best catalytic properties were observed in the 2Pt : 3Co/Ebonex® (2 : 3 = weight ratios of precursors) with the oxygen evolution reaction reaching current densities of 230 mA cm^−2^ at 1.9 V. Higher activity for bimetallic-Ebonex® catalysts is attributed to the solid solution formed between the two metals causing changes in the electron density in the atoms and surface intermediate bond strength and stability of Ebonex® at high anodic potentials.^168^ Won *et al.* developed a bifunctional oxygen catalyst using Ti_4_O_7_ capable of both oxygen reduction reaction and oxygen evolution, to enhance the efficiency of a unitised regenerative fuel cell. The optimised oxygen catalyst, synthesised by depositing PtIr on a Ti_4_O_7_ support (60 wt% metal catalysts on Ti_4_O_7_) using the borohydride reduction method, showed the highest specific activity of 1.50 mA cm^−2^ at 1.5 V, compared to Pt/Ti_4_O_7_ and Pt/C, which exhibited specific activities of 0.26 and 0.15 mA cm^−2^, respectively. Moreover, PtIr/Ti_4_O_7_ demonstrated the lowest Tafel slope of 82.3 mV dec^−1^, compared to 104.5 and 107.3 mV dec^−1^ for Pt/Ti_4_O_7_ and Pt/C, respectively, highlighting its significantly superior oxygen evolution reaction activity. The enhanced performance was attributed to the synergistic effect of the PtIr phase that had a suitable composition for both oxygen evolution and oxygen reduction reactions and high stability of the Ti_4_O_7_ support in acidic medium.^169^

**Fig. 15:**
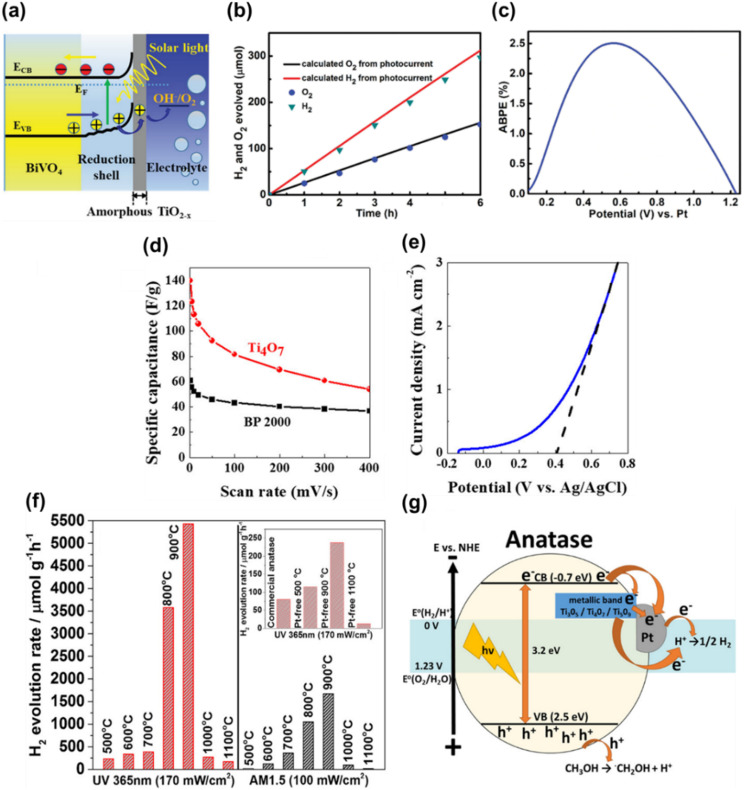
(a) Energy diagram for the transit and transfer of photogenerated charges on the b-BiVO_4_/TiO_2−*x*_ photoanode. (b) H_2_ and O_2_ generated by the b-BiVO_4_/TiO_2−*x*_ photoanode at 1.23 V_RHE_ and calculated H_2_ and O_2_ amounts evolved from the photocurrent assuming 100% faradaic efficiency. (c) Applied bias-to photocurrent efficiency (APBE) of the b-BiVO_4_/TiO_2−*x*_ photoanode obtained for solar water splitting. Reprinted with permission from ref. 172. Copyright 2019, John Wiley and Sons. (d) Specific capacitance *vs.* scan rate for the Ti_4_O_7_ electrode and Black Pearls 2000 carbon black electrode (BP 2000). (e) Linear sweep voltammetry curve for Ti_4_O_7_ electrodes in 0.1 M KOH solution. Reprinted with permission from ref. 140. Copyright 2018, American Chemical Society. (f) Photocatalytic hydrogen evolution from powders synthesised at temperatures 500–1000 °C under 365 nm LED and AM 1.5 solar simulator illumination. Inset: experiment conducted for anatase and powders obtained by the same synthesis method but without Pt. (g) Schematic diagram showing the band alignment between TiO_2_, Magnéli phase titanium suboxides and Pt. Reprinted with permission from ref. 173. Copyright 2019, American Chemical Society.

With the intention of developing non-platinum electrocatalysts for water splitting, Paunović and co-workers developed a Co/Ebonex® catalyst and tested it for oxygen and hydrogen evolution. The activity of the catalyst for hydrogen evolution was less than that of other electrocatalysts such as Vulcan XC-72 + TiO_2_ or activated multi-walled carbon nanotubes, due to the low surface area of the Magnéli phase titanium suboxides in Ebonex®. However, the Co/Ebonex® electrode showed better performance for oxygen evolution compared to CoPt/Ebonex® catalytic systems. The observed improvement is attributed to the formation of oxides on the surface of the catalyst and metal–support interaction, causing Ebonex® to act as the support material and as an active oxide electrode.^170^ Jović *et al.* studied the use of electrodeposited Ni-(Ebonex®/Ir) coatings as anode materials for the oxygen evolution reaction in alkaline solution. They observed that the pure Ni coating exhibited a drastic loss of activity after 24 h of continuous oxygen evolution at *j* = 50 mA cm^−2^ (Δ*E* = 395 mV), despite initially showing higher intrinsic catalytic activity for the oxygen evolution reaction compared to the composite coatings. In contrast, the oxygen evolution reaction overpotential of the Ni-(Ebonex/Ir) coatings showed only negligible changes after the stability test (Δ*E* = 22 mV). The OH species that were adsorbed by the active sites in Ni were transferred to the less active IrO_2_ particles, causing both the inherent activity and improved retention of catalytic activity of Ni-(Ebonex®/Ir) coatings.^171^ An amorphous TiO_2−*x*_ layer containing Ti_4_O_7_ and lower titanium oxides (Ti_2_O_3_) was employed to fabricate a photoanode of black BiVO_4_@amorphous TiO_2−*x*_ that showed a remarkable photocurrent density of 6.12 mA cm^−2^ at 1.23 V_RHE_ for water oxidation and 2.5% applied bias photon-to-current efficiency for solar water splitting. The ability of the TiO_2−*x*_ layer to act as an oxygen evolution catalyst, a protection layer to catalyse the water oxidation reaction and to prevent dissolution of BiVO_4_, promotes the efficient water splitting demonstrated by the BiVO_4_/TiO_2−*x*_ photoanode (Fig. 15(a)-(c)).^172^

Huang *et al.* synthesised a series of titanium suboxides (Ti_2_O_3_, Ti_3_O_5_, Ti_4_O_7_) with high surface areas (between 260 and 120 m^2^ g^−1^) *via* a combined sol–gel and carbothermic processes. The highest specific capacitance of 140 F g^−1^ was observed for the Ti_4_O_7_ electrode, with a high oxygen oxidation current above 0.5 V (Fig. 15(d) and (e)). A higher specific capacitance demonstrated the outstanding pseudocapacitance behaviour of Ti_4_O_7_, making it a promising electrochemical electrode material.^140^ Kolbrecka *et al.* studied the effect of porosity on the properties of Ti_4_O_7_ electrodes. Results revealed that the porosity of the electrodes affects the anodic current densities and the course of oxygen evolution, which is irreversible on Magnéli phase titanium suboxide electrodes. Furthermore, they also observed that the more reproducible results and high anodic current densities are obtained for the ceramic electrodes that have a high number of large pores.^120^

#### 4.1.2 Hydrogen evolution reaction

Similar to other evolution reactions, the hydrogen evolution reaction requires a significant overpotential, making it crucial to identify suitable electrocatalysts to maximise efficiency. Noble metals like Pt, Pd and Ru are among the most effective electrocatalysts for hydrogen evolution due to their low overpotential. Out of these, Pt is currently regarded as the best material, due to its ability to optimally adsorb hydrogen, which facilitates both the adsorption of active hydrogen species and the desorption of hydrogen molecules. However, the high cost and limited catalytic stability of these materials in acidic and alkaline electrolytes have prompted the search for alternative catalysts.^174,175^

Weirzbicka *et al.* reported the use of anatase TiO_2_ and Magnéli phase titanium suboxides loaded with Pt nanoparticles for photocatalytic H_2_ evolution. The optimal mixed-phase particles, containing 32% anatase, 11% rutile and 57% Magnéli phases, loaded with 290 ppm of Pt, exhibited a highly efficient photocatalytic H_2_ evolution rate of ∼5432 μmol h^−1^ g^−1^ under UV light and 1670 μmol h^−1^ g^−1^ under AM 1.5 conditions. This was approximately 50–100 times more efficient than anatase with similar Pt loading. The enhanced photocatalytic performance of the optimised nanoparticles under both solar irradiation and UV light (*λ* = 365 nm) was attributed to the synergy of charge carrier formation by anatase and charge carrier separation and mobility by Magnéli phase titania (Fig. 15(f) and (g)).^173^ Nanoscopic inserts of Magnéli phase titanium suboxides in anatase TiO_2_ nanocrystals have shown to work as efficient cocatalysts in H_2_ generation when suspensions of these nanoparticles were used in H_2_O/methanol solutions. The mixed-phase particles optimised at 900 °C, consisting of 30% anatase, 25% Ti_4_O_7_ and 20% Ti_5_O_9_, achieved a direct photocatalytic H_2_ evolution rate of 145 μmol h^−1^ g^−1^ under AM 1.5 solar-simulated light, without the use of a cocatalyst. In comparison, pure anatase or Magnéli phases exhibited significantly lower photocatalytic H_2_ evolution performance. The researchers further elaborate that the activity in these mixed particles is due to the synergistic effect of anatase TiO_2_ acting as the light absorber and Ti_4_O_7_ as the mediator for charge separation, transport and transfer, leading to significant H_2_ generation without the need of an external cocatalyst.^125^ A Au(111)@Ti_6_O_11_ heterostructure synthesised *via* photoreduction showed excellent hydrogen evolution activity with a low overpotential of 49 mV at 10 mA cm^−2^ current density. Furthermore, it also showed 18 times higher mass activity (9.25 A mg_Au_^−1^*vs.* 0.51 A mg_Pt_^−1^) and similar stability in acidic media compared to commercial Pt/C (20 wt%). Successful heterostructure formation enabling Au nanoparticles to better adsorb H^+^, increasing the number of active sites on the catalyst and the conductive Ti_6_O_11_ support material were determined as factors that lead to better performance in the Au(111)@Ti_6_O_11_ catalyst.^176^

### 4.2 Wastewater purification

#### 4.2.1 Electrooxidation of organic pollutants

Electrooxidation, an advanced oxidation method, has emerged as a promising approach for degrading organic contaminants in wastewater due to its high mineralisation efficiency, mild treatment conditions, relatively simple equipment and environmentally friendly nature.^177^ In electrooxidation, organic contaminants are removed in a two-step process. First, a direct electron transfer occurs from the contaminant (R) to the anode (eqn (9)) followed by generation of high amounts of highly active OH˙ through water discharge at the surface of the anode material (M), which has a high O_2_ overpotential (eqn (10)). Generated OH˙ actively participate in degrading a diverse range of organic compounds, as given in eqn (11).^178,179^

9 R → (R˙)^+^ + e^−^

10 M + H_2_O → M (OH˙) + H^+^ + e^−^

11 R + M (OH˙) → degradation by-products

**Fig. 16:**
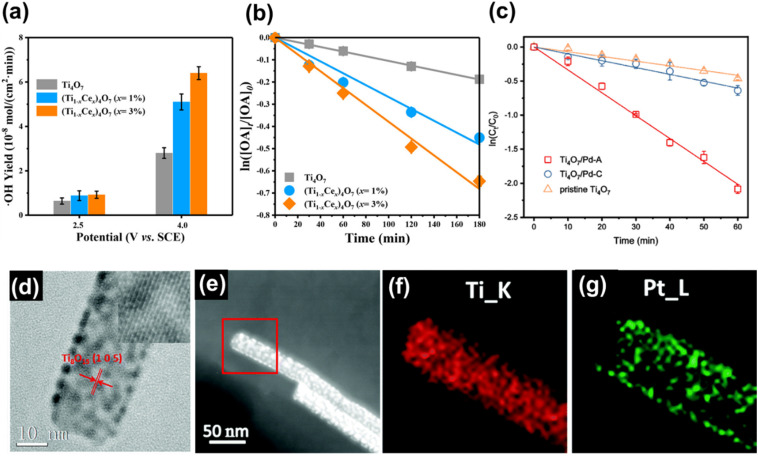
(a) Hydroxyl radical yield at different potentials and (b) oxidation of oxalate under anode polarisation at 2.0 V *vs.* saturated calomel electrode by Ti_4_O_7_ and (Ti_(1−*x*)_Ce_*x*_)_4_O_7_ electrodes. Reprinted with permission from ref. 181. Copyright 2021, American Chemical Society. (c) Pseudo first order kinetics of PFOA degradation with pristine Ti_4_O_7_, Ti_4_O_7_/amorphous Pd and Ti_4_O_7_/crystalline Pd electrodes. Reprinted with permission from ref. 184. Copyright 2020, American Chemical Society. (d) High resolution TEM (HRTEM) image, (e) high-angle annular dark-field scanning transmission electron microscopy (HAADF-STEM) image, (f) and (g) HRTEM-STEM energy dispersive X-ray spectroscopy (EDS) mapping images of Pt/Ti_8_O_15_ nanowires. Reprinted with permission from ref. 146. Copyright 2015, Royal Society of Chemistry.

Boron doped diamond (BDD) has been a common anode material in such reactions but has high fabrication cost. This has moved attention to the use of materials such as Magnéli phase titanium suboxides, which have low fabrication costs, high oxygen evolution potential (>2.5 V *vs.* standard hydrogen electrode (SHE)) and chemical inertness.^179,180^ Magnéli titania-based electrodes have been used to successfully degrade and inactivate various organic compounds including per- and polyfluoroalkyl substances (PFASs), perfluorooctanoic acid (PFOA), phenol, *N*-nitrosodimethylamine, antibiotics and pathogens.^181–183^ However, studies have shown that the yield of OH˙ radicals generated by Magnéli phase titania-based electrodes is lower compared to that by conventional electrodes such as BDD and PbO_2_, due to its low interfacial charge transfer rate. To overcome this issue and to gain better performance, pure phase titanium suboxides such as Ti_4_O_7_ were introduced with the inclusion of varying amounts of foreign elements such as C, amorphous Pd clusters and Ce^3+^ (Fig. 16(a)–(c)).^181,184^

You *et al.* studied the use of monolithic porous Ti_4_O_7_ electrodes for the electrochemical oxidation (degradation) of organic pollutants in industrial dyeing and finishing wastewater. Their results confirmed that the Ti_4_O_7_ electrode removed the chemical oxygen demand (COD) and dissolved organic carbon (DOC) by 66.5% and 46.7% respectively, at 8 mA cm^−2^ current density after 2 h of reaction. Furthermore, the bioavailability of wastewater was improved, with the ratio between the five-day biological oxygen demand: COD increasing from 0.029 to 0.28 after treatment, making it a more cost effective and environment friendly material for electrode preparation compared to PbO_2_ and SnO_2_ based electrodes.^22^ Wang *et al.* investigated the successful degradation and deactivation of antibiotics, antibiotic resistant bacteria and antibiotic resistance genes present in raw wastewater *via* electrooxidation using a Magnéli phase Ti_4_O_7_ anode. Multidrug-resistant *Salmonella enterica* serotype Typhimurium DT104 was fully inactivated, achieving a 6.2 log reduction within 15 minutes at a current density of 10.0 mA cm^−2^. Additionally, antibiotic resistance genes such as *TetG*, *floR* and *sul1*, along with the class 1 integron gene (*intI1*) and virulence genes (*invA* and *spvC*) within the pathogen, were reduced by 99.65% to 99.94%. In the same electrochemical oxidation treatment system, the model antibiotics tetracycline and sulfadimethoxine were degraded by 97.95% and 93.42%, respectively, within 3 h.^185^ When used for electrochemical oxidation of perfluorooctanesulfonic acid (PFOS), the nano-Ti_4_O_7_ anode showed a better PFOS degradation rate and energy efficiency in both batch and REM operations compared to a micro-Ti_4_O_7_ anode and commercial Ebonex® (Ti_9_O_17_) anode due to its favourable pore size distribution and composition. The study found that the electroactive surface area of the anodes is linked to pores larger than 1.03 μm, suggesting a pore size threshold that limits electrochemical accessibility in Magnéli phase porous titanium suboxide materials. As a result, anodes with pore sizes just over 1 μm are likely to offer the most effective surface area for electrochemical reactions.^186^

A Ti_4_O_7_ anode was also used for the effective removal of tetracycline by electrochemical oxidation, due to the high conductivity and chemical stability of the material. For tetracycline removal, applying current densities between 0.5 and 3 mA cm^−2^ achieved over 90% removal across initial concentrations ranging from 1 to 50 ppm, with half-lives of 28 to 75 minutes. They further found that hydroxyl radicals generated on Ti_4_O_7_, at a rate of 2 × 10^−9^ mol cm^−2^ min^−1^ under 0.5 mA cm^−2^, contributed to at least 40% of the total tetracycline removal. Tests on *Escherichia coli* (*E. coli*) cultures confirmed that electrooxidation by the Ti_4_O_7_ anode reduced tetracycline's antimicrobial activity to undetectable levels.^182^ A film containing a 1 : 1 TiO_2_ : Ti_4_O_7_ mixture prepared on polymethyl-meta-acrylate spectroscopy cuvettes showed effective photoanodic performance in decolourising methylene orange dye compared to TiO_2_ and Ti_4_O_7_ separately. The photoelectrochemical dye degradation tests indicated that TiO_2_ and Ti_4_O_7_ achieved decolourisation values of 35% and 46% respectively, while the TiO_2_/Ti_4_O_7_ mixed film achieved 53% decolourisation. The enhancement in composite film performance is attributed to the synergistic effect of photocatalytic and electrochemical activities shown by TiO_2_ and Ti_4_O_7_, respectively.^187^ Geng *et al.* synthesised highly ordered Ti_4_O_7_ nanotube arrays and examined their electrooxidation ability. In electrooxidizing phenol, Ti_4_O_7_ nanotube arrays showed a 1.7 times higher degradation coefficient compared to BDD, demonstrating their suitability for electrooxidation applications.^180^ Ti_4_O_7_ nanotube arrays achieved a 95.3% removal of phenol chemical oxygen demand, outperforming Ti_4_O_7_ particles (79.4%). This enhanced performance was attributed to the presence of Ti_4_O_7_ particulates in the nanotube arrays and their higher surface area (11.7 m^2^ g^−1^) compared to Ti_4_O_7_ particles (4.7 m^2^ g^−1^). When used for the electrochemical methanol oxidation reaction, Pt loaded Ti_8_O_15_ nanowires prepared by an electrodeposition method showed a peak current density of 28.2 mA cm^−2^ compared to 20.5 mA cm^−2^ and 17.2 mA cm^−2^ for Pt/C and Pt/TiO_2_ electrodes, respectively, demonstrating the best performance for the Magnéli titania supported electrocatalysts (Fig. 16(d)–(g)).^146^ A Sc_2_O_3_-Magnéli phase titanium composite electrode was prepared by Bai and co-workers using a sintering-pressing technique that demonstrated 90.16% degradation of methyl orange after 120 min of electrolysis. Optimal electrocatalytic activity was determined with a current density of 10 mA cm^−2^, solution pH 3 and temperature of 25 °C.^188^

The interaction of Ebonex® with the deposited metal could alter the activity or nature of the metal electrocatalyst, as studied by Dieckmann and Langer. When tested for electrogenerative oxidation of aliphatic and aromatic alcohols and formaldehyde, Pt/Ebonex® showed higher activity for methanol oxidation compared to Ni/Ebonex®, while the latter showed significant activity for formaldehyde oxidation and was slightly more polarised for benzyl alcohol.^189^ Chen *et al.* investigated the use of an Ebonex® ceramic anode for electrolytic oxidation of trichloroethylene and observed that CO_2_ was primarily formed with traces of CO, and no other carbon containing products.^190^ Under an anodic potential (*E*_a_) of 2.5 to 4.3 V *vs.* silver-silver chloride electrode, trichloroethylene degradation followed first-order kinetics with respect to its concentration. The oxidation rate was pH-independent between pH 1.6 and 11. Trichloroethylene oxidation occurred exclusively on the anodic surface and became mass transport-limited at higher potentials (*E*_a_ > 4.0 V). Despite the strong anodic performance of Magnéli phase electrodes, Jing *et al.*^191^ observed a decline in the electrochemical activity of Ti_*n*_O_2*n*−1_ electrodes during anodic polarisation. This was attributed to be dependent on the type of electrolyte, either due to the formation of a surface TiOSO_4_ passivating layer or by the loss of charge carriers. Though this surface deactivation can be reversed during the discharge process, the associated ohmic drop requires pre-conditioning of the cell.^191,192^

#### 4.2.2 Photocatalytic degradation of wastewater pollutants

Semiconductors have been widely used in the photocatalytic degradation of wastewater pollutants, including waterborne pathogens, antibiotics, pharmaceuticals, dyes and other industrial effluents. This is due to the low cost of semiconductor materials, their high pollutant degradation rate, low toxicity and the ability to completely mineralise organic contaminants into non-toxic products such as H_2_O and O_2_. In semiconductor photocatalysis, when light with energy greater than the bandgap of the semiconductor is absorbed, it excites electrons from the VB to the CB, generating electron–hole pairs. These electrons and holes participate in a series of oxidation and reduction reactions, producing reactive oxygen species, which degrade the organic contaminants in wastewater.^193,194^ TiO_2_ is a regularly used semiconductor in photocatalytic wastewater purification due to its high abundance, non-toxicity, strong oxidising ability, low cost and chemical stability. Photocatalytic degradation of organic contaminants occurs with absorption of UV light by TiO_2_, which eventually leads to the formation of highly active superoxide (O_2_˙^−^) and hydroxyl (OH˙) radicals. These radicals along with photogenerated holes oxidise organic contaminants resulting in less toxic by-products.^41,195^ Unlike UV light-active TiO_2_, Magnéli phase titanium suboxides demonstrate visible light photosensitivity, due to the V_O_ induced during phase transformation from rutile to Magnéli.^29^ However, as described above, most Magnéli phase titanium suboxides synthesis approaches require high temperature annealing, resulting in micrometre-sized particles with poor surface area having less adsorption capacity, thus limiting its photocatalytic activity.^33,196^ To address this issue, Magnéli titania has been combined with different materials such as C, Pt or g-C_3_N_4_ to improve the surface properties.^29,196^

**Fig. 17:**
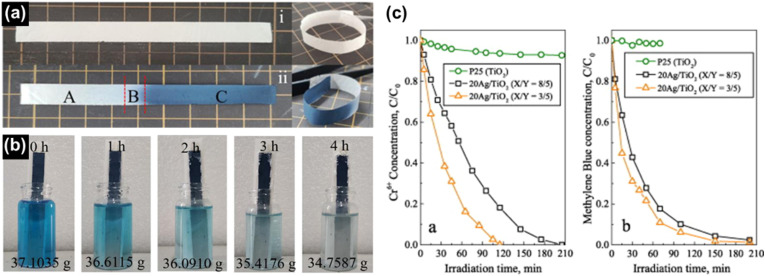
(a) Digital images of the (i) prepared pristine TiO_2_ and (ii) gradient titanium oxide films and (b) photographs of the change in simulated sewage with time with the treatment of gradient titanium oxide films. Reprinted with permission from ref. 197. Copyright 2022, American Chemical Society. (c) Photocatalytic reduction of Cr^6+^ ions (left) and methylene blue degradation (right) under visible light irradiation by TiO_2_ (commercial P25) and 20Ag/TiO_2_ prepared by flame spray pyrolysis (FSP) at *X*/*Y* = 3/5 and *X*/*Y* = 8/5. *X* is the rate at which the precursor solution containing TTIP, silver acetate and acetonitrile was fed through the FSP nozzle, which was then dispersed to a fine spray by *Y* = 5 mL min^−1^ oxygen. Reprinted with permission from ref. 198. Copyright 2014, Elsevier.

Si *et al.* synthesised gradient titanium oxide nanowire films composed of Ti_*n*_O_2*n*−1_ (composed of Ti_5_O_9_, Ti_4_O_7_ and Ti_3_O_5_) and TiO_2−*x*_ (anatase TiO_2_ with traces of rutile TiO_2_) (Fig. 17(a)) by an electrostatic spinning method and gradient temperature annealing, which showed excellent photothermal conversion efficiency and high photodegradation ability against simulated sewage (Fig. 17(b)). The film completely degraded 0.02 g L^−1^ methylene blue dye in 90 min under 2 suns and achieved a water evaporation rate of 1.833 kg m^−2^ h^−1^ under 1 sun.^197^ Similarly, Fujiwara *et al.* showed that TiO_2_ nanostructures containing layers of Ti_4_O_7_ and lower titanium suboxides (Ti_3_O_5_) with different Ag loadings demonstrated high photoactivity in Cr^6+^ reduction and methylene blue degradation under visible light (*λ* > 400 nm) (Fig. 17(c)). The improved performance compared to pristine materials has been attributed to the strong visible light activity of the composite due to the sub-bandgap energy tails generated from the suboxides.^198^ Li and co-workers synthesised TiO_2_–Ti_5_O_9_ nanostructures *via* a one-step laser ablation in liquids approach, using varying pulse energy densities during synthesis. The optimised Ti_5_O_9_–TiO_2_ heterojunction formed between the metal oxide phases exhibited enhanced visible light photocatalytic degradation of rhodamine B dye (*λ* ≥ 420 nm), nearly completely removing the dye within three hours of light irradiation. This improved performance was attributed to efficient charge separation at the phase junction and increased light absorption across a broader wavelength range.^199^

Zhao *et al.* synthesised a high surface area carbon layer coated Ti_4_O_7_ and g-C_3_N_4_ composite *via* a wet chemical and high temperature treatment route (C@Ti_4_O_7_/g-C_3_N_4_), with its surface area 5 times higher (72.97 m^2^ g^−1^) than pristine Ti_4_O_7_ (14.83 m^2^ g^−1^). Photocatalytic degradation studies on rhodamine B, methylene blue and methyl orange under visible light showed that the activity and cyclability of C@Ti_4_O_7_/g-C_3_N_4_ outperformed that of pristine Ti_4_O_7_, C@Ti_4_O_7_ and anatase TiO_2_/g-C_3_N_4_ catalysts. The superior activity of the core–shell structured composite catalyst was attributed to inherent V_O_, efficient light absorption, a large specific surface area and enhanced charge carrier separation efficiency.^196^ Carbon-coated Magnéli phase titanium suboxides, prepared by heat-treating rutile TiO_2_ with PVA at 1100 °C under N_2_ flow, were studied for the photocatalytic decomposition of iminoctadine triacetate and phenol under visible light (*λ* > 400 nm). However, results showed that lower titanium suboxides, Ti_2_O_3_ and Ti_3_O_5_, had better photocatalytic activity compared to higher *n* Magnéli phases (*n* = 4, 5, 6, 9), implying that the presence of a higher concentration of V_O_ is favourable for the photocatalytic activity under visible light.^29^

### 4.3 Reactive electrochemical membranes

Traditional membrane filtration faces several challenges, such as relying solely on physical separation for removing toxic pollutants, membrane fouling, decreased permeate flux, chemical usage and high energy consumption. In contrast, reactive electrochemical membranes (REMs) integrate electrochemical advanced oxidation processes (EAOPs) with physical filtration, creating a single, multifunctional water treatment system. REMs generate reactive oxygen species through EAOPs to disinfect organic contaminants without producing harmful by-products.^178^ Similar to the electrooxidation of organic pollutants discussed in Section 4.2.1, water in reactive electrochemical membranes is oxidised to produce OH˙ radicals on the anode surface, as shown in eqn (12) below.

12 H_2_O →OḢ + H^+^ + e^−^

These OH˙ engage in oxidative reactions with various organic contaminants, leading to their complete degradation or conversion to different byproducts. However, for certain recalcitrant compounds that exhibit low activity towards OH˙ (such as fluorinated organics), direct oxidation can also take place during anodic oxidation. In this process, an electron is transferred from the contaminant (R) to the anode, as was shown in eqn (9).^200^

REMs can effectively remove waterborne pathogens and heavy metals from drinking water. Their use minimises the need for pretreatment, simplifies operation, reduces chemical consumption and lowers both process and operational costs.^201^ By combining EAOPs and microfiltration, REMs also mitigate membrane fouling during filtration or backwashing, enhancing flux recovery.^26^

Given the similarity in the working principles of anodic oxidation for organic pollutants, as discussed in Section 4.2.1, and REMs, the types of materials commonly employed are also similar. The efficiency of the process is highly dependent on the choice of anode material. Several anode materials, such as BDD, Ti/RuO_2_, SnO_2_ and PbO_2_, have been extensively explored for REMs. Among these, BDD has demonstrated the highest efficiency and lowest energy consumption (65 kW h kg per COD^1^). However, due to the high production costs involved with BDD, there has been a search for alternative anode materials for REMs.^202^ Magnéli phases, particularly Ti_4_O_7_, have attracted attention in this regard due to their excellent stability, high oxygen evolution potential and superior electrical conductivity.

Zaky and Chaplin demonstrated the potential use of porous and tubular Ti_4_O_7_ as REMs through the removal of a series of *p*-substituted phenolic pollutants. Their studies showed that these REMs were effective for both anodic oxidation and OH˙ generation, facilitating efficient removal of the phenolic compounds. Their cross-flow filtration studies, supported by DFT calculations, revealed that *p*-benzoquinone was mainly removed through reactions with electrochemically produced OH˙. In contrast, the removal of *p*-nitrophenol and *p*-methoxyphenol was mainly influenced by the anodic potential applied during the process.^203,204^ To improve the flexibility of REMs, Santos *et al.* used an electrospinning and electrospraying method that resulted in highly porous and flexible REMs, composed of polysulfone fibres and Ti_4_O_7_ particles. Membrane filtration experiments showed that the observed first order rate constant for phenol oxidation was 2.6 times higher in filtration mode compared to cross flow mode at a 1.0 mA cm^−2^ current density.^205^ Studies have shown that the use of REMs as cathodes prevents the formation of halogenated organic compounds that are typically produced during electrochemical oxidation and advanced oxidation processes. Magnéli phase titania samples have been investigated as suitable candidates for this purpose because of the low cost of porous monolithic structure formation and their ability to form OH˙ *via* water oxidation.^26^

**Fig. 18:**
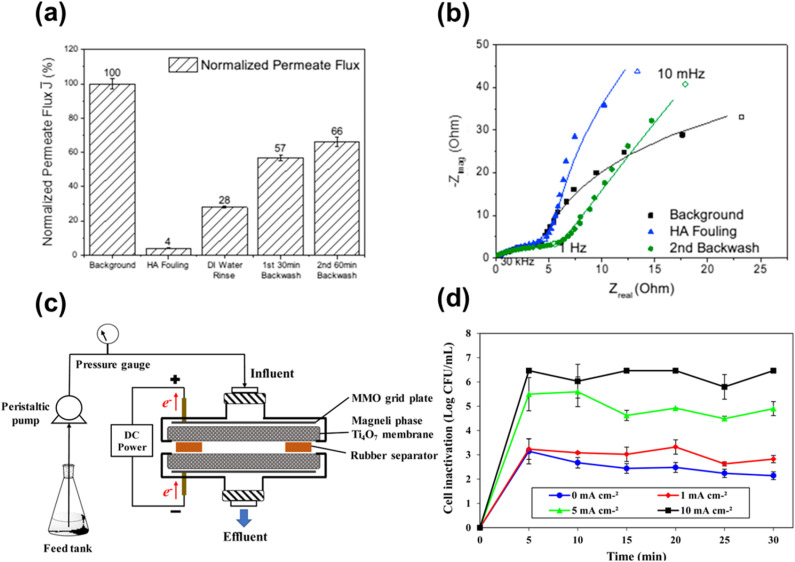
(a) Normalised permeate flux under different operational conditions for the anodic chemical-free electrochemical regeneration in backwash mode of 150 mg L^−1^ humic acid fouled REM and (b) electron impedance spectra obtained in the complete frequency range. Reprinted with permission from ref. 124. Copyright 2016, Elsevier. (c) REM system containing Ti_4_O_7_ ceramic membrane design and setup. (d) Effect of different current densities on the removal of *E. coli* by the Ti_4_O_7_ membrane filtration system. Reprinted with permission from ref. 207. Copyright 2018, Elsevier.

As discussed above, Ti_4_O_7_ is the most conductive Magnéli phase, generating the highest amount of OH˙.^206^ However, to further improve OH˙ generation, many studies have focussed on modifying Ti_4_O_7_ either by increasing the electrochemical surface area or by doping or forming composites with other electrocatalysts.^123^ Jing *et al.* synthesised a ceramic, asymmetric, ultrafiltration REM composed of Ti_4_O_7_ and Ti_6_O_11_ Magnéli phases and tested their activity using humic acid and polystyrene beads as model foulants. Membrane fouling was characterised using an electrochemical impedance spectroscopy technique. A backwash mode chemical-free electrochemical regeneration process was developed based on the results of the above analyses and enabled complete recovery of a fouled membrane without the need for any chemical reagents (Fig. 18(a) and (b)).^124^ Degradation of sulfamethoxazole using electrochemical reduction and oxidation in single pass, flow through mode using Ti_4_O_7_ REMs and Pd–Cu doped Ti_4_O_7_ REMs were studied by Misal and co-workers. An impressive 96.1 ± 3.9% of sulfamethoxazole was removed by the Pd–Cu/Ti_4_O_7_ REM *via* electrochemical reduction at −1.14 V/SHE, higher than that observed for Ti_4_O_7_ and Pd/Ti_4_O_7_ REMs. However, in electrochemical oxidation, the Ti_4_O_7_ REM showed the highest removal, removing 95.7 ± 1.0% sulfamethoxazole at 2.03 V/SHE.^122^ Adsorption and electrochemical reduction of *N*-nitrosodimethylamine by carbon–Ti_4_O_7_ composite REMs was studied by Almassi and co-workers. Upon the addition of multi-walled carbon nanotubes or activated carbon to the REM, the residence times of *N*-nitrosodimethylamine in the REM increased by a factor of 3.8 to 5.4, leading to higher degradation.^183^ To study the electrochemical inactivation of *E. coli* at different current densities, Liang *et al.* studied a REM system with two highly conductive, stable and porous Ti_4_O_7_ membrane electrodes, working in dead-end filtration mode (Fig. 18(c)). Their studies showed that as the current density increased, the bacterial concentration decreased, with severe damage to the cells. Moreover, it is reported that the *E. coli* concentration decreased from 6.46 to 0.18 log CFU mL^−1^ after passing through the membrane filter (Fig. 18(d)).^207^ To treat agriculturally contaminated water, Gayen and co-workers modified Ti_4_O_7_ REMs by depositing B-doped SnO_2_ to provide high overpotential in oxygen evolution. When atrazine and clothianidin were used as the model contaminants and terephthalic acid as the OH˙ probe, complete mineralisation of all compounds was achieved at 3.5 V/SHE in a single pass in the reactor with a 3.6 s residence time.^121^

### 4.4 Electronic and optoelectronic applications

The operation of resistive random-access memory (ReRAM) is based on the dielectric breakdown of an insulator, mostly metal oxides, which shows resistive switching phenomena. In the structure of a ReRAM, the insulator/semiconducting material is sandwiched between two metal electrodes. In the resistive switching process of an ReRAM, by applying pulsed voltages, the resistance of the cell can be changed significantly.^208,209^ Hence, devices in which the resistive switching mechanism occurs demonstrate bipolar behaviour, wherein the conducting and insulating states are alternated by applying opposite bias polarities.

**Fig. 19:**
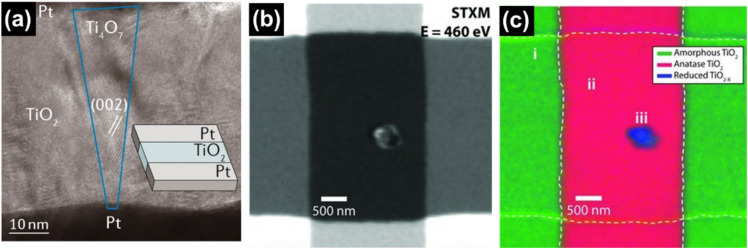
(a) HRTEM image of a nanocrystalline Ti_4_O_7_ filament (outlined in blue) in a Pt/TiO_2_/Pt system used in a resistive switching device. Reprinted with permission from ref. 217. Copyright 2020, Springer Nature. (b) Scanning Transmission X-ray Microscopy image of the device junction area, showing absorption contrast within the junction and (c) chemical and structural mapping of the three observed phases in the junction. Region (i) corresponds to amorphous TiO_2_, Region (ii) is anatase TiO_2_ and Region (iii) is reduced titanium suboxides TiO_2−*x*_. Reprinted with permission from ref. 211. Copyright 2010, John Wiley and Sons.

Kwon and co-workers probed the nanofilaments in a Pt/TiO_2_/Pt system during resistive switching directly by HRTEM. They observed that Ti_4_O_7_ conducting filaments formed in TiO_2_ implying that the formation of Magnéli phase filaments induced the observed switching (Fig. 19(a)).^210^ Similarly, Strachan *et al.* observed a Ti_4_O_7_ crystallite in the TiO_2_ matrix, showing that the resistance switching demonstrated by a Pt/TiO_2_/Pt memristor is due to TiO_2_ reduction and crystallisation of a metallic conducting network. They further explained that within a TiO_2_ matrix, the formation of Magnéli phases is thermodynamically favoured over a high concentration of randomly distributed vacancies in the material, depending on the electrochemical potential within the device (Fig. 19(b) and (c)).^211^ Kim *et al.* prepared a cross-bar type Pt/TiO_2_/Pt structure with improved electrical endurance characteristics and uniform low resistance state and high resistance state distribution in filamentary resistive switching. Ti_4_O_7_ and Ti_5_O_9_ conducting filaments were observed in the TiO_2_ thin film that lead to a stable memory window.^212^

Banerjee *et al.* demonstrated the resistive switching and complementary resistive switching behaviours in a TiO_*x*_/Al_2_O_3_-based 3D vertical crossbar ReRAM device. Stable resistive switching mechanisms in these devices were confirmed and attributed to the observation of Ti_5_O_9_ nanofilaments by HRTEM.^213^ As observed in these studies, formation of titanium suboxide Magnéli phases *via* generation of shear planes in TiO_2_ is related to the valence-change memory effect, which states that the reduction of transition metal ions is caused by migration of V_O_ driven by the electrochemical potential gradient of V_O_.^62,210–214^

### 4.5 Batteries

Rechargeable batteries, a type of electrochemical energy storage, have garnered significant attention over the past few decades due to their higher energy efficiency compared to mechanical and chemical storage systems.^215^ Among various rechargeable batteries, commercial lithium-ion batteries have become particularly popular thanks to their high energy density, cyclic stability and energy efficiency. Recently, sodium and potassium have also gained similar interest as potential alternatives, due to their comparable chemical properties to lithium.^216^ Magnéli phase titanium suboxides are used in batteries as electrodes because of their unique electronic and electrochemical properties. These materials have high electrical conductivity and high specific capacity, suggesting that they can store and release a significant amount of energy per unit mass. In addition, Magnéli phase titanium suboxides are highly stable, corrosion resistant and durable, making them excellent candidates in rechargeable batteries.^27,28^ They have also demonstrated excellent cyclability, enabling them to undergo many charge–discharge cycles without a decline in electrochemical performance. Furthermore, their high thermal stability allows these materials to withstand high temperature, improving the safety and reliability of batteries and preventing overheating and short circuiting.^23,24,218^ As discussed in Section 4.1, Magnéli phase titanium suboxides are commonly used as catalyst supports for oxygen and hydrogen evolution reactions, primarily due to their high electrical conductivity and exceptional corrosion resistance, which ensure stability in both highly acidic and basic environments and provide significant electrochemical durability. Given that similar properties are desirable for battery applications, it is likely that Magnéli phase titanium suboxides developed for catalytic purposes could be adapted and optimised for use in battery technologies as well.

**Fig. 20:**
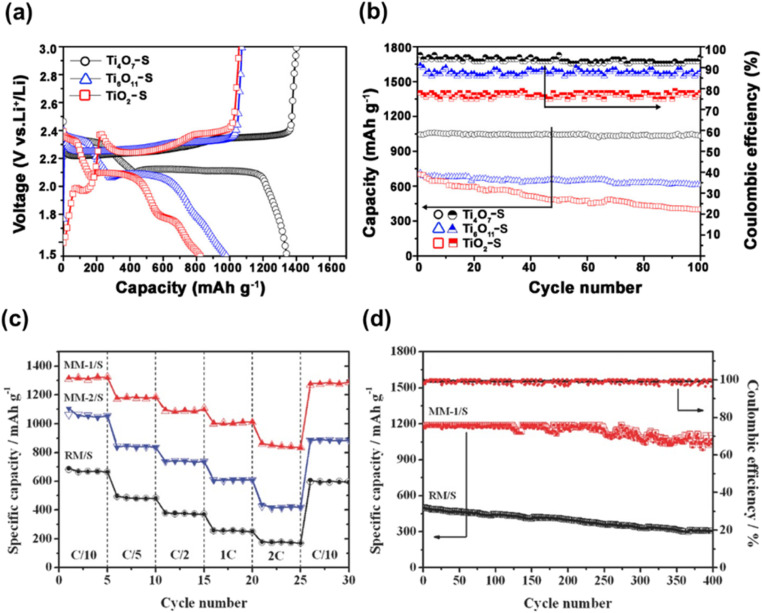
(a) Initial charge–discharge curves of composite cathodes formed with TiO_2_, Ti_4_O_7_ and Ti_6_O_11_ at a rate of 0.02C. (b) Cyclic performance and coulombic efficiency of the cathodes for 100 cycles at 0.1C rate. Reprinted with permission from ref. 219. Copyright 2014, American Chemical Society. (c) Rate performance from C/10 to 2C of rutile TiO_2_ microspheres infiltrated with S (RM/S) and Ti_4_O_7_ microspheres infiltrated with S (MM-x/S) (The molar ratio of TiO_2_ : C during synthesis varied across the samples, with a ratio of approximately 3 : 2 for MM-1, and 3 : 4 for MM-2, depending on the volume of resol solution used) and (d) cyclability with calculated coulombic efficiency of RM/S and MM-1/S at C/5. Reprinted with permission from ref. 223. Copyright 2017, John Wiley and Sons.

Ti_4_O_7_ has been investigated as a suitable host material for sulfur loading in lithium sulfur batteries due to its high conductivity and high binding affinity towards lithium polysulfide.^145^ In lithium sulfur batteries, the shuttle effect and the uncontrollable deposition of lithium sulfides have been identified as factors that can result in low coulombic efficiency and capacity decay. To find a solution to this challenge, Tao *et al.* used a sublimation and deposition method to fabricate a high performance Ti_4_O_7_–S cathode composite by thermal diffusion of S into the Ti_4_O_7_ matrix. Results revealed higher cycling performance and reversible capacity compared to the TiO_2_–S cathode, due to more effective binding of Ti_4_O_7_ with S species. The Ti_4_O_7_–S cathode demonstrated high specific capacities at different C rates, achieving 1342, 1044 and 623 mA h g^−1^ at 0.02, 0.1 and 0.5C, respectively, along with impressive capacity retention of 99% at 100C and 0.1C (Fig. 20(a) and (b)).^219^ Sabbaghi and co-workers developed highly conductive Magnéli Ti_4_O_7_ nanotube arrays supported by a carbon-coated separator, to enhance the energy density and enable rapid charging and discharging in Li–S batteries. The battery demonstrated a reversible discharge capacity of 723 mA h g^−1^ after 500 cycles with a capacity fading rate of 0.07% per cycle at 0.5C.^220^ Han and Wang reported the use of graphitic carbon coated Ti_9_O_17_ as anodes for Li-ion batteries and in hybrid electrochemical cells. These materials have shown excellent cyclic stability giving a pseudocapacitive lithium-storage behaviour with a reversible capacity of 182 mA h g^−1^.^221^ Additionally, Lee *et al.* presented the Ti_6_O_11_/carbon nanotube composite electrode as a promising anode material for K-ion batteries. This electrode exhibited an extended cycling life of over 500 cycles at a current rate of 200 mA g^−1^, with a capacity retention of 76% and an impressive coulombic efficiency of 99.9%.^222^

Wei *et al.* developed a sulfur cathode hosted on mesoporous Ti_4_O_7_ microspheres (70 wt% sulfur) that exhibited a high discharge capacity of 1317.6 mA h g^−1^ at moderate current density, with 88% capacity retention after 400 cycles, exhibiting excellent cyclability. Excellent stability attributed to the Ti–S interactions between low-coordinated Ti_4_O_7_ and lithium polysulfides, facilitated sulfur redeposition during the charging phase. Additionally, the porosity and the high electronic conductivity of the electrodes contributed to the high performance of the material.^223^ Furthermore, Li *et al.* successfully studied the feasibility of Ti_4_O_7_ for use as air–cathodes in zinc–air batteries under strong alkaline conditions due to its electrochemical durability and stability. The stability of the Ti_4_O_7_ electrode was evaluated in an O_2_-saturated alkaline solution through cyclic voltammetry within a potential range of −0.7 to +0.7 V *vs.* Hg/HgO. The Ti_4_O_7_ electrode successfully survived 5000 cycles without any significant loss in the oxygen reduction reaction peak current.^114^ Yao *et al.* used Ti_4_O_7_ as the conductive additive in a sulfur electrode and showed that sulfur/Ti_4_O_7_ had improved electrochemical properties compared to a sulfur/acetylene black electrode, in both its polysulfide absorption and its catalytic activity towards the Li/S redox reaction.^127^

Franko and co-workers utilised Ti_4_O_7_ as a stabilising additive for simple carbon paper cathodes to limit NaO_2_ degradation in sodium–oxygen batteries. Results revealed that Ti_4_O_7_ serves as a stable nucleation point for NaO_2_ formed in the solution, resulting in a reduced rate of NaO_2_ degradation. Consequently, Ti_4_O_7_-coated cathodes exhibited significantly longer cell lifetimes over many cycles compared to cathodes cast with commercial SuperP carbon black. When the Ti_4_O_7_ content in the cathode slurry (comprising Ti_4_O_7_, poly(vinylidene fluoride) and SuperP carbon in acetone) was increased to up to 90%, the cell lifetime significantly improved to 37 cycles before any notable degradation was observed.^224^ To evaluate the possibility of replacing carbon in electrodes, Lee *et al.* synthesised RuO_2_@Ti_4_O_7_ nanospheres that could enhance the performance of Li–O_2_ batteries. With Ti_4_O_7_ supporting the activation of catalytic performance of RuO_2_ nanoparticles during discharge–charge processes, these carbon-free RuO_2_@Ti_4_O_7_ nanosphere electrodes are considered as potential candidates for superior oxygen reduction and oxygenation reactions.^225^

### 4.6 Photothermal applications

Solar-driven steam generation is becoming an important approach for harnessing solar energy, as it directly converts solar energy into heat for water evaporation and facilitates energy storage.^226^ The photothermal materials used in solar steam evaporators can effectively capture solar energy and generate heat, which is then transferred to bulk water for water vapour generation.^227^ The performance of a solar steam evaporator partially depends on the photothermal material employed. Among the various types of photothermal materials, such as plasmonic nanoparticles, carbon-based materials, polymers and inorganic semiconductors, semiconductors are increasingly being modified and utilised due to their abundance and low cost.^228^ Magnéli phase titanium suboxides, with their excellent light absorbance across the solar spectrum and desirable thermal conductivity, have demonstrated high solar-to-vapour efficiency, making them suitable photothermal materials for solar steam generation.^30^

**Fig. 21:**
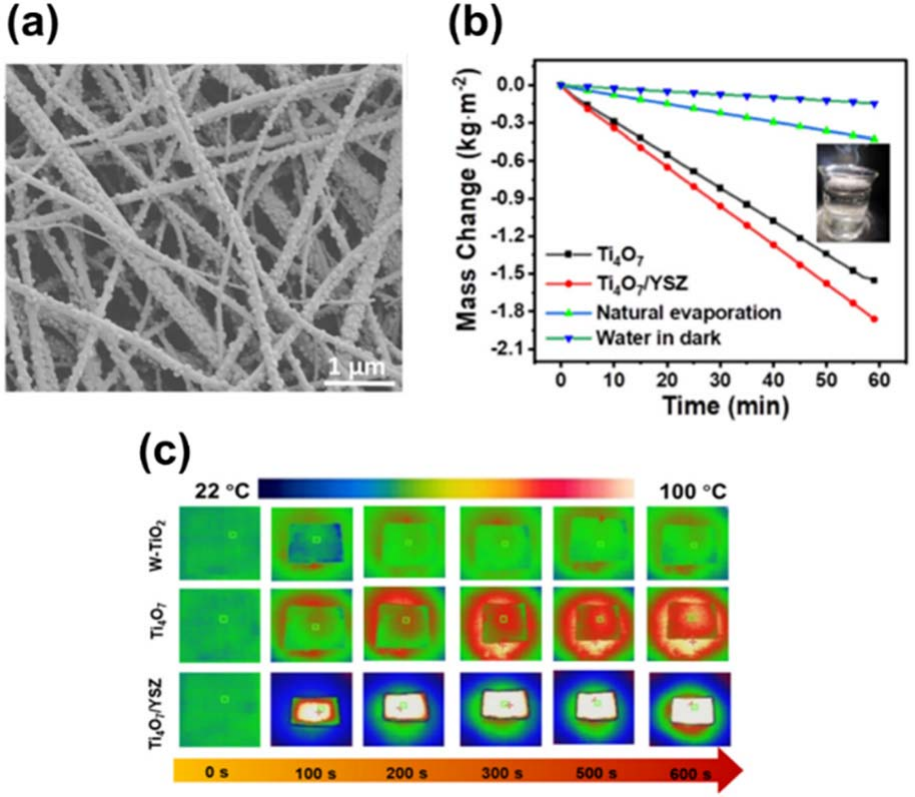
(a) SEM image of fibrous Ti_4_O_7_ membrane after calcination. (b) Rate of water evaporation from the Ti_4_O_7_ membrane with and without YSZ. (c) Temperatures on the surface of water recorded for TiO_2_, Ti_4_O_7_ and Ti_4_O_7_/YSZ membranes by an IR thermometer. Reprinted with permission from ref. 229. Copyright 2022, American Chemical Society.

The ability for Magnéli phase titanium suboxides to absorb solar light has been used in solar steam generation *via* a self-floating Ti_4_O_7_/yttrium stabilised zirconia (YSZ) membrane (Fig. 21(a)). While the insulating YSZ layer is conducive to water transportation, the upper Ti_4_O_7_ layer was used for photothermal conversion, so that the bilayered membrane showed a remarkable water evaporation rate of 1.86 kg m^−2^ h^−1^ under one sun (Fig. 21(b) and (c)).^229^ Si *et al.* synthesised gradient titanium oxide nanowire films composed of TiO_2−*x*_ and Ti_*n*_O_2*n*−1_, which exhibit both photocatalytic and photothermal properties. The Ti_*n*_O_2*n*−1_ component of the film (containing Ti_4_O_7_ and Ti_3_O_5_) demonstrated a water evaporation rate of 1.833 kg m^−2^ h^−1^ under one sun irradiation, with an energy conversion efficiency of 88.96%.^197^ Xu *et al.* prepared Ti_4_O_7_-PVA nanocomposite hydrogels that exhibit a narrow bandgap of approximately 0.81 eV for highly efficient solar steam generation. Under one sun irradiation, this hydrogel achieved an evaporation rate of approximately 4.45 kg m^−2^ h^−1^ with an energy efficiency of around 90.69%. Additionally, the hydrogel demonstrated impressive stability, maintaining an evaporation rate of up to approximately 4.03 kg m^−2^ h^−1^ until day 20. Further experiments conducted on seawater desalination revealed negligible salt accumulation on the hydrogel's surface, enabling its use in stable purification of sewage or desalination of seawater containing multiple organic contaminants.^228^ Since most Magnéli phase titanium suboxides used in solar steam evaporators are based on Ti_4_O_7_, in one of our previous studies, we investigated Magnéli phases other than Ti_4_O_7_ in a solar steam evaporator and monitored its performance. We utilised a graphene oxide-based aerogel and incorporated Magnéli phase titanium suboxides (comprising 2.8% Ti_5_O_9_, 81.8% Ti_6_O_11_, 30.7% Ti_7_O_13_, 3.8% Ti_8_O_15_ and 0.9% Ti_9_O_17_) to form a solar steam evaporator. The composite aerogel (radius 2.25 cm) demonstrated a water evaporation rate of 0.832 kg m^−2^ h^−1^ under 1.0 sun with an optimised weight of 50 mg of Magnéli phase titanium suboxide particles. The calculated energy conversion efficiency for the optimised aerogel was 56.5%, which is over three times that of water without the aerogel under the same conditions.^30^

### 4.7 Other applications

**Fig. 22:**
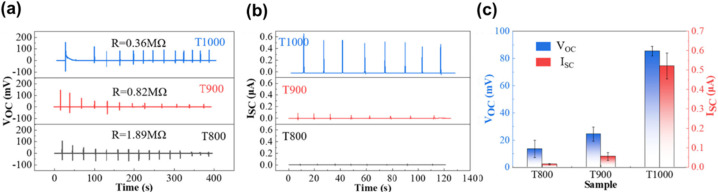
(a) Measured open circuit voltage (*V*_OC_) and (b) short circuit current (*I*_SC_) for Magnéli phase nanocrystal films prepared at different temperatures (800–1000 °C) and (c) average peak values of *V*_OC_ and *I*_SC_. Reprinted with permission from ref. 129. Copyright 2021, American Chemical Society.

Hydrovoltaics is a novel electricity generating technology that harnesses the ability of nanomaterials to respond to waves or the flow, dropping or evaporation of water.^230^ To enhance the electric power generation in a material used in hydrovoltaics, its interaction with water needs to be improved with a reduction in its internal resistance. Since V_O_ increase the carrier concentration and attract cations, Si *et al.* used Magnéli phase nanocrystal film as the active material for hydrovoltaic power generation. They observed that the open circuit voltage (*V*_OC_) and short circuit current (*I*_SC_) increased with increasing oxygen vacancy concentration (which correlated to increasing annealing temperature during material synthesis). They demonstrated the use of their energy generation devices by capturing rainwater and observed that a ∼600 mV *V*_OC_ was maintained for over 2.5 h (Fig. 22(a)–(c)).^129^

Qing *et al.* synthesised a Ti_4_O_7_/polyimide composite using a heat pressing technique, which exhibited tunable dielectric properties and excellent absorbance in the high-frequency range of the GHz band. In addition, the study also demonstrated the potential of these composites as flexible absorbing coating materials. When the polyimide matrix was backfilled with 60 wt% Ti_4_O_7_ particles, the minimal reflection loss reached −49.3 dB at 13.7 GHz with a thickness of 1.25 mm. This finding further confirmed that the tunable and unique dielectric properties of Ti_4_O_7_ make it an excellent candidate for designing absorbers and for other electromagnetic applications.^231^ The gas sensing behaviour of a Magnéli phase containing a metal oxide layer (rutile TiO_2_, Ti_9_O_17_ and Ti_8_O_15_) coated on a flexible polymeric thin film was tested using NH_3_(g) at 12.5–100 ppm. Positive results observed in the sensing experiments were attributed to the high concentration of V_O_ and porosity of the active layer, which can increase gas adsorption capacity and the surface for interaction.^232^ Fan *et al.* demonstrated the possibility of using Ti_9_O_17_ as a non-toxic n-type thermoelectric material,^77^ while Canillas *et al.* explored the use of Ti_5_O_9_ as a candidate electrode material in neuron growth stimulation due to its low impedance and high chemical stability.^233^

## 5. Challenges and prospects

### 5.1 Stability and durability

Magnéli phases are well known for their excellent chemical and electrochemical stability, making them appealing to electrochemists and electrochemical engineers. Because of these properties, Ebonex® materials have been employed both as standalone electrode materials and as substrates for various electrocatalysts.

Research conducted by Smith *et al.* explored the stability of titanium suboxides in the Magnéli phase under harsh conditions. The study found these materials to be more durable than traditional electrodes used in large-scale electrochemical processes, including both Ti and TiO_2_. Magnéli phases remained stable when exposed to aggressive chemicals like fluoride-based etchants, hydrochloric acid and aqua regia. However, they experienced some degradation in more extreme environments, such as boiling phosphoric acid, concentrated HF and highly concentrated NaOH solutions (concentration > 12 M).^25^ Liu *et al.* investigated the electrochemical stability windows of Ti_4_O_7_ electrodes across acidic, neutral and alkaline aqueous solutions. Their findings demonstrate that the stability windows in all three types of solutions exceed 3.5 V, enabling the production of significant amounts of ˙OH radicals. Notably, the Ti_4_O_7_ electrode exhibited its widest stability window of 4.19 V in a 1 M NaCl solution, while the narrowest window, 3.53 V, was observed in a 1 M H_2_SO_4_ solution.^234^

Magnéli phase titanium suboxides show better stability in various aspects compared to carbon-based materials. Ceramics produced at high temperatures tend to be in their most stable and fully oxidised state, meaning they are less likely to oxidise further, unlike metals and carbon. In addition, in applications like electrolysis, Ebonex® electrodes demonstrate greater stability than carbon electrodes, particularly at high pH levels, where carbon materials tend to decompose.^25^ Li *et al.* investigated the stability of Magnéli phase Ti_4_O_7_ electrodes for oxygen reduction in zinc–air rechargeable batteries, as carbon materials tend to degrade due to corrosion from O_2_ and H_2_O_2_, and at high electrode potentials. Ti_4_O_7_, with its excellent conductivity and electrochemical stability, emerged as a strong candidate for air–cathodes in these batteries. Cyclic voltammetry and chronopotentiometric tests confirmed its stability. Raman and XPS analysis before and after testing revealed the formation of a thin TiO_2_ layer on the Ti_4_O_7_ surface, which likely protects the bulk material from further oxidation, enhancing its long-term stability.^114^

Krishnan *et al.* describe the use of Magnéli phase titanium suboxides as durable catalyst supports capable of withstanding high potentials in PEM fuel cells. These materials demonstrate remarkable corrosion resistance, even under very high potential conditions. The electrochemical stability of the Pt/Ti_*n*_O_2*n*−1_ catalyst (with Ti_4_O_7_ as the dominant phase in Ti_*n*_O_2*n*−1_) was investigated through cyclic voltammetry at a scan rate of 50 mV s^−1^. Their research highlighted that Ti_*n*_O_2*n*−1_ exhibits excellent stability over the potential range of −0.25 to 2.75 V *vs.* SHE.^130^ Owing to their resistance to corrosion and resilience against polarity reversal, Ebonex® electrodes are also used in electrophoresis. Their chemical inertness, especially in the presence of organic electrolytes, provides flexibility in the choice of gel types . Their large surface area allows for efficient voltage and current distribution, and they perform exceptionally well in reverse or pulsed electrophoresis, due to their high stability under polarity reversal.^25^

These findings suggest that, despite being synthesised through the creation of V_O_, Magnéli phase titanium suboxides remain stable at room temperature and in oxygen-rich environments over time. Instead of instability, they exhibit remarkable stability and durability across diverse chemical conditions, reaffirming their suitability for various applications.

### 5.2 Cost–benefit analysis

As detailed in the synthesis section, TiO_2_ is the primary feedstock for many synthesis methods of Magnéli phases. Depending on the method, different reducing agents such as hydrogen gas, carbon or metals are used. Since these reduction processes typically require high temperatures over extended periods, cost considerations largely depend on the choice of raw materials and reductants, assuming operational and energy costs remain comparable.

In most reduction methods for forming Magnéli phases, TiO_2_ is the primary raw material, priced at approximately USD 3/kg globally in 2022.^235^ Considering the various reduction methods used to form Magnéli phases, the high cost of metals such as Ti (over USD 10/kg)^236^ and Zr (with an average import price of USD 28/kg in the United States in 2023) makes large-scale synthesis *via* metallothermic reduction highly impractical.

Due to the high costs associated with the storage and transportation of hydrogen, carbothermal reduction emerges as a more cost-effective method for large-scale synthesis of Magnéli phase titanium suboxides, including commercial Ebonex®. This approach is particularly advantageous as it uses activated charcoal as the reductant, which is priced at around USD 5.6/kg, based on both literature and commercial sources.^237^ Consequently, the relatively low cost of raw materials for producing Magnéli phase titanium suboxides offers these materials a significant cost-benefit advantage over more commonly used electrode materials like BDD in electrochemical applications.

### 5.3 Challenges related to the synthesis and use of Magnéli phase titanium suboxides

In the last decade, Ti^3+^ self-doped Magnéli phase titanium suboxides have attracted much interest in diverse fields due to their outstanding optoelectronic, photochemical and photocatalytic properties. The main current issues in the synthesis and use of Magnéli phase titanium suboxides are the high temperatures and extensive sintering required, which lead to particle coalescence and the formation of microparticles that are unsuitable for applications requiring high surface areas. Issues related to microparticle formation have been resolved by using diverse titanium precursors, such as Ti(NO_3_)_2_, and various polymeric reducing agents combined with microwave irradiation, leading to the synthesis of nanoscale Ti_4_O_7_.^127,238^ However, these methods still require expensive, extensive and time-consuming precursor treatments and high-temperature synthesis. To address this, several novel techniques have been developed to synthesise titanium suboxides without the need for such intense sintering. One such approach, described by Arif and co-workers, involves a thermally induced plasma process.^239^ While this method successfully produces Magnéli phase titanium suboxides with relatively higher surface area (52.9 m^2^ g^−1^) the use of plasma is expensive and limits its scalability. Moderate-temperature synthesis methods for Magnéli phase titanium suboxides have also been explored, but these often rely on costly reductants, such as metals and metal hydrides, making them impractical for large-scale production.^143^ This indicates that none of the current synthesis approaches fully meet all criteria, including nanoparticle formation, the use of low cost reductants/raw materials and the elimination of sintering. Therefore, there is a clear opportunity to develop innovative methods for synthesising nanostructured Magnéli phases at moderate temperatures without relying on costly raw materials.

**Fig. 23:**
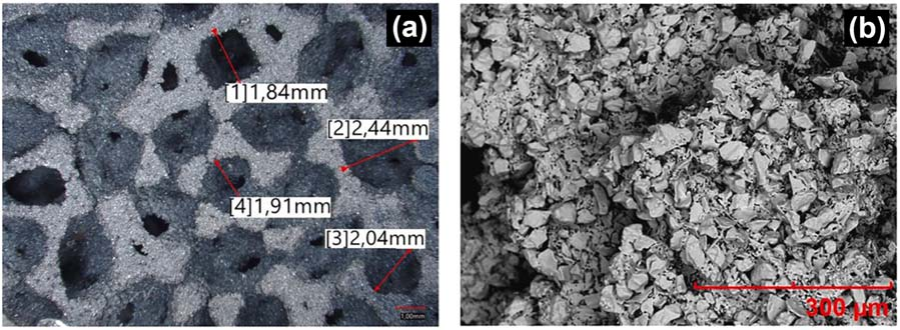
(a) Porous structure and pore size of 3D printed TiO_*x*_ electrode and (b) morphology of 3D TiO_*x*_ as observed from SEM. Reprinted with permission from ref. 240. Copyright 2023, Elsevier.

Recently, Ma *et al.* made use of 3D printing technique to synthesise a porous Magnéli phase electrode composed of Ti_5_O_9_, Ti_6_O_11_ and traces of Ti_7_O_13_, from a TiO_*x*_ powder mixture and aqueous binder (Fig. 23(a) and (b)). This 3D TiO_*x*_ electrode showed enhanced degradation kinetics for the probe molecules oxalic acid, terephthalic acid and paracetamol, lower accumulation of toxic by-products (hydroquinone and benzoquinone) and higher mineralisation yield compared to commercial BDD and Ti/TiO_*x*_ plate anodes.^240^ This study demonstrates the potential of 3D printing for creating low-energy, feasible approaches for Ti_*n*_O_2*n*−1_ preparation, offering an alternative to conventional methods requiring high temperatures or expensive raw materials.

In addition to the challenges associated with the synthesis of Magnéli phase titanium suboxides, a lesser-known aspect is the potential health risks and concerns related to these materials. It is reported that Magnéli phase materials are generated as incidental nanoparticles during industrial coal combustion causing these nanomaterials to be widespread in the environment.^241,242^ Analyses of coal ash samples obtained from powerplants in USA and China have shown that all Magnéli phases (*n* = 4–9) are generated during coal burning with Ti_6_O_11_ being the most frequent.^243^

Toxicity tests on Magnéli phase nanomaterials report that they have potential toxicity pathways that are biologically active without photostimulation.^241^ These studies report that exposure and accumulation of Magnéli phases cause abnormalities in macrophages due to increased oxidative stress and mitochondrial dysfunction and also result in reduced lung function impacting airway resistance and elastance.^242^ Kononenko *et al.* studied the hazard potential of Magnéli phase titanium suboxides on A549 human lung cells. Although some potentially adverse effects of Magnéli phase nanoparticles were observed due to their cellular internalisation and biopersistance, they were considered as non-hazardous, with no increase in intracellular reactive oxygen species levels upon exposure.^244^ Jemec Kokalj *et al.* conducted a study on hazard characterisation of Magnéli TiO_*x*_ using a set of ecotoxicological test organisms and human lung and liver cell lines. Since exposure to these test organisms and cell lines did not induce any biological response, Magnéli TiO_*x*_ particles were considered as acutely non-hazardous.^245^ To mitigate the harmful effects of these nanoparticles, some countries use particle traps to capture these nanoparticles before final emission of the exhaust gas. Further assessment regarding the impact of Magnéli phase titanium suboxide particles is required to determine the feasibility of using them in large scale applications.

### 5.4 Potential improvement strategies and prospects

As previously discussed, the 3D printing of Magnéli phase titanium suboxide electrodes offers a promising synthesis route due to its relatively low cost and time efficiency compared to conventional approaches, which often require significant energy and extensive processing time. To date, there has been only one documented instance of Magnéli phases being synthesised through additive manufacturing. This represents a significant opportunity for further research and development, particularly in the creation of Magnéli phase electrodes for various electrochemical applications. Advancing this area could lead to more sustainable and scalable production methods for energy storage and conversion technologies.

Due to the high production costs of BDD for electrochemical applications, Magnéli phase titanium suboxides and their doped counterparts are actively being investigated as alternative anode materials for the electrooxidation of organic pollutants and in REMs. While their use in these applications is currently mostly limited to the laboratory scale, we believe that electrochemical applications will soon present a promising direction for Magnéli phase titanium suboxides.

The authors of this review identified several areas related to these materials that offer significant opportunities for further development and clarification. As discussed in Section 4, photocatalytic pollutant degradation using Magnéli phase titanium suboxides has been investigated in numerous studies, with most approaches focusing on heterojunction formation between Magnéli phase Ti_*n*_O_2*n*−1_ and other materials. However, comprehensive studies on heterojunctions involving both organic and inorganic semiconductors are limited, revealing an evident research gap. Moreover, the charge transfer mechanisms in these heterojunctions, which are currently understood in terms of Z-scheme or semiconductor-to-metal charge transfer mechanisms, require deeper investigation. Integrating these experimental studies with theoretical insights could provide a broader understanding of charge transfer dynamics and open new avenues for utilising these materials in photocatalysis.

Further research could also explore the potential of Magnéli phases as standalone catalysts in photocatalytic water purification or with dye sensitisation. A key challenge in these applications is the narrow bandgap of Magnéli phases, which may limit their ability to generate reactive oxygen species. Additionally, the low surface area of these materials may hinder dye adsorption, thus reducing their efficacy. Our research has demonstrated the possibility of bandgap tuning to overcome this limitation, allowing Magnéli phases to reach the potentials required for the formation of superoxide and hydroxyl radicals, thereby enabling them to participate in photocatalytic reactions. Developing Magnéli phases with higher surface areas could further expand their applications, enhancing their utility in environmental and energy-related technologies. Implementing *in situ* characterisation could offer critical insights into the catalytic and oxidation mechanisms occurring on the surface or interface of the material across various applications. This approach may clarify real-time structural and chemical changes, thereby advancing our understanding of how Magnéli phase titanium suboxides perform under operational conditions.

Two lesser-explored applications of Magnéli phase titanium suboxides are gas sensing and hydrovoltaics. These areas hold considerable potential for industrial applications and sustainable energy generation, particularly using low-cost raw materials. Further investigation into these applications could significantly broaden the scope of Magnéli phases in environmental remediation and energy storage, contributing to the development of advanced, cost-effective solutions for global challenges.

## 6. Conclusion

This review has highlighted the numerous opportunities that Magnéli phase titanium suboxides present across various fields, including catalysis, batteries, REMs, and electronic and optoelectronic devices. The performance comparisons with the most common materials used in these applications indicate that Magnéli phases not only match but often exceed the performance of traditional materials for similar tasks.

Despite the challenges associated with synthesising these materials for large-scale applications, innovative approaches, such as 3D printing, hold great promise. If these methods can be developed and optimised to be cost-effective, Magnéli phase materials could effectively replace conventional materials due to their excellent thermoelectric properties, optical characteristics, corrosion and oxidation resistance, and their ability to form reactive oxygen species on their surfaces.

Given their rising popularity, it is anticipated that research efforts will focus on developing higher surface area Magnéli phases through low-cost synthesis strategies, thereby broadening their use in a variety of applications. This continued exploration may address current challenges and unlock new functionalities and markets for Magnéli phases, solidifying their role as a transformative material in advanced technologies.

## Data availability

No primary research results, software or code have been included and no new data were generated or analysed as part of this review.

## Author contributions

S. Amanda Ekanayake: investigation, validation, formal analysis, writing – original draft. Haoxin Mai: investigation, writing – review and editing. Dehong Chen: conceptualisation, visualisation, supervision. Rachel A. Caruso: conceptualisation, funding acquisition, writing – review & editing, supervision.

## Conflicts of interest

There are no conflicts to declare.

## References

[cit1] Guo Q. (2019). *et al.*, Fundamentals of TiO_2_ photocatalysis: concepts, mechanisms, and challenges. Adv. Mater..

[cit2] Chen X., Selloni A. (2014). Introduction: Titanium Dioxide (TiO_2_) Nanomaterials. Chem. Rev..

[cit3] Roy P., Berger S., Schmuki P. (2011). TiO_2_ Nanotubes: Synthesis and Applications. Angew. Chem., Int. Ed..

[cit4] Kaplan R. (2016). *et al.*, Simple synthesis of anatase/rutile/brookite TiO_2_ nanocomposite with superior mineralization potential for photocatalytic degradation of water pollutants. Appl. Catal., B.

[cit5] Miao R. (2016). *et al.*, Mesoporous TiO_2_ modified with carbon quantum dots as a high-performance visible light photocatalyst. Appl. Catal., B.

[cit6] Yang X. (2008). *et al.*, Synthesis of visible-light-active TiO_2_-based photocatalysts by carbon and nitrogen doping. J. Catal..

[cit7] Zhang Y. (2011). *et al.*, Engineering the Unique 2D Mat of Graphene to Achieve Graphene-TiO_2_ Nanocomposite for Photocatalytic Selective Transformation: What Advantage does Graphene Have over Its Forebear Carbon Nanotube?. ACS Nano.

[cit8] Li G.-S., Zhang D.-Q., Yu J. C. (2009). A New Visible-Light Photocatalyst: CdS Quantum Dots Embedded Mesoporous TiO_2_. Environ. Sci. Technol..

[cit9] Pan X. (2013). *et al.*, Defective TiO_2_ with oxygen vacancies: synthesis, properties and photocatalytic applications. Nanoscale.

[cit10] Nowotny J. (2007). *et al.*, Titanium dioxide for solar-hydrogen I. Functional properties. Int. J. Hydrogen Energy.

[cit11] Diebold U. (2003). The surface science of titanium dioxide. Surf. Sci. Rep..

[cit12] Malik H. (2020). *et al.*, Modelling and synthesis of Magnéli Phases in ordered titanium oxide nanotubes with preserved morphology. Sci. Rep..

[cit13] Arif A. F. (2017). *et al.*, Highly conductive nano-sized Magnéli phases titanium oxide (TiO_*x*_). Sci. Rep..

[cit14] Xu B. (2016). *et al.*, Structures, preparation and applications of titanium suboxides. RSC Adv..

[cit15] Inglis A. (1983). *et al.*, Electrical conductance of crystalline Ti_n_O_2n-1_ for n= 4–9. J. Phys. C: Solid State Phys..

[cit16] Bursill L. A., Hyde B. G. (1972). Crystallographic shear in the higher titanium oxides: Structure, texture, mechanisms and thermodynamics. Prog. Solid State Chem..

[cit17] Bennett R. A. (1999). *et al.*, STM and LEED observations of the surface structure of TiO_2_ (110) following crystallographic shear plane formation. Phys. Rev. B:Condens. Matter Mater. Phys..

[cit18] Andersson S. (1957). *et al.*, Phase analysis studies on the titanium-oxygen system. Acta Chem. Scand..

[cit19] Radecka M. (2007). *et al.*, Effect of oxygen nonstoichiometry on photo-electrochemical properties of TiO_2−*x*_. J. Power Sources.

[cit20] Kao W. H., Patel P., Haberichter S. L. (1997). Formation Enhancement of a Lead/Acid Battery Positive Plate by Barium Metaplumbate and Ebonex®. J. Electrochem. Soc..

[cit21] Bartholomew R. F., Frankl D. R. (1969). Electrical Properties of Some Titanium Oxides. Phys. Rev..

[cit22] You S. (2016). *et al.*, Monolithic Porous Magnéli-phase Ti4O7 for Electro-oxidation Treatment of Industrial Wastewater. Electrochim. Acta.

[cit23] Walsh F. C., Wills R. G. A. (2010). The continuing development of Magnéli phase titanium sub-oxides and Ebonex® electrodes. Electrochim. Acta.

[cit24] Smith J. R., Walsh F. C., Clarke R. L. (1998). Electrodes based on Magnéli phase titanium oxides:the properties and applications of Ebonex“materials”. J. Appl. Electrochem..

[cit25] Smith J. R., Walsh F. C., Clarke R. L. (1998). Electrodes based on Magnéli phase titanium oxides: the properties and applications of Ebonex® materials. J. Appl. Electrochem..

[cit26] Guo L., Jing Y., Chaplin B. P. (2016). Development and Characterization of Ultrafiltration TiO_2_ Magnéli Phase Reactive Electrochemical Membranes. Environ. Sci. Technol..

[cit27] Ellis K. (2004). *et al.*, The performance of Ebonex® electrodes in bipolar lead-acid batteries. J. Power Sources.

[cit28] Simičić M. V. (2017). *et al.*, Influence of non-stoichiometric binary titanium oxides addition on the electrochemical properties of the barium ferrate plastic-bonded cathode for super-iron battery. Electrochim. Acta.

[cit29] Toyoda M. (2009). *et al.*, Preparation of carbon-coated Magneli phases Ti_n_O_2n−1_ and their photocatalytic activity under visible light. Appl. Catal., B.

[cit30] Ekanayake S. A. (2024). *et al.*, Activated charcoal-mediated non-contact carbothermal reduction of TiO_2_ for controlled synthesis of Magnéli phase titanium suboxides. J. Mater. Chem. A.

[cit31] Jayashree S., Ashokkumar M. (2018). Switchable Intrinsic Defect Chemistry of Titania for Catalytic Applications. Catalysts.

[cit32] Berger O. (2022). Understanding the fundamentals of TiO_2_ surfaces. Part I. The influence of defect states on the correlation between crystallographic structure, electronic structure and physical properties of single-crystal surfaces. Surf. Eng..

[cit33] Kumar A., Barbhuiya N. H., Singh S. P. (2022). Magnéli phase titanium sub-oxides synthesis, fabrication and its application for environmental remediation: Current status and prospect. Chemosphere.

[cit34] Xu B. (2016). *et al.*, Structures, preparation and applications of titanium suboxides. RSC Adv..

[cit35] Wu X., Wang H., Wang Y. (2023). A Review: Synthesis and Applications of Titanium Sub-Oxides. Materials.

[cit36] Hejazi S. (2022). *et al.*, One-dimensional suboxide TiO_2_ nanotubes for electrodics applications. Electrochem. Commun..

[cit37] Rajaraman T. S., Parikh S. P., Gandhi V. G. (2020). Black TiO_2_: A review of its properties and conflicting trends. Chem. Eng. J..

[cit38] Walsh F. C., Wills R. G. A. (2010). The continuing development of Magnéli phase titanium sub-oxides and Ebonex® electrodes. Electrochim. Acta.

[cit39] Yang W. (2024). *et al.*, Preparation and Electrochemical Applications of Magnéli Phase Titanium Suboxides: A Review. Chem.–Eur. J..

[cit40] Dubrovinsky L. S. (2001). *et al.*, The hardest known oxide. Nature.

[cit41] Fujishima A., Zhang X., Tryk D. A. (2008). TiO_2_ photocatalysis and related surface phenomena. Surf. Sci. Rep..

[cit42] Liu L., Chen X. (2014). Titanium Dioxide Nanomaterials: Self-Structural Modifications. Chem. Rev..

[cit43] Nowotny M. K. (2008). *et al.*, Defect Chemistry of Titanium Dioxide. Application of Defect Engineering in Processing of TiO_2_-Based Photocatalysts. J. Phys. Chem. C.

[cit44] Nowotny M. K. (2005). *et al.*, Titanium vacancies in nonstoichiometric TiO_2_ single crystal. Phys. Status Solidi B.

[cit45] Lu Y. (2020). *et al.*, Spatial Heterojunction in Nanostructured TiO_2_ and Its Cascade Effect for Efficient Photocatalysis. Nano Lett..

[cit46] Zhuang G. (2020). *et al.*, Oxygen vacancies in metal oxides: recent progress towards advanced catalyst design. Sci. China Mater..

[cit47] Zhu K. (2020). *et al.*, The roles of oxygen vacancies in electrocatalytic oxygen evolution reaction. Nano Energy.

[cit48] Sarkar A., Khan G. G. (2019). The formation and detection techniques of oxygen vacancies in titanium oxide-based nanostructures. Nanoscale.

[cit49] Huang Y. (2020). *et al.*, Oxygen Vacancy Engineering in Photocatalysis. Sol. RRL.

[cit50] Pan X. (2013). *et al.*, Defective TiO_2_ with oxygen vacancies: synthesis, properties and photocatalytic applications. Nanoscale.

[cit51] Diebold U. (2003). Structure and properties of TiO_2_ surfaces: a brief review. Appl. Phys. A: Mater. Sci. Process..

[cit52] Pang C. L., Lindsay R., Thornton G. (2013). Structure of Clean and Adsorbate-Covered Single-Crystal Rutile TiO_2_ Surfaces. Chem. Rev..

[cit53] Sekiya T. (2004). *et al.*, Defects in Anatase TiO_2_ Single Crystal Controlled by Heat Treatments. J. Phys. Soc. Jpn..

[cit54] Liu X. (2016). *et al.*, Progress in Black Titania: A New Material for Advanced Photocatalysis. Adv. Energy Mater..

[cit55] Thompson T. L., Yates J. T. (2006). Surface Science Studies of the Photoactivation of TiO_2_ New Photochemical Processes. Chem. Rev..

[cit56] Parrino F. , *et al.*, 2 - Properties of titanium dioxide, in Titanium Dioxide (TiO_2_) and Its Applications, ed. F. Parrino and L. Palmisano, Elsevier, 2021, pp. 13–66

[cit57] Berger T. (2005). *et al.*, Light-Induced Charge Separation in Anatase TiO_2_ Particles. J. Phys. Chem. B.

[cit58] Henderson M. A. (2003). *et al.*, Insights into Photoexcited Electron Scavenging Processes on TiO_2_ Obtained from Studies of the Reaction of O_2_ with OH Groups Adsorbed at Electronic Defects on TiO_2_(110). J. Phys. Chem. B.

[cit59] Stoyanov E., Langenhorst F., Steinle-Neumann G. (2007). The effect of valence state and site geometry on Ti L3,2 and O K electron energy-loss spectra of TixOy phases. Am. Mineral..

[cit60] Dang Y., West A. R. (2019). Oxygen stoichiometry, chemical expansion or contraction, and electrical properties of rutile, TiO_2±*δ*_ ceramics. J. Am. Ceram. Soc..

[cit61] Henderson M. A. (2011). A surface science perspective on TiO_2_ photocatalysis. Surf. Sci. Rep..

[cit62] K Szot M. R., Speier W., Klusek Z., Besmehn A., Waser R. (2011). TiO_2_—a prototypical memristive material. Nanotechnology.

[cit63] Harada S., Tanaka K., Inui H. (2010). Thermoelectric properties and crystallographic shear structures in titanium oxides of the Magnèli phases. J. Appl. Phys..

[cit64] Bursill L. A., Hyde B. G. (1971). Crystal Structures in the {132} CS Family of Higher Titanium Oxides Ti_n_O_2n-1_. Acta Crystallogr., Sect. B:Struct. Sci., Cryst. Eng. Mater..

[cit65] Stoyanov E., Langenhorst F., Steinle-Neumann G. (2007). The effect of valence state and site geometry on Ti L_3,2_ and O K electron energy-loss spectra of Ti_x_O_y_ phases. Am. Mineral..

[cit66] Cox P. A. , Transition metal oxides: an introduction to their electronic structure and properties, Oxford university press, 2010, vol. 27

[cit67] Padilha A. C. M. (2016). *et al.*, Charge storage in oxygen deficient phases of TiO_2_: defect Physics without defects. Sci. Rep..

[cit68] Azor-Lafarga A. (2018). *et al.*, Modified Synthesis Strategies for the Stabilization of low n Ti_n_O_2n-1_ Magnéli Phases. Chem. Rec..

[cit69] Ok K. M. (2018). *et al.*, Effect of point and planar defects on thermal conductivity of TiO_2−*x*_. J. Am. Ceram. Soc..

[cit70] Le Page Y., Strobel P. (1982). Structural chemistry of the Magnéli phases Ti_n_O_2n−1_, 4 ≤ n ≤ 9: II. Refinements and structural discussion. J. Solid State Chem..

[cit71] Goodenough J. B. (1960). Direct Cation- -Cation Interactions in Several Oxides. Phys. Rev..

[cit72] Marezio M. (1973). *et al.*, Structural aspects of the metal-insulator
transitions in Ti_4_O_7_. J. Solid State Chem..

[cit73] Andersson S. (1960). *et al.*, The crystal structure of Ti_5_O_9_. Acta Chem. Scand..

[cit74] Bowden M. E. (1996). *et al.*, Improved powder diffraction patterns for the titanium suboxides Ti_n_O_2n−1_ (4 ≤ n ≤ 9). Powder Diffr..

[cit75] Strobel P., Le Page Y. (1982). Growth of Ti_9_O_17_ crystals by chemical vapor transport. J. Cryst. Growth.

[cit76] Lei W. (2022). *et al.*, Defect engineering of nanostructures: Insights into photoelectrochemical water splitting. Mater. Today.

[cit77] Fan Y. (2018). *et al.*, Preparation of monophasic titanium sub-oxides of Magnéli phase with enhanced thermoelectric performance. J. Eur. Ceram. Soc..

[cit78] Bak T., Nowotny J., Stranger J. (2010). Electrical properties of TiO_2_: equilibrium *vs.* dynamic electrical conductivity. Ionics.

[cit79] Woydt M. (2000). Tribological characteristics of polycrystalline Magnéli-typetitanium dioxides. Tribol. Lett..

[cit80] Maneeratana V. (2014). *et al.*, Original Electrospun Core-Shell Nanostructured Magnéli Titanium Oxide Fibers and their Electrical Properties. Adv. Mater..

[cit81] Kitada A. (2012). *et al.*, Selective Preparation of Macroporous Monoliths of Conductive Titanium Oxides Ti_n_O_2n–1_ (n = 2, 3, 4, 6). J. Am. Chem. Soc..

[cit82] Eder D., Kramer R. (2003). Stoichiometry of “titanium suboxide”. Phys. Chem. Chem. Phys..

[cit83] Fairhurst S. A. (1983). *et al.*, Intrinsic paramagnetic dimers in the magneli titanium oxides. J. Magn. Reson..

[cit84] Slipukhina I., Ležaić M. (2014). Electronic and magnetic properties of the Ti_5_O_9_ Magnéli phase. Phys. Rev. B:Condens. Matter Mater. Phys..

[cit85] Marezio M. (1972). *et al.*, Charge Localization at Metal-Insulator Transitions in Ti_4_O_7_ and V_4_O_7_. Phys. Rev. Lett..

[cit86] Backhaus-Ricoult M. (2012). *et al.*, Levers for Thermoelectric Properties in Titania-Based Ceramics. J. Electron. Mater..

[cit87] Eyert V., Schwingenschlögl U., Eckern U. (2004). Charge order, orbital order, and electron localization in the Magnéli phase Ti_4_O_7_. Chem. Phys. Lett..

[cit88] Watanabe M. (2009). Raman spectroscopy of charge-ordered states in Magnéli titanium oxides. Phys. Status Solidi C.

[cit89] Watanabe M., Ueno W. (2006). Raman study of order-disorder transition of bipolarons in Ti_4_O_7_. Phys. Status Solidi C.

[cit90] Keys L. K., Mulay L. N. (1967). Magnetic Susceptibility Measurements of Rutile and the Magnéli Phases of the Ti-O System. Phys. Rev..

[cit91] Mulay L. N., Danley W. J. (1970). Cooperative Magnetic Transitions in the Titanium-Oxygen System: A New Approach. J. Appl. Phys..

[cit92] Adamaki V. (2014). *et al.*, Manufacturing and characterization of Magnéli phase conductive fibres. J. Mater. Chem. A.

[cit93] Regonini D. (2012). *et al.*, AC electrical properties of TiO_2_ and Magnéli phases, Ti_n_O_2n−1_. Solid State Ionics.

[cit94] Shi X.-L., Zou J., Chen Z.-G. (2020). Advanced Thermoelectric Design: From Materials and Structures to Devices. Chem. Rev..

[cit95] Zhou X. (2018). *et al.*, Routes for high-performance thermoelectric materials. Mater. Today.

[cit96] Liu X. (2021). *et al.*, Controlling the Thermoelectric Properties of Nb-Doped TiO_2_ Ceramics through Engineering Defect Structures. ACS Appl. Mater. Interfaces.

[cit97] Pandey S. J. (2016). *et al.*, Modeling the Thermoelectric Properties of Ti_5_O_9_ Magnéli Phase Ceramics. J. Electron. Mater..

[cit98] Zhou D. (2022). *et al.*, Preparation of titanium-tantalum-oxygen composite thermoelectric ceramics through high-pressure and high-temperature method. J. Alloys Compd..

[cit99] Qian X., Zhou J., Chen G. (2021). Phonon-engineered extreme thermal conductivity materials. Nat. Mater..

[cit100] Lee H. (2016). *et al.*, Thermoelectric properties of in-situ plasma spray synthesized sub-stoichiometry TiO_2−*x*_. Sci. Rep..

[cit101] Vratny F., Micale F. (1963). Reflectance spectra of non-stoichiometric titanium oxide, niobium oxide, and vanadium oxide. Trans. Faraday Soc..

[cit102] Coufová P., Arend H. (1961). On the nature of colour centres in oxygen-deficient BaTiO_3_ single crystals. Trans. Faraday Soc..

[cit103] Han W.-Q., Zhang Y. (2008). Magnéli phases Ti_n_O_2n−1_ nanowires: Formation, optical, and transport properties. Appl. Phys. Lett..

[cit104] Zhou G. (2017). *et al.*, Ti^3+^ self-doped mesoporous black TiO_2_/graphene assemblies for unpredicted-high solar-driven photocatalytic hydrogen evolution. J. Colloid Interface Sci..

[cit105] Liu J. (2019). *et al.*, Pressure Dependence of Electrical Conductivity of Black Titania Hydrogenated at Different Temperatures. J. Phys. Chem. C.

[cit106] Malik H. (2020). *et al.*, Modelling and synthesis of Magnéli Phases in ordered titanium oxide nanotubes with preserved morphology. Sci. Rep..

[cit107] Niu M. (2015). *et al.*, Bandgap engineering of Magnéli phase Ti_n_O_2n−1_: Electron-hole self-compensation. J. Chem. Phys..

[cit108] Chen X. (2011). *et al.*, Increasing solar absorption for photocatalysis with black hydrogenated titanium dioxide nanocrystals. Science.

[cit109] Liu Y. (2018). *et al.*, Revealing the Relationship between Photocatalytic Properties and Structure Characteristics of TiO_2_ Reduced by Hydrogen and Carbon Monoxide Treatment. ChemSusChem.

[cit110] Fang W., Xing M., Zhang J. (2014). A new approach to prepare Ti^3+^ self-doped TiO_2_*via* NaBH_4_ reduction and hydrochloric acid treatment. Appl. Catal., B.

[cit111] Xing M. (2013). *et al.*, Self-doped Ti^3+^-enhanced TiO_2_ nanoparticles with a high-performance photocatalysis. J. Catal..

[cit112] Liu X. (2018). *et al.*, Bifunctional, Moth-Eye-Like Nanostructured Black Titania Nanocomposites for Solar-Driven Clean Water Generation. ACS Appl. Mater. Interfaces.

[cit113] Siracusano S. (2009). *et al.*, Preparation and characterization of titanium suboxides as conductive supports of IrO_2_ electrocatalysts for application in SPE electrolysers. Electrochim. Acta.

[cit114] Li X. (2010). *et al.*, Magneli phase Ti_4_O_7_ electrode for oxygen reduction reaction and its implication for zinc-air rechargeable batteries. Electrochim. Acta.

[cit115] Geng P., Chen G. (2016). Magnéli Ti_4_O_7_ modified ceramic membrane for electrically-assisted filtration with antifouling property. J. Membr. Sci..

[cit116] Takimoto D. (2019). *et al.*, Conductive Nanosized Magnéli-Phase Ti_4_O_7_ with a Core@Shell Structure. Inorg. Chem..

[cit117] Krishnan P., Advani S. G., Prasad A. K. (2012). Magneli phase Ti_n_O_2n−1_ as corrosion-resistant PEM fuel cell catalyst support. J. Solid State Electrochem..

[cit118] Martyanov I. N. (2010). *et al.*, Enhancement of TiO_2_ visible light photoactivity through accumulation of defects during reduction–oxidation treatment. J. Photochem. Photobiol., A.

[cit119] Lee Y.-W. (2013). *et al.*, Facile and catalytic synthesis of conductive titanium suboxides for enhanced oxygen reduction activity and stability in proton exchange membrane fuel cells. Int. J. Electrochem. Sci..

[cit120] Kolbrecka K., Przyluski J. (1994). Sub-stoichiometric titanium oxides as ceramic electrodes for oxygen evolution—structural aspects of the voltammetric behaviour of Ti_n_O_2n−1_. Electrochim. Acta.

[cit121] Gayen P. (2018). *et al.*, Electrocatalytic Reduction of Nitrate Using Magnéli Phase TiO_2_ Reactive Electrochemical Membranes Doped with Pd-Based Catalysts. Environ. Sci. Technol..

[cit122] Misal S. N. (2020). *et al.*, Modeling electrochemical oxidation and reduction of sulfamethoxazole using electrocatalytic reactive electrochemical membranes. J. Hazard. Mater..

[cit123] Nayak S., Chaplin B. P. (2018). Fabrication and characterization of porous, conductive, monolithic Ti_4_O_7_ electrodes. Electrochim. Acta.

[cit124] Jing Y., Guo L., Chaplin B. P. (2016). Electrochemical impedance spectroscopy study of membrane fouling and electrochemical regeneration at a sub-stoichiometric TiO_2_ reactive electrochemical membrane. J. Membr. Sci..

[cit125] Domaschke M. (2019). *et al.*, Magnéli-Phases in Anatase Strongly Promote Cocatalyst-Free Photocatalytic Hydrogen Evolution. ACS Catal..

[cit126] Kuroda Y. (2019). *et al.*, Templated Synthesis of Carbon-Free Mesoporous Magnéli-Phase Titanium Suboxide. Electrocatalysis.

[cit127] Yao S. (2017). *et al.*, Synthesis, characterization, and electrochemical performance of spherical nanostructure of Magnéli phase Ti_4_O_7_. J. Mater. Sci.: Mater. Electron..

[cit128] Vasilevskaia A. K. (2016). *et al.*, Formation of nonstoichiometric titanium oxides nanoparticles Ti_n_O_2n–1_ upon heat-treatments of titanium hydroxide and anatase nanoparticles in a hydrogen flow. Russ. J. Appl. Chem..

[cit129] Si P. (2021). *et al.*, Origin of Enhanced Electricity Generation on Magnéli Phase Titanium Suboxide Nanocrystal Films. ACS Appl. Energy Mater..

[cit130] Krishnan P., Advani S. G., Prasad A. K. (2012). Magneli phase Ti_n_O_2n−1_ as corrosion-resistant PEM fuel cell catalyst support. J. Solid State Electrochem..

[cit131] You S. (2016). *et al.*, Monolithic Porous Magnéli-phase Ti_4_O_7_ for Electro-oxidation Treatment of Industrial Wastewater. Electrochim. Acta.

[cit132] Moradi R., Groth K. M. (2019). Hydrogen storage and delivery: Review of the state of the art technologies and risk and reliability analysis. Int. J. Hydrogen Energy.

[cit133] Wang F. (2019). *et al.*, Formation mechanisms of interfaces between different Ti_n_O2_n−1_ phases prepared by carbothermal reduction reaction. CrystEngComm.

[cit134] Dewan M. A. R., Zhang G., Ostrovski O. (2009). Carbothermal Reduction of Titania in Different Gas Atmospheres. Metall. Mater. Trans. B.

[cit135] Zhu R. (2013). *et al.*, Magnéli phase Ti_4_O_7_ powder from carbothermal reduction method: formation, conductivity and optical properties. J. Mater. Sci.: Mater. Electron..

[cit136] Ostrovski O. (2010). *et al.*, Carbothermal Solid State Reduction of Stable Metal Oxides. Steel Res. Int..

[cit137] Koc R., Folmer J. S. (1997). Carbothermal synthesis of titanium carbide usingultrafine titania powders. J. Mater. Sci..

[cit138] Trellu C. (2018). *et al.*, Mineralization of organic pollutants by anodic oxidation using reactive electrochemical membrane synthesized from carbothermal reduction of TiO_2_. Water Res..

[cit139] Takeuchi T. (2017). *et al.*, Synthesis of Ti_4_O_7_ Nanoparticles by Carbothermal Reduction Using Microwave Rapid Heating. Catalysts.

[cit140] Huang S.-S. (2018). *et al.*, Synthesis of High-Performance Titanium Sub-Oxides for Electrochemical Applications Using Combination of Sol–Gel and Vacuum-Carbothermic Processes. ACS Sustainable Chem. Eng..

[cit141] Tsumura T. (2004). *et al.*, Formation of the Ti4O7 phase through interaction between coated carbon and TiO_2_. Desalination.

[cit142] Pang Q. (2014). *et al.*, Surface-enhanced redox chemistry of polysulphides on a metallic and polar host for lithium-sulphur batteries. Nat. Commun..

[cit143] Nagao M. (2020). *et al.*, Magneli-Phase Titanium Suboxide Nanocrystals as Highly Active Catalysts for Selective Acetalization of Furfural. ACS Appl. Mater. Interfaces.

[cit144] Geng X. (2023). *et al.*, Fabrication of high-performance Magnéli phase Ti4O7 ceramics by in-situ hot-pressed sintering in a single step. Mater. Today Commun..

[cit145] Liu M. (2021). *et al.*, Atom-economic synthesis of Magnéli phase Ti_4_O_7_ microspheres for improved sulfur cathodes for Li–S batteries. Nano Energy.

[cit146] He C. (2015). *et al.*, Direct synthesis of pure single-crystalline Magnéli phase Ti_8_O_15_ nanowires as conductive carbon-free materials for electrocatalysis. Nanoscale.

[cit147] Zhu G. (2015). *et al.*, Black Titania for Superior Photocatalytic Hydrogen Production and Photoelectrochemical Water Splitting. ChemCatChem.

[cit148] Gusev A. A. (2004). *et al.*, Conducting ceramic anodes based on titanium oxides. Chem. Sustainable Dev..

[cit149] Tang C., Zhou D., Zhang Q. (2012). Synthesis and characterization of Magneli phases: Reduction of TiO_2_ in a decomposed NH_3_ atmosphere. Mater. Lett..

[cit150] Gou H.-P., Zhang G.-H., Chou K.-C. (2017). Phase evolution and reaction mechanism during reduction–nitridation process of titanium dioxide with ammonia. J. Mater. Sci..

[cit151] Zhang X. (2014). *et al.*, Fabrication of Ti_4_O_7_ electrodes by spark plasma sintering. Mater. Lett..

[cit152] Ye J. (2015). *et al.*, Temperature effect on electrochemical properties of Ti_4_O_7_ electrodes prepared by spark plasma sintering. J. Mater. Sci.: Mater. Electron..

[cit153] Yu M. (2018). *et al.*, Magnéli phase titanium suboxides by Flash Spark Plasma Sintering. Scr. Mater..

[cit154] Wang L. (2021). *et al.*, Study on the fabricated non-stoichiometric titanium dioxide by *in situ* reduction with carbon powder *via* spark plasma sintering. J. Mater. Sci.: Mater. Electron..

[cit155] Conze S. (2015). *et al.*, Magnéli phases Ti_4_O_7_ and Ti_8_O_15_ and their carbon nanocomposites *via* the thermal decomposition-precursor route. J. Solid State Chem..

[cit156] Xu B. (2018). *et al.*, Flash synthesis of Magnéli phase (Ti_n_O_2n-1_) nanoparticles by thermal plasma treatment of H_2_TiO_3_. Ceram. Int..

[cit157] Ertekin Z., Tamer U., Pekmez K. (2015). Cathodic electrochemical deposition of Magnéli phases Ti_n_O_2n−1_ thin films at different temperatures in acetonitrile solution. Electrochim. Acta.

[cit158] Langlade C. (2002). *et al.*, Characterization of titanium oxide films with Magnéli structure elaborated by a sol–gel route. Appl. Surf. Sci..

[cit159] Matsuda M. (2021). *et al.*, Magnéli Ti_4_O_7_ thin film produced by stepwise oxidation of titanium metal foil. Scr. Mater..

[cit160] Sun S. (2016). *et al.*, Enhanced electrochemical performance of TiO_2_ nanotube array electrodes by controlling the introduction of substoichiometric titanium oxides. J. Alloys Compd..

[cit161] Vračar L. M. (2006). *et al.*, Electrocatalysis by nanoparticles – oxygen reduction on Ebonex/Pt electrode. J. Electroanal. Chem..

[cit162] Xu J. (2022). *et al.*, Insights into the electrooxidation of florfenicol by a highly active La-doped Ti_4_O_7_ anode. Sep. Purif. Technol..

[cit163] Luk'yanenko T. (2019). *et al.*, Design and properties of dimensionally stable anodes on Ebonex® substrate. Vopr. Khim. Khim. Tekhnol..

[cit164] Dong G., Yan L., Bi Y. (2023). Advanced oxygen evolution reaction catalysts for solar-driven photoelectrochemical water splitting. J. Mater. Chem. A.

[cit165] You B. (2019). *et al.*, Enhancing Electrocatalytic Water Splitting by Strain Engineering. Adv. Mater..

[cit166] Ge M. (2017). *et al.*, One-dimensional TiO_2_ Nanotube Photocatalysts for Solar Water Splitting. Advanced Science.

[cit167] Slavcheva E. (2005). *et al.*, Electrocatalytic activity of Pt and PtCo deposited on Ebonex by BH reduction. Electrochim. Acta.

[cit168] Stoyanova A. (2012). *et al.*, Oxygen evolution on Ebonex-supported Pt-based binary compounds in PEM water electrolysis. Int. J. Hydrogen Energy.

[cit169] Won J.-E. (2018). *et al.*, PtIr/Ti_4_O_7_ as a bifunctional electrocatalyst for improved oxygen reduction and oxygen evolution reactions. J. Catal..

[cit170] Paunović P. (2010). *et al.*, Co-Magneli phases electrocatalysts for hydrogen/oxygen evolution. Int. J. Hydrogen Energy.

[cit171] Jović B. M. (2016). *et al.*, Ni-(Ebonex-supported Ir) composite coatings as electrocatalysts for alkaline water electrolysis. Part II: Oxygen evolution. Int. J. Hydrogen Energy.

[cit172] Tian Z. (2019). *et al.*, Novel Black BiVO_4_/TiO_2−*x*_ Photoanode with Enhanced Photon Absorption and Charge Separation for Efficient and Stable Solar Water Splitting. Adv. Energy Mater..

[cit173] Wierzbicka E. (2019). *et al.*, Magnéli Phases Doped with Pt for Photocatalytic Hydrogen Evolution. ACS Appl. Energy Mater..

[cit174] Ďurovič M., Hnát J., Bouzek K. (2021). Electrocatalysts for the hydrogen evolution reaction in alkaline and neutral media. A comparative review. J. Power Sources.

[cit175] Eftekhari A. (2017). Electrocatalysts for hydrogen evolution reaction. Int. J. Hydrogen Energy.

[cit176] Xiong G. (2022). *et al.*, Au(111)@Ti6O11 heterostructure composites with enhanced synergistic effects as efficient electrocatalysts for the hydrogen evolution reaction. Nanoscale.

[cit177] Qiao J., Xiong Y. (2021). Electrochemical oxidation technology: A review of its application in high-efficiency treatment of wastewater containing persistent organic pollutants. J. Water Proc. Eng..

[cit178] Trellu C. (2018). *et al.*, Electro-oxidation of organic pollutants by reactive electrochemical membranes. Chemosphere.

[cit179] Panizza M., Cerisola G. (2009). Direct And Mediated Anodic Oxidation of Organic Pollutants. Chem. Rev..

[cit180] Geng P. (2015). *et al.*, Highly-Ordered Magnéli Ti_4_O_7_ Nanotube Arrays as Effective Anodic Material for Electro-oxidation. Electrochim. Acta.

[cit181] Lin H. (2021). *et al.*, Defect Engineering on a Ti_4_O_7_ Electrode by Ce^3+^ Doping for the Efficient Electrooxidation of Perfluorooctanesulfonate. Environ. Sci. Technol..

[cit182] Liang S. (2018). *et al.*, Electro-oxidation of tetracycline by a Magnéli phase Ti_4_O_7_ porous anode: Kinetics, products, and toxicity. Chem. Eng. J..

[cit183] Almassi S. (2019). *et al.*, Simultaneous Adsorption and Electrochemical Reduction of *N*-Nitrosodimethylamine Using Carbon-Ti_4_O_7_ Composite Reactive Electrochemical Membranes. Environ. Sci. Technol..

[cit184] Huang D. (2020). *et al.*, Amorphous Pd-Loaded Ti_4_O_7_ Electrode for Direct Anodic Destruction of Perfluorooctanoic Acid. Environ. Sci. Technol..

[cit185] Wang B. (2021). *et al.*, Simultaneous removal of multidrug-resistant Salmonella enterica serotype typhimurium, antibiotics and antibiotic resistance genes from water by electrooxidation on a Magnéli phase Ti_4_O_7_ anode. Chem. Eng. J..

[cit186] Wang Y. (2022). *et al.*, Electrooxidation of perfluorooctanesulfonic acid on porous Magnéli phase titanium suboxide Anodes: Impact of porous structure and composition. Chem. Eng. J..

[cit187] Becerril-Estrada V. (2020). *et al.*, Study of TiO_2_/Ti_4_O_7_ photo-anodes inserted in an activated carbon packed bed cathode: Towards the development of 3D-type photo-electro-Fenton reactors for water treatment. Electrochim. Acta.

[cit188] Bai H. (2017). *et al.*, Fabrication of Sc_2_O_3_-Magneli phase titanium composite electrode and its application in efficient electrocatalytic degradation of methyl orange. Appl. Surf. Sci..

[cit189] Dieckmann G. R., Langer S. H. (1998). Comparisons of Ebonex® and graphite supports for platinum and nickel electrocatalysts. Electrochim. Acta.

[cit190] Chen G., Betterton E. A., Arnold R. G. (1999). Electrolytic oxidation of trichloroethylene using a ceramic anode. J. Appl. Electrochem..

[cit191] Jing Y. (2018). *et al.*, The roles of oxygen vacancies, electrolyte composition, lattice structure, and doping density on the electrochemical reactivity of Magnéli phase TiO_2_ anodes. J. Mater. Chem. A.

[cit192] Tran C. (2011). *et al.*, Increased discharge capacity of a Li-air activated carbon cathode produced by preventing carbon surface passivation. Carbon.

[cit193] Jabbar Z. H., Esmail Ebrahim S. (2022). Recent advances in nano-semiconductors photocatalysis for degrading organic contaminants and microbial disinfection in wastewater: A comprehensive review. Environ. Nanotechnol., Monit. Manage..

[cit194] Rafiq A. (2021). *et al.*, Photocatalytic degradation of dyes using semiconductor photocatalysts to clean industrial water pollution. J. Ind. Eng. Chem..

[cit195] Nakata K., Fujishima A. (2012). TiO_2_ photocatalysis: Design and applications. J. Photochem. Photobiol., C.

[cit196] Zhao X. (2020). *et al.*, A direct oxygen vacancy essential Z-scheme C@Ti4O7/g-C3N4 heterojunctions for visible-light degradation towards environmental dye pollutants. Appl. Surf. Sci..

[cit197] Si P. (2022). *et al.*, Gradient Titanium Oxide Nanowire Film: a Multifunctional Solar Energy Utilization Platform for High-Salinity Organic Sewage Treatment. ACS Appl. Mater. Interfaces.

[cit198] Fujiwara K. (2014). *et al.*, Visible-light active black TiO_2_-Ag/TiO_*x*_ particles. Appl. Catal., B.

[cit199] Li L. H., Deng Z. X., Xiao J. X., Yang G. W. (2015). A metallic metal oxide (Ti5O9)-metal oxide (TiO_2_)nanocomposite as the heterojunction to enhance visible-light photocatalytic activity. Nanotechnology.

[cit200] Zaky A. M., Chaplin B. P. (2013). Porous Substoichiometric TiO_2_ Anodes as Reactive Electrochemical Membranes for Water Treatment. Environ. Sci. Technol..

[cit201] Qi Y. (2024). *et al.*, Electrochemical filtration for drinking water purification: A review on membrane materials, mechanisms and roles. J. Environ. Sci..

[cit202] Wang W. (2024). *et al.*, Enhanced treatment of p-nitrophenol and coking wastewater through electrochemical and electrochemical-ozonation coupling process utilizing a novel Ti4O7 reactive electrochemical membrane anode. J. Environ. Chem. Eng..

[cit203] Zaky A. M., Chaplin B. P. (2014). Mechanism of p-Substituted Phenol Oxidation at a Ti_4_O_7_ Reactive Electrochemical Membrane. Environ. Sci. Technol..

[cit204] Zaky A. M., Chaplin B. P. (2013). Porous Substoichiometric TiO_2_ Anodes as Reactive Electrochemical Membranes for Water Treatment. Environ. Sci. Technol..

[cit205] Santos M. C. (2016). *et al.*, Highly porous Ti_4_O_7_ reactive electrochemical water filtration membranes fabricated *via* electrospinning/electrospraying. AIChE J..

[cit206] Hayfield P. C. S. and Kuhn A., Development of a New Material: Monolithic Ti_4_O_7_ Ebonex Ceramic, Royal Society of Chemistry, 2007

[cit207] Liang S. (2018). *et al.*, Electrochemical inactivation of bacteria with a titanium sub-oxide reactive membrane. Water Res..

[cit208] Gupta V. (2020). *et al.*, Resistive Random Access Memory: A Review of Device Challenges. IETE Tech. Rev..

[cit209] Sawa A. (2008). Resistive switching in transition metal oxides. Mater. Today.

[cit210] Kwon D.-H. (2010). *et al.*, Atomic structure of conducting nanofilaments in TiO_2_ resistive switching memory. Nat. Nanotechnol..

[cit211] Strachan J. P. (2010). *et al.*, Direct Identification of the Conducting Channels in a Functioning Memristive Device. Adv. Mater..

[cit212] Hwan Kim G. (2011). *et al.*, Improved endurance of resistive switching TiO_2_ thin film by hourglass shaped Magnéli filaments. Appl. Phys. Lett..

[cit213] Banerjee W. (2017). *et al.*, Complementary Switching in 3D Resistive Memory Array. Adv. Electron. Mater..

[cit214] Doo Seok Jeong R. T., Katiyar R. S., Scott J. F., Kohlstedt H., Petraru A., Hwang C. S. (2012). Emerging memories: resistive switchingmechanisms and current status. Rep. Prog. Phys..

[cit215] Hosaka T. (2020). *et al.*, Research Development on K-Ion Batteries. Chem. Rev..

[cit216] Li L. (2022). *et al.*, Advanced Multifunctional Aqueous Rechargeable Batteries Design: From Materials and Devices to Systems. Adv. Mater..

[cit217] Wang Z. (2020). *et al.*, Resistive switching materials for information processing. Nat. Rev. Mater..

[cit218] Styles M. J. (2012). *et al.*, A furnace and environmental cell for the *in situ* investigation of molten salt electrolysis using high-energy X-ray diffraction. J. Synchrotron Radiat..

[cit219] Tao X. (2014). *et al.*, Strong Sulfur Binding with Conducting Magnéli-Phase Ti_n_O_2n–1_ Nanomaterials for Improving Lithium–Sulfur Batteries. Nano Lett..

[cit220] Sabbaghi A. (2023). *et al.*, Magneli Ti_4_O_7_ nanotube arrays as a free-standing efficient interlayer for fast-delivery and high-energy density lithium-sulfur batteries. J. Alloys Compd..

[cit221] Han W.-Q., Wang X.-L. (2010). Carbon-coated Magnéli-phase Ti_n_O_2n−1_ nanobelts as anodes for Li-ion batteries and hybrid electrochemical cells. Appl. Phys. Lett..

[cit222] Lee G.-W. (2019). *et al.*, Magnéli Phase Titanium Oxide as a Novel Anode Material for Potassium-Ion Batteries. ACS Omega.

[cit223] Wei H. (2017). *et al.*, Chemical Bonding and Physical Trapping of Sulfur in Mesoporous Magnéli Ti_4_O_7_ Microspheres for High-Performance Li–S Battery. Adv. Energy Mater..

[cit224] Franko C. J., Sadighi Z., Goward G. R. (2022). Ti_4_O_7_-Enhanced Carbon Supports to Stabilize NaO_2_ in Sodium-Oxygen Batteries. J. Phys. Chem. C.

[cit225] Lee S. (2018). *et al.*, Magnéli-Phase Ti_4_O_7_ Nanosphere Electrocatalyst Support for Carbon-Free Oxygen Electrodes in Lithium–Oxygen Batteries. ACS Catal..

[cit226] Dao V.-D., Vu N. H., Yun S. (2020). Recent advances and challenges for solar-driven water evaporation system toward applications. Nano Energy.

[cit227] Lin Y. (2019). *et al.*, Solar steam generation based on the photothermal effect: from designs to applications, and beyond. J. Mater. Chem. A.

[cit228] Xu X. (2024). *et al.*, Full-spectrum-responsive Ti4O7-PVA nanocomposite hydrogel with ultrahigh evaporation rate for efficient solar steam generation. Desalination.

[cit229] Qiu X. (2022). *et al.*, Interface Engineering of a Ti4O7 Nanofibrous Membrane for Efficient Solar-Driven Evaporation. ACS Appl. Mater. Interfaces.

[cit230] Zhang Z. (2018). *et al.*, Emerging hydrovoltaic technology. Nat. Nanotechnol..

[cit231] Qing Y. (2021). *et al.*, Ti3+ self-doped dark TiO_2_ nanoparticles with tunable and unique dielectric properties for electromagnetic applications. J. Mater. Chem. C.

[cit232] Gardon M. (2013). *et al.*, New procedures for building-up the active layer of gas sensors on flexible polymers. Surf. Coat. Technol..

[cit233] Canillas M. (2013). *et al.*, Physico-chemical properties of the Ti_5_O_9_ Magneli phase with potential application as a neural stimulation electrode. J. Mater. Chem. B.

[cit234] Liu H. J. (2022). *et al.*, A high strength and conductivity bulk Magnéli phase Ti4O7 with superior electrochemical performance. Ceram. Int..

[cit235] Oladipupo P., Raman A., Pekny J. F. (2023). Minimum production scale for economic feasibility of a titanium dioxide plant. J. Adv. Manuf. Process..

[cit236] Takeda O., Ouchi T., Okabe T. H. (2020). Recent Progress in Titanium Extraction and Recycling. Metall. Mater. Trans. B.

[cit237] Alhashimi H. A., Aktas C. B. (2017). Life cycle environmental and economic performance of biochar compared with activated carbon: A meta-analysis. Resour.,
Conserv. Recycl..

[cit238] Takeuchi T. (2017). *et al.*, Synthesis of Ti4O7 Nanoparticles by Carbothermal Reduction Using Microwave Rapid Heating. Catalysts.

[cit239] Arif A. F. (2017). *et al.*, Highly conductive nano-sized Magnéli phases titanium oxide (TiO_*x*_). Sci. Rep..

[cit240] Ma J. (2023). *et al.*, Porous Magnéli phase obtained from 3D printing for efficient anodic oxidation process. Chem. Eng. J..

[cit241] Yang Y. (2017). *et al.*, Discovery and ramifications of incidental Magnéli phase generation and release from industrial coal-burning. Nat. Commun..

[cit242] McDaniel D. K. (2019). *et al.*, Pulmonary exposure to Magnéli phase titanium suboxides results in significant macrophage abnormalities and decreased lung function. Front. Immunol..

[cit243] Yang Y. (2015). *et al.*, Importance of a Nanoscience Approach in the Understanding of Major Aqueous Contamination Scenarios: Case Study from a Recent Coal Ash Spill. Environ. Sci. Technol..

[cit244] Kononenko V., Drobne D. (2019). In Vitro Cytotoxicity Evaluation of the Magnéli Phase Titanium Suboxides (Ti_x_O_2x−1_) on A549 Human Lung Cells. Int. J. Mol. Sci..

[cit245] Jemec Kokalj A. (2019). *et al.*, The first comprehensive safety study of Magnéli phase titanium suboxides reveals no acute environmental hazard. Environ. Sci.: Nano.

